# Grain amaranth genes coding for an RNA-binding and a small, unknown function protein, respectively, enhance thermotolerance when overexpressed in *Arabidopsis thaliana*

**DOI:** 10.1007/s12298-025-01696-x

**Published:** 2026-01-08

**Authors:** Gabriela Cabrales-Orona, Alejandra Reyes-Rosales, Norma A. Martínez-Gallardo, Lino Sánchez-Segura, José Luis Cabrera-Ponce, Octavio Martínez de la Vega, Paola A. Palmeros-Suárez, John Paul Délano-Frier

**Affiliations:** 1 Departamento de Biotecnología y Bioquímica, Centro de Investigación y de Estudios Avanzados del IPN, Unidad Irapuato, Km 9.6 del Libramiento Norte Carretera Irapuato-León, 36500 Irapuato, GTO México; 2 Departamento de Ingeniería Genética, Centro de Investigación y de Estudios Avanzados del IPN, Unidad Irapuato, Km 9.6 del Libramiento Norte Carretera Irapuato-León, 36500 Irapuato, GTO México; 3 Departamento de Producción Agrícola, Centro Universitario de Ciencias Biológicas y Agropecuarias, Universidad de Guadalajara, 45200 Zapopan, JAL México; 4 Department of Plant and Soil Science, Institute of Genomics for Crop Abiotic Stress Tolerance (IGCAST), Texas Tech University, Lubbock, TX 79409 USA

**Keywords:** Grain amaranth, Heat stress, Heat shock, Intrinsically disordered proteins, RNA-binding proteins, Thermotolerance, Unknown-function genes

## Abstract

Recent findings suggest that unknown function genes may contribute to grain amaranths’ capacity to thrive under stressful conditions. In the present study, the overexpression of two grain amaranth unknown function genes, i.e., *AhHAB4-PAI-1* and *Ah2880*, in *Arabidopsis thaliana* supported this premise by enhancing their thermotolerance. These genes were identified in stress-exposed *Amaranthus hypochondriacus* plants and were also induced by heat shock (HS) conditions. Accordingly, both transgenic *A. thaliana* lines recovered from HS exposure that was near-lethal to untransformed plants. Transcriptional and microscopic analyses indicated that enhanced HS tolerance in both transgenic plant lines occurred by yet to be defined mechanisms that followed strikingly different time-course activation patterns, as evinced by: (i) the accumulation of RNA florescence signals, hypothetically representative of stress granules, which reached their highest intensity in the midst of HS conditions, in *AhHAB4-PAI-1* OE plants, and at the initial recovery phase, in *Ah2880* OE plants, and (ii) the analysis of transcriptomic data, which revealed a clear difference in the nature, timing and abundance of differentially expressed genes (DEGs) recorded in both OE plants during the HS and recovery stages. Collectively, most DEGs were representative of known heat stress-related responses, predominantly DNA repair, alternative splicing, chromatin remodeling, protein stabilization/degradation/modification, autophagy, cell wall and membrane alterations, ribosomal and organellar responses, high molecular weight complex formation and activation of stress-associated transcription factors and phytohormone signaling. This study’s results highlight the potential use of unknown function genes for the generation of highly heat stress-resistant plants, which may occur through contrasting protective mechanisms.

**Supplementary Information:**

The online version contains supplementary material available at 10.1007/s12298-025-01696-x.

## Introduction

Plants are sessile organisms uncapable of escaping from the various and usually harmful stress conditions that they encounter during their life span in an increasingly warming world. Daily and seasonal temperature fluctuations having sufficient range, rate and duration to produce heat stress are among the most damaging conditions affecting plants (Liu et al. 2013; Nahar et al. 2022). A recent IPCC report (Hoegh-Guldberg et al. 2018) predicted that a significant increase in average global temperature will inevitably affect worldwide crop and ecosystem productivity (Dusenge et al. 2019; Li et al. 2019; Ortiz-Bobea et al. 2019) with estimated yield reductions in wheat, rice, maize and soybean ranging between 3.1 and 7.4% with every 1 °C increase in ambient temperature above 28 °C (Challinor et al. 2014; Zhao et al. 2017).

The plant’s response to heat stress is activated by largely unknown heat sensors that initiate a rapid and reversible accumulation of signaling-related factors such as Ca^2+^, H_2_O_2_, nitric oxide, phytohormones and various others. These further contribute to heat stress perception through the promoted function of heat shock factors (HSFs) and heat shock proteins (HSPs), MAPK and calcium-dependent kinases signaling cascades and additional regulatory processes that include microRNAs and small interfering RNAs (Hayes et al. 2021; Pagano et al. 2021; Zuo et al. 2021; Pokhrel and Meyers 2022). Further responses to heat stress involve an overall transcriptional and post-translational regulation associated with the rapid assembly of stress granules (SGs) and other cytosolic focal points. SGs are phase-separated, membrane-less, cytoplasmic ribonucleoprotein assemblies that are generated to promote cell survival. Their formation implicates the condensation of translationally static mRNAs, ribosomal complexes and several other components that depend on the presence of different RNA-binding proteins, in addition to intrinsically disordered proteins and related chaperones (Kosmacz et al. 2019; Campos-Melo et al. 2021; Maruri-López et al. 2021; Zhu et al. 2022). Heat stress also activates alternative RNA splicing (Rosenkranz et al. 2022), a widespread reorganization of the protein biogenesis, transport, modification and degradation network (Trösch et al. 2022), a remodeling of the cytosolic translation machinery (Beine-Golovchuk et al. 2018), cell-cycle arrest, modified photosynthetic performance, cell wall reorganization, cuticular wax synthesis and carbohydrate modifications (Xiang and Rathinasabapathi 2022).

The genus *Amaranthus* consists of *ca.* 70 annual herbaceous plants with C4 photosynthesis (Kadereit et al. 2003; Das 2016) that are mostly cultivated as vegetable and/or grain crops (Achigan-Dako et al. 2014; Joshi et al. 2018; Hoidal et al. 2019; Sarker and Oba 2019). Three grain amaranths species are known: *Amaranthus cruentus*, *A. hypochondriacus* and *A. caudatus*. They are greatly valued for their highly nutritional, protein-rich seeds that have added nutraceutical properties, while their leaves may be also used as a vitamin-rich vegetable source (Venskutonis and Kraujalis 2013; De Ron et al. 2017; Hoidal et al. 2019). Grain amaranths, which are native of the American continent (Kietlinski et al. 2014; Wu and Blair 2017; Stetter et al. 2020 and references therein), are considered to be climate-resilient plants due to their demonstrated tolerance to drought, salinity, above-average heat and ultraviolet irradiance. They can also recover rapidly from severe defoliation, insect and/or pathogen damage and thrive in poor soils and low-input agricultural systems. This property has been associated with various anatomical, physiological and biochemical adaptations (Brenner et al. 2000; Reyes-Rosales et al. 2023 and references therein).

Data from a large-scale transcriptomic analysis of *A. hypochondriacus* plant leaves disclosed that several orphan and/or unknown function genes changed their expression levels in response to diverse stress conditions (Délano-Frier et al. 2011). Subsequent over-expression of a number of these genes in *Arabidopsis thaliana* suggested their active participation in mechanisms linked to (a)biotic stress tolerance (Cabrales-Orona and Délano-Frier 2021; Cabrales-Orona et al. 2022). The present study further expands this information by describing the results derived from the characterization of two of these intriguing genes in *A. thaliana* plants. They code for an RGG motif-containing and hyaluronan/mRNA binding protein (i.e., AhHAB4-PAI-1) and a small unknown protein (i.e., Ah2880), respectively. Similar to other RNA binding proteins, RGG motif-containing proteins have been proposed to be involved in the regulation of mRNA stability and posttranscriptional regulation of gene expression, in addition to their possible association with SGs during the plant’s adaptation to osmotic or drought stress (Huang et al. 2000; Ambrosone et al. 2015; Marondedze 2020; Marondedze et al. 2020; Muleya and Marondedze 2020). Ah2880 is a small, 161 amino acid (aa), unknown function protein predicted to have an N-terminal 58 aa α-helix, followed by a disordered structure constituted mostly by a random coil interspaced with five short (4 aa) helixes. A previous report showed that *Ah2880* expression is developmentally regulated in leaves of *A. cruentus*, whereas it was found to respond in a species-specific manner to diverse (a)biotic stresses and exogenous phytohormone applications (Cabrales-Orona and Délano-Frier 2021). This information was expanded by the results of the present study, which revealed that a significantly higher rate of recovery from a heat-shock (HS) treatment was provided by the overexpression of the *AhHAB4-PAI-1* and *Ah2880* in transgenic *A. thaliana* plants. Additional transcriptomic data provided possible mechanisms associated with the HS tolerance observed and also supported the growing evidence linking unknown function proteins to the increased tolerance to high temperatures, severe water-deficit and excessive soil salt contents that characterizes grain amaranths. They also suggest that these climate resilient plants may be used as a model to generate heat-resistant crops able to readily adapt to the rapidly changing climatic conditions of the planet.

## Materials and methods

### Plant material

*Arabidopsis thaliana* plants, ecotype Columbia, used for the generation of transgenic plants, were germinated and grown following standard procedures, as previously described (Palmeros-Suárez et al. 2015). Briefly, seeds were surface sterilized, were washed thrice with sterile distilled and de-ionized (dd) water and submerged in water for 48 h at 4 °C. Germination of vernalized seeds was performed on 100 × 15 mm petri dishes containing 0.1 × Murashige and Skoog (MS) media (Murashige and Skoog 1962) and incubated in growth chambers operating under controlled conditions of fluorescent white light (PAR, 70 to 110 μmol m^−2^ s^−1^), photoperiod (16 h light/8 h dark) and temperature (22 ± 1 °C). Ten days after germination, plants were transferred to 125 mL plastic pots filled with 60 g of a soil mixture composed of three parts Sunshine Mix 618 3TM (Sun-Gro Horticulture, Bellevue, WA, USA), one part loam, two parts mulch, one part vermiculite (SunGro Hort) and one part perlite (Termolita S.A., Nuevo León, México) and fertilized with a 20:10:20 N: P: K, plus micronutrients, solution (Peters Professional; Scotts-Sierra Horticultural Products, Marysville, OH, USA). All subsequent plant manipulation prior to experimentation was performed in the growth chambers described above.

### Identification of the genes coding for a hyaluronan and mRNA binding protein (AhHAB4-PAI-1) and for a small unknown function protein (Ah2880) in *A. hypochondriacus*

The whole gene and mRNA sequences of the grain amaranth *AhHAB4-PAI-1* and *Ah2880* genes were retrieved from the *A. hypochondriacus* genome (Phytozome 13, v2.1, accession numbers AH018279 and AH015341, respectively). The intronic and exonic regions of both genes were determined directly via the Phytozome platform (Goodstein et al. 2012). A MatInspector Release professional 8.0.5 program (Genomatix), was used to detect regulatory elements in the *ca.* 1600 bp promoter region of both genes. The proteins encoded by the open reading frames (ORFs) of these genes cDNAs were deduced with the aid of ExpasyTranslate platform (https://web.expasy.org/translate/). The AhHAB4-PAI-1 and Ah2880 protein sequences were used to perform BLASTp searches against different plant genomes within the NCBI platform. Multiple alignment with amino acid sequences of related proteins in other plant species, based on the NCBI taxonomy, was used to construct the phylogenetic trees. These were generated with MEGA software (version 11.0) using the maximum like-hood method based on the JTT matrix-based model (Jones et al. 1992).

### Molecular modeling of the AhHAB4-PAI-1 and Ah2880 proteins

Modeling of both proteins was performed by entering the amino acid sequence, in FASTA format, into the Iterative Threading ASSEmbly Refinement or I-TASSER server platform. The link predicted the most viable 3D structures which were modeled and visualized using the PYMOL software (http://pymol.sourceforge.net) (Roy et al. 2010). All websites mentioned in Sections. “Identification of the genes coding for a hyaluronan and mRNA binding protein (AhHAB4-PAI-1) and for a small unknown function protein (Ah2880) in A. hypochondriacus” and “Molecular modeling of the AhHAB4-PAI-1 and Ah2880 proteins”, and others below, were constantly accessed in a period comprising the years 2019 to 2022.

### Generation of *AhHAB4-PAI-1* and *Ah2880 *overexpressing (OE) *Arabidopsis thaliana* transgenic plants

The isolation of the full-length cDNA and subsequent generation of the *AhHAB4-PAI-1* and *Ah2880* OE transgenic plants was performed as follows: Total RNA samples (1 μg) from leaves of *A. hypochondriacus* were reverse-transcribed to generate the first-strand cDNA as previously described (Palmeros-Suárez et al. 2015). Aliquots of these reactions (2 μL) were directly used as template in all subsequent PCR reactions in the presence of 100 pmol each of specific primers. The 1051 bp and 483 bp open reading frames of the *AhHAB4-PAI-1* and the *Ah2880* genes, respectively, were directly PCR amplified from *A. hypochondriacus* cDNA using specific primers (Table S1) and purified using a standardized procedure (*Protocol* kit; Qiagen, Germatown, MD, USA). After sequence confirmation, the full-length cDNAs of the *AhHAB4-PAI-1* and *Ah2880* genes were cloned in the pCR8/GW/TOPO TA vector (Invitrogen, Carlsbad, CA, USA). This was followed by the LR reaction, catalyzed by the Clonase Enzyme Mix (Invitrogen), required to transfer them into the pB7WG2D destination vector (Cambia, Canberra, Australia), under the control of 35S CaMV promoter, which was subsequently used to transform chemo-competent *Escherichia coli* TOP10 cells (Invitrogen). These, were selected on the basis of acquired spectinomycin resistance (at 50 µg/mL). The binary constructs were extracted and purified following the manufacturer’s instructions (Invitrogen). After confirmation of their correct orientation and size by sequencing (Instituto de Biotecnología de la UNAM, Cuernavaca, Morelos, México), the constructs were electroporated into *Agrobacterium tumefaciens* strain GV2260. After incubating for 48 h at 28 °C on LB media spiked with rifampicin (25 µg/mL) and spectinomycin (100 mg/L), they were used to transform *A. thaliana* plants by a modified floral dip method (Martinez-Trujillo et al. 2004).

Seeds of the transformed plants were germinated and grown to maturity under controlled growing conditions, as described above. One-week old T1 transgenic *A. thaliana* plants were selected by growth on MS medium containing N-acetyl-L-phosphinothricin (PPT) (20 μg/mL). T2 seeds were collected from individual transformants (T1) and plated again on the selection medium to determine PPT-resistant vs. PPT-sensitive plants segregation ratios. Homozygous T3 and T4 transgenic plants produced no PPT-sensitive seedlings from seeds of T2 plants. All further experimentation with the *AhHAB4-PAI-1* and *Ah2880* OE lines was performed using T4 plants homozygous for the transgenes. Three independent T_4_ homozygous transgenic lines of the *AhHAB4-PAI-1* (i.e., L1, L12 and L13; Fig. S1A) and *Ah2880* (i.e., L9, L12 and L13; Fig. S1B) OE plants were generated for subsequent experimentation. They had low, medium and high levels of the respective transgenes. These were calculated relative to trace background levels in untransformed wild-type (WT) plants using the 2^−ΔΔC^_T_ comparative cycle threshold method (Livak and Schmittgen 2001) as described previously (Palmeros-Suárez et al. 2015).

### Generation of *pAh2880::GUS A. thaliana* plants and histochemical β‑glucuronidase (GUS) staining assay

A 1596 bp DNA fragment covering the 5’flanking region of the *A. hypochondriacus Ah2880* gene was PCR-amplified from *A. hypochondriacus* genomic DNA. The pairs of primers used in the PCR reaction are listed in Table S1. The amplicon was double-strand sequenced to verify its authenticity, as above. Using the Gateway technology previously described, it was inserted first into the pCR8/GW/TOPO TA vector and then into the pFAST-G04 vector harboring the *E. coli uidA* β-glucuronidase (*GUS*) reporter gene. This vector was electroporated into *A. tumefaciens* GV2260 cells, which were selected by incubation for 48 h and 28 °C on LB media supplemented with rifampicin (50 mg/L) and spectinomycin (100 mg/L). Transformed agrobacteria were subsequently used for *A. thaliana* transformation as described before. Seeds of T4 homozygous *A. thaliana* transgenic plant lines harboring the p*Ah2880::GUS* construct were germinated and grown as described before.

GUS histochemical staining was performed in diverse organs of transgenic *A. thaliana* plants harboring the *pAh2880::GUS* fusion construct at different development stages (5 and 35 day-old plants). The protocol employed for the visualization of the blue-stained organ sections indicative of GUS activity in plants grown in optimal conditions or subjected to different HS exposure times (see below) has been described previously (Cabrales-Orona et al. 2022). The images of blue-colored plant organs and plants at different developmental stages/times of HS exposure were recorded using a ZEISS Stemi 2000-C stereo microscope (Carl Zeiss, Oberkochen, Germany). GUS-stained organs and plants shown in this study were obtained from representative experiments that were repeated twice with similar results.

### Generation of *GFP::Ah2880 *plants and subcellular localization of the Ah2880 protein in* A. thaliana*

Plasmids used to express the *Ah2880* coding sequence N-terminally fused to the green fluorescent protein (GFP) under the control of the CaMV 35S promoter were constructed using the Gateway Cloning technology (Invitrogen), as described above. Coding sequences were amplified as PCR products via nested PCR with the same primers used for the generation of the OE plants (Table S1). After validation by sequencing, the entry clone was inserted into the pK7WGF2 destination vector in order to fuse the *Ah2880* sequence N-terminally to GFP (Karimi et al. 2002). These vectors were electroporated into *A. tumefaciens* GV2260 cells, which were selected and subsequently used for *A. thaliana* transformation as described above. Transformed plants were grown to maturity under controlled ambient conditions, as mentioned, and the seeds were harvested at maturity. One-week old T1 transgenic *A. thaliana* plants were selected by growth in MS medium containing PPT (20 μg/mL) in addition to the emission of GFP-related fluorescence, corroborated under a Zeiss Primo Star fluorescence microscope (Carl-Zeiss GmbH, Oberkochen, Germany) and the PCR amplification of the GFP marker gene using specific primers (Table S1). Seeds of T4 homozygous *A. thaliana* transgenic plant lines differing in *GFP*::*Ah2880* transgene dosage (i.e., L1, L8, and L44; Fig. S1C) were germinated and grown as described above.

Fluorescent images of roots and leaves were obtained from transgenic plants corresponding to lines 8 and 44 (Fig. S1C). They were recorded from roots and leaves of 3- and/or 35-day old plants maintained under optimal conditions, and from leaves of plants subjected to a HS treatment at 45 °C for 11 h and after A 1-day recovery period (see below). Root images were obtained using a confocal laser scanning microscope LSM 800 (Carl Zeiss). Propidium iodide (PI) at 0.01 mg/mL was used as a counterstain to highlight root cell morphology. GFP and PI were excited using the 488 nm and 514 nm laser lines, respectively, of an Argon laser. GFP emission was filtered with a 500–550-nm band pass (BP) filter and PI emission was filtered with a 575-nm long pass (LP) filter. *A. thaliana* leaves were dissected from the petiole and were fixed with 4% paraformaldehyde (Sigma-Aldrich, St. Louis MO, USA) dissolved in a 0.1 M phosphate buffer (PB), pH 7.2 for 24 h. The samples were washed three times with PB for 20 min and covered with Clear-Mount PIPES buffer (Electron Microscopy Science, Hatfield, PA, USA). The leaves were mounted on glass slides and covered with high-performance Gold Seal cover glasses (Thermo Scientific, Portsmouth, NH, USA) and observed in a multiphoton microscope (LSM 880–NLO, Carl Zeiss) equipped with a Ti:Sapphire laser (Chameleon Vision II, COHERENT, Santa Clara, CA, USA; tuning range: 690–1060 nm). The samples were observed and analysed with an immersion objective 60 × /1.4 Oil DIC, NA ∞/0.17, Zeiss Plan NEOFLUAR. The GFP was excited at 488 nm with 2.4% of laser power and emission was recorded at 492–535 nm. The chlorophyll was excited at 633 nm with 2.47% of laser power and the emission, from 660 to 711 nm, was recorded. All micrographs were captured in CZI format at 1024 × 1024 pixels and RGB color.

### HS assays in transgenic *A. thaliana* plants

The three transgenic plant lines for each transgene mentioned above were used to perform HS stress experiments, as defined by Geange et al. (2021). These were done using sets of 20 three-weeks old plants of WT plants and three lines each of the transgenic *AhHAB4-PAI-1* or *Ah2880* OE plants. HS experiments were also performed with transgenic *A. thaliana* plants overexpressing the N-terminal *GFP::Ah2880* construct (lines 8 and 44; Fig. S1C), together with their respective WT controls. For the HS experiments, the plants were placed for 22 h in a growth chamber set at 45 °C and under constant illumination. They were subsequently allowed to recover in a growth room kept under optimal conditions (see above) for seven days, time after the survival rate of the treated plants was registered. Additional HS treatments were performed in order to collect samples for subsequent reporter gene, microscopic and transcriptomic analysis (see below). The GUS reporter gene analysis was performed using plants subjected to HS for 1, 3, 5.5, 9 and 11 h (represented as HS-1 to HS-11). The microscopic analysis was performed using leaves from plants subjected to HS-11 and allowed to recover for 1 day, or R-1, under optimal growth conditions. The transcriptomic data was generated from RNA extracted form leaves of plants at HS-1, HS-5 and HS-11 and at R-1 and R-3. Sets of 20 experimental plants were used for each sampling point.

### Multiphoton microscopy to determine cell membrane integrity and RNA/DNA localization

*A. thalian*a leaves were dissected from the petiole and fixed with a 4% formaldehyde (Sigma-Aldrich) dissolved in phosphate buffer (PB) at 0.1 M, pH 7.2 for 24 h. The samples were cleared with ethanol/PB solution at 90% (*v*/*v*) and incubated for 24 h. Subsequently, the samples were washed thrice, for 30 min, with PB. To stain the cellular membranes, the samples were treated with a Synaptored C2 (Sigma-Aldrich,) solution (1 µL from a Synaptored C2 stock solution in 100 µL of PB) for 1.5 h at 4 °C in the dark, and subsequently rinsed thrice with PB. Subsequent RNA staining in the samples was performed by incubating in an orange acridine (OA; Sigma-Aldrich) solution (2 µL from an OA stock solution in 400 µL of PB), for 30 min at 4 °C in the dark. The samples were washed with PB for 20 min, and were then contra-stained to visualize DNA by incubating with a 4′,6-diamidino-2-phenylindole dihydrochloride (DAPI; Sigma-Aldrich) solution, prepared by mixing 5 µL of a DAPI stock solution in 400 µL of PB, for 40 min at 4 °C in the dark. Finally, the samples were washed thrice with PB for 20 min and covered with mounting medium prepared with PIPES buffer (Electron Microscopy Science, Hatfield, PA, USA).

The stained leaves were mounted on glass slides and covered with high-performance Gold Seal cover glasses (Thermo Scientific) and observed in a multiphoton microscope (LSM 880–NLO, Zeiss, Oberkochen, Germany) equipped with a Ti: Sapphire laser (Chameleon Vision II, COHERENT, Santa Clara, CA, USA; tuning range: 690–1,060 nm). The samples were observed and analysed with an immersion objective 60 × /1.4 Oil DIC, NA ∞/0.17, Zeiss Plan NEOFLUAR. The Synaptored C2 was excited with HeNe laser at 543 nm with 3.0% of power and the emission from 641–732 nm was recorded. OA was excited with an argon laser at 488 nm with 0.12% power, and the emission from 553 to 638 nm was recorded. DAPI was excited with the chameleon laser tuned at 710 nm with 3.0% of power and detected at 403–465 nm. All micrographs were captured in CZI format at 1024 × 1024 pixels and RGB colour.

### RNA-seq library construction and analysis

RNA extraction was performed as described previously (Palmeros-Suárez et al. 2015). Total RNA was extracted from leaves of *A. thaliana* of the two OE plant lines subjected to the different HS or R treatments, as described above. The leaves of 10 plants per treatment were used for each of the two biological replicates of the HS+R experiments performed. RNA purity and concentration were determined using a NanoDrop 2000 apparatus as instructed (ThermoFisher Scientific; Waltham, MA, USA). The integrity of the purified RNA was assessed through RNA integrity numbers (RIN) for each sample using the Bioanalyzer RNA 6000 Nano assay (Agilent; Santa Clara, CA, USA) provided by LabSerGen (Laboratorio Nacional de Genómica, Irapuato, Gto.; México). Pure and integral samples of total RNA were shipped to Novogene (https://en.novogene.com/ accessed on: 07 August 2022) for library construction, sequencing, and mapping to the *A. thaliana* reference genome, as described below.

Quantified libraries were pooled and sequenced on Illumina platforms, according to effective library concentration and data amount. The procedures performed by Novogene Co., Ltd, as well as a full report for each library were obtained. All clean reads from the 30 libraries were individually mapped to the TAIR10 release of the *A. thaliana* genome. Briefly, Bowtie2 (version 2.2.5) was employed to map the reads to the genome. Samtools (version 1.6) allowed the conversion of the sam to the bam format and the ordering for position to the reference genome. Picard (version 2.0.1) tools were employed to eliminate duplicate reads. Finally, the Subread (version 2.0.0) package was used to obtain the counts per gene for each one of the 30 libraries sequenced. The number of clean reads that mapped to the genome in the 30 libraries had a mean of 2,657,050. All statistical analyses were performed in R (R Core Team, 2013) (version 4.2.2) running under macOS Monterey 12.5.1. For Differential Gene Expression (DGE) analyses, the “edgeR” package (version 3.40.0) was employed. The subsequent strategy employed to analyze the large number of significant results in several different contrasts, and to reduce the complexity of the interpretation is described in detail in the Supplemental Experimental Procedures contained in the Supplemental Information file.

#### Statistical analysis

All the experiments were performed using a completely randomized design with at least 10 plants per genotype. Data generated were analyzed using a one-way analysis of variance (ANOVA) to examine the effect that the overexpression of these genes had on the survival of transgenic *A. thaliana* plants subjected to HS conditions. The means were compared using the Tukey–Kramer test to identify statistically significant differences between them. The statistical analysis was performed with the aid of R (http://r-project.org/) Rstudio (https://www.rstudio.com) and JMT Pro13 (jmp.com) statistical software.

## Results

### Sequence and domain architecture of the *AhHAB4-PAI-1* and *Ah2880* genes

Genes coding for an uncharacterized *A. hypochondriacus* hyaluronan and mRNA binding protein (AhHAB4-PAI-1) and an unknown function protein (Ah2880) were identified and characterized. Sequence alignment of the mRNA and the genomic contig sequences revealed that the *AhHAB4-PAI-1* gene is constituted by 4152 bp in which six embedded exons of 175, 307, 133, 159, 57 and 177 bp, respectively, combine to generate an ORF of 1,900 bp coding for a 356 aa protein with distinct ARN-binding and nuclear localization domains (Fig. S2). Likewise, the 1160 bp *Ah2880* gene contains four exons of 93, 71, 187 and 135 bp, respectively, that combine into a 486 bp ORF coding for a 161 aa protein. The 5’UTR and 3’UTR regions of both genes are also included (Fig. S3).

**Fig. 1:**
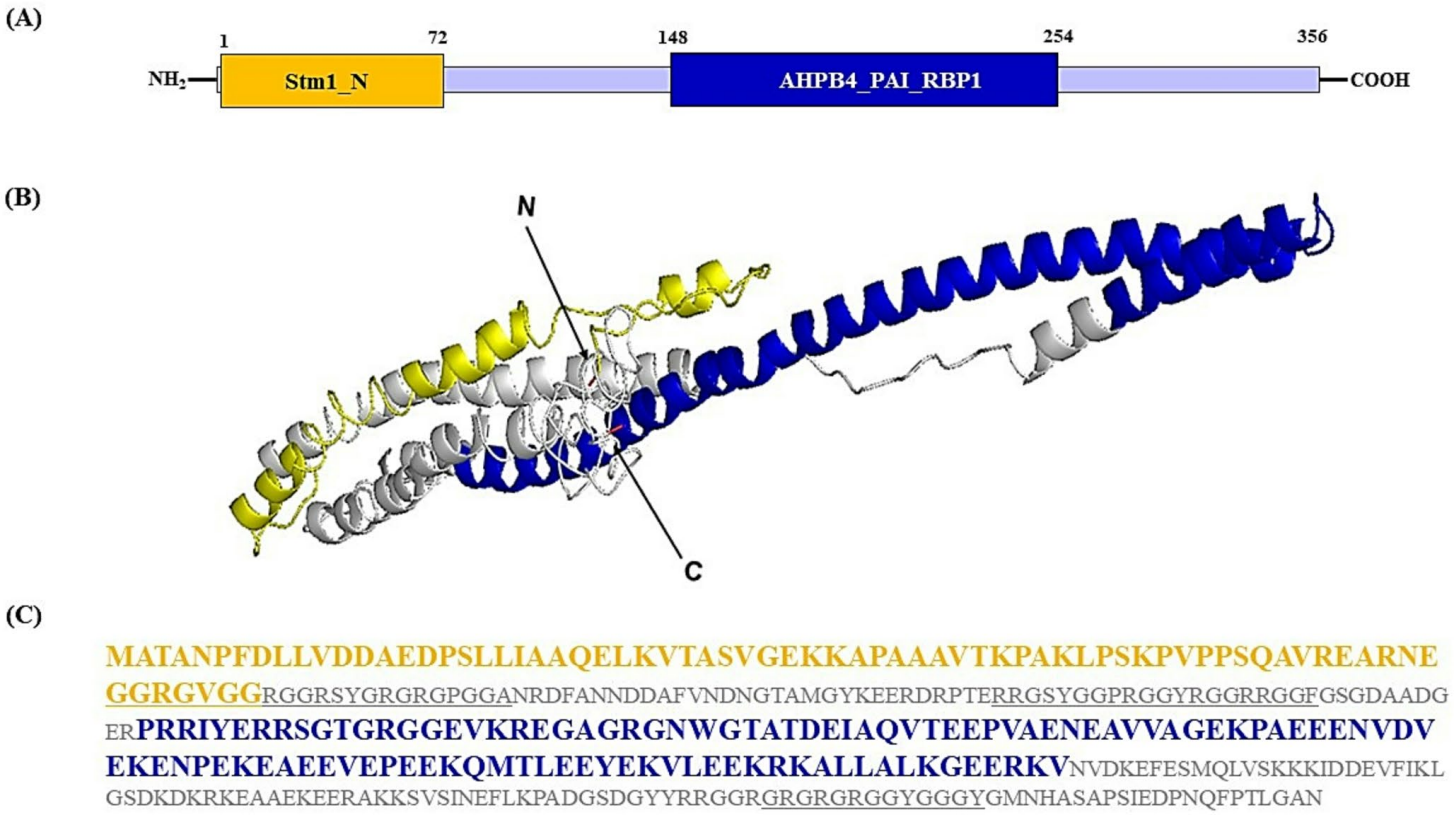
Structural characterization of the AhHAB4-PAI-1 protein. **A** Diagram showing the length of the AhHAB4-PAI-1 protein and the localization of the Stm1 and CDC13 nucleic acid-binding and hyaluronan binding motifs, respectively. **B** The molecular modeling of shows the Stm1 at the N-terminal end of the protein, colored yellow, followed by the CDC13 domain, colored blue. The model was generated using the tools described in https://zhanglab.ccmb.med.umich.edu/I-TASSER. **C** The 356-long amino acid sequence of the AhHAB4-PAI-1 protein. The segments corresponding to the Stm1 and CDC13 domains are colored in yellow and blue, respectively. The RGG/RG nucleic acid binding domains are underlined

The AhHAB4-PAI-1 protein contains an N-terminal region also found in Stm1, a G4 quadraplex and purine motif triplex nucleic acid-binding protein. It also harbors an HAB4-PAI-1 hyaluronan-binding domain that can recognize the type 1 plasminogen activating inhibitor (PAI) binding domain that regulates mRNA stability (Huang et al. 2000; Heaton et al. 2001). The Stm1 and HAB4-PAI-1 domains are localized at positions 1–72 and 148–254, respectively (Figs. 1A and C). In addition, this protein has three characteristic arginine-glycine-glycine (RGG) boxes, clustered together into RGG/RG motifs and C-terminal KR patches (Fig. S2C), which are predicted to facilitate protein-nucleic acids interactions (Thandapani et al. 2013; Ostendorp et al. 2022). Further bioinformatic analyses (https://zhanglab.ccmb.med.umich.edu/I-TASSER/output/S553256/) predicted a highly ordered 3D structure in which α-helices are predominant (Fig. 1B).

Ah2880 is a small, unknown function protein whose predicted secondary structure includes a predominant 58 aa α-helix, localized practically at the start of the N-terminal end. The rest of the protein is assembled as a disordered random coil (Cabrales-Orona and Délano-Frier 2021). Interestingly, a glutamine-rich segment within the central part of the Ah2880 protein resembles polyQ regions found in proteins having biologically-relevant prion-like domains (PrLDs, Fig. S3C; Jung et al. 2020).

### Phylogeny of the AhHAB4-PAI-1 and Ah2880 proteins

A phylogenetic tree (Fig. S4A and S4B) showed that the AhHAB4-PAI-1 and Ah2880 proteins from *A. hypochondriacus* are grouped together with other uncharacterized proteins present in related *Beta vulgaris*, *Spinacea oleracea* and *Chenopodium quinoa.* No sequences homologous to Ah2880 were detected in *A. thaliana,* whereas AhHAB4-PAI-1 shared a low degree of identity (maximum 50.8%) with hyaluronan/ mRNA-binding domain proteins and other RNA-binding proteins in *A. thaliana* (not shown). The trees also indicated that these proteins shared a low degree of homology with proteins present in other dicot plants species.

### In silico analysis of the promoter regions of the *AhHAB4-PAI-1 *and *Ah2880* genes

The results of an in silico analysis of the promoter regions of these two genes are shown in Table S2. Interestingly, several of the most prominent elements found in them, e.g., DNA-binding One Zinc Finger, or DOF, have been reported in genes associated with diverse heat stress responses in various plant models. Also, the abundance of some other motifs in the promoter region of the *AhHAB4-PAI-1* gene, e.g., the GT-1 motif and Sequences Over-Represented in Light-Induced was relevant considering that they are also found in the promoters of genes closely related to heat stress and high light responses in plants.

### GUS and GFP reporter-aided localization of *Ah2880* gene expression during development and in response to HS in transgenic *A. thaliana* plants

**Fig. 2:**
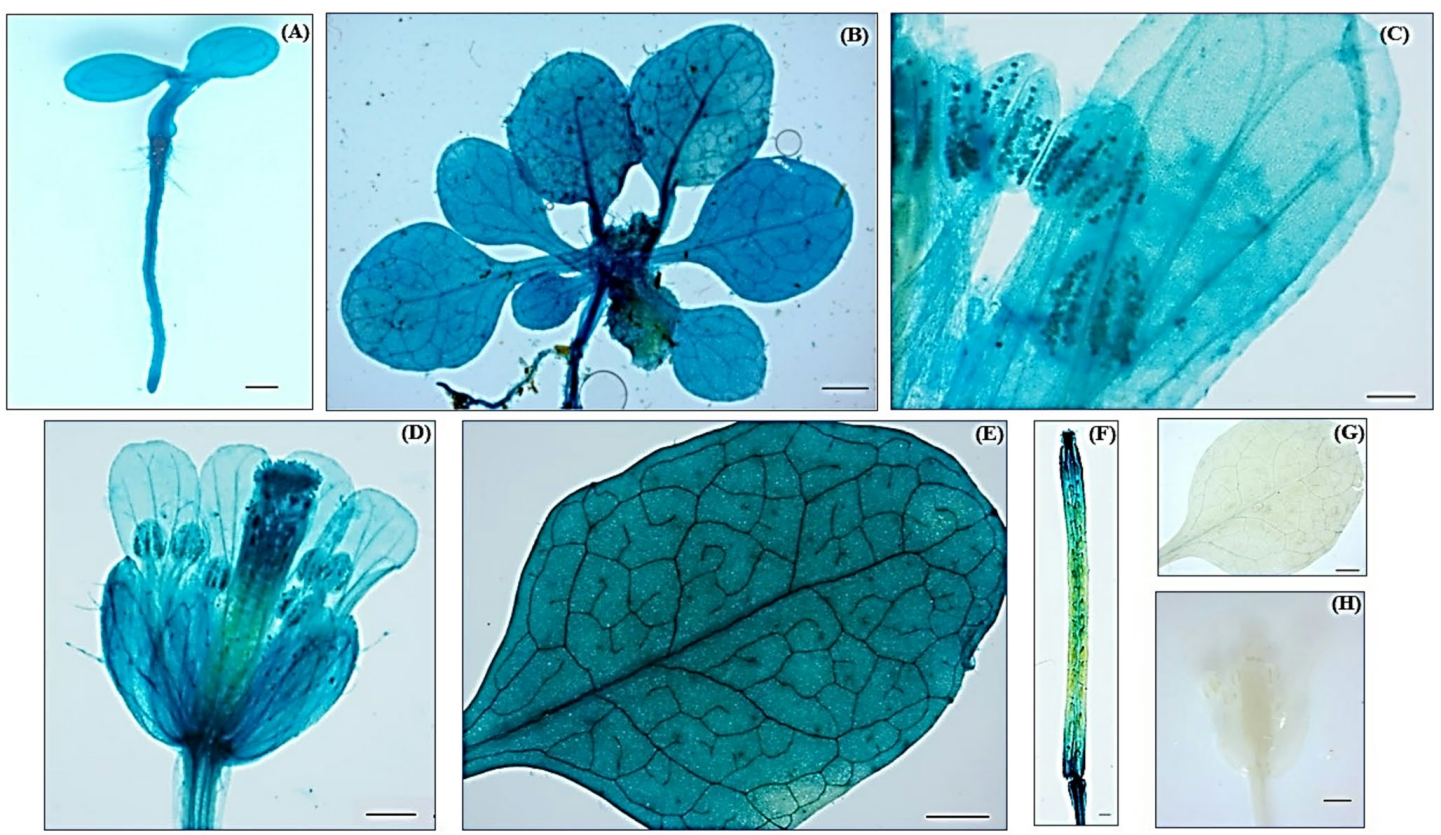
Histochemical assay of β-glucuronidase (GUS) activity in transgenic *Arabidopsis thaliana* plants expressing the *Ah2880* promoter-GUS fusion gene. The images show the detection of activated *GUS* reporter gene activation in **A** germinated seedlings, **B** adult plants, **C**, **D**, and **F** stamens, pistil and siliques of flowering plants, respectively, and **E** leaf vasculature. Images of leaves (**G**) and flowers (**H**) of untransformed WT controls are shown. Scale bars = 100 µM in A, E and F and 50 µm in B, C, D, F, G and H

**Fig. 3:**
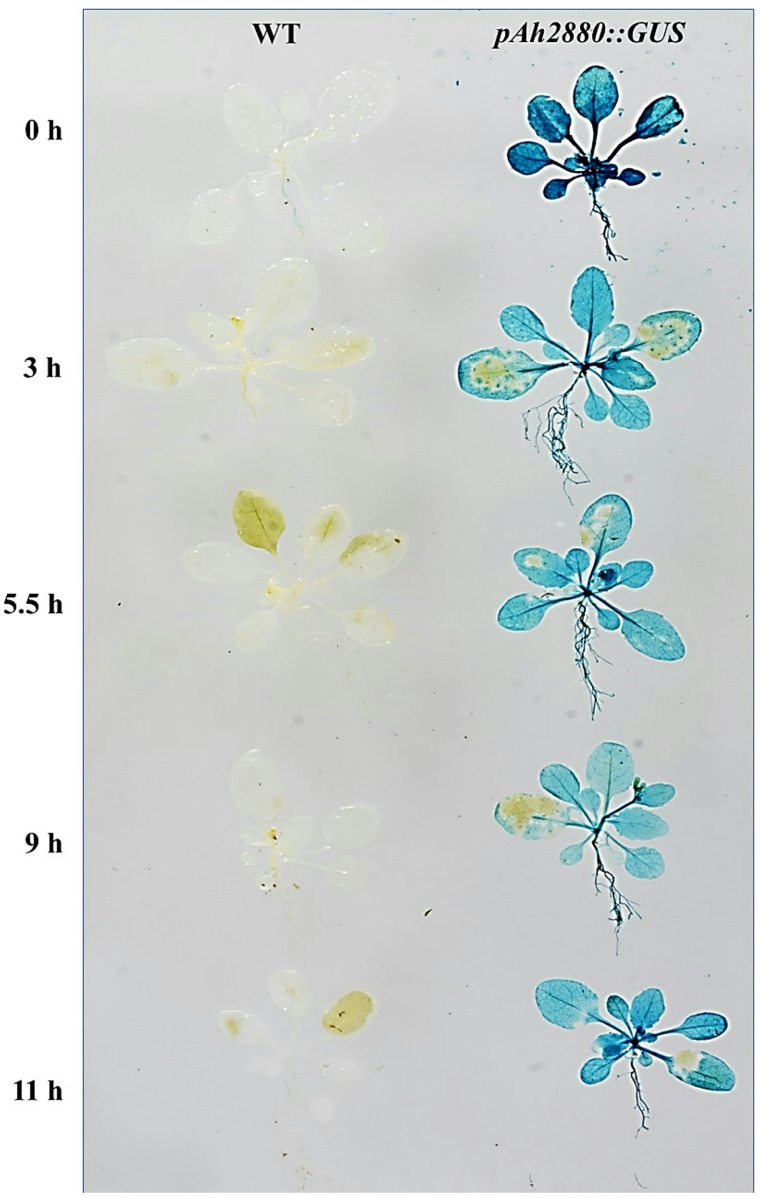
Histochemical assay of β-glucuronidase (GUS) activity in transgenic *Arabidopsis thaliana* plants expressing the *Ah2880* promoter-GUS fusion gene in response to heat shock. Broad GUS activity was detected in **A** transgenic, *pAh2880-GUS* plants maintained in optimal growing conditions. The GUS signal remained extensively expressed, but with a reduced intensity, in *pAh2880-GUS* plants exposed to HS, at 45 °C, for **B** 3 h, **C** 5.5 h, **D** 9 h and **E** 11 h. Scale bars = 50 µM in all images

**Fig. 4:**
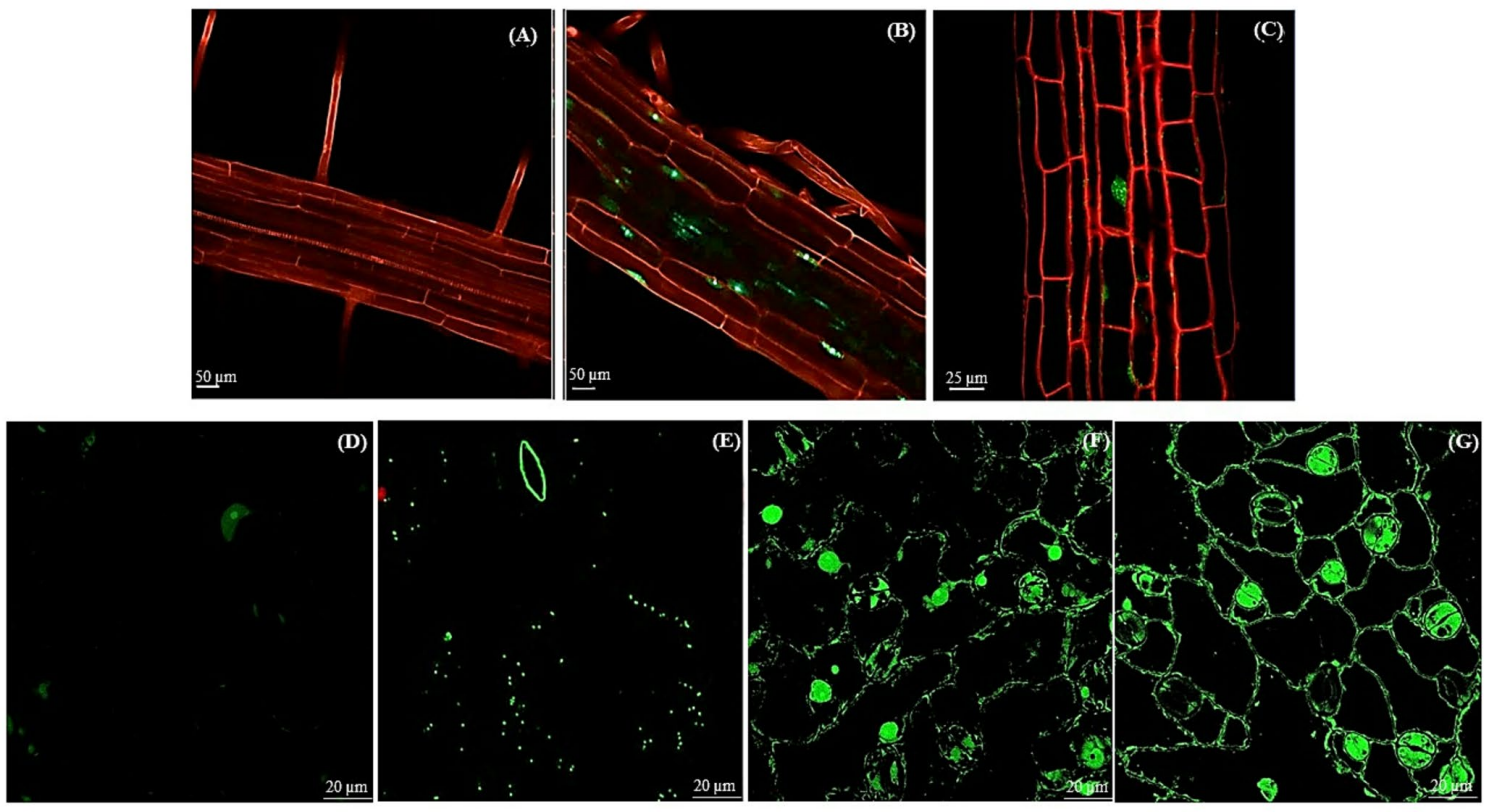
GFP fluorescence emission in roots and leaves of transgenic *Arabidopsis thaliana* plants expressing the N-terminal *eGFP*::*Ah2880* fusion gene. GFP emission against a background of propidium iodide-stained tissues in cells of the main root (**B** and **C**). Image of a propidium iodide-stained main root of an untransformed WT control plant is shown in (**A**). The GFP signal was sparsely distributed in the nuclei of a few mesophyll (**D**), and epidermal and stomatal guard cells (**E**) of leaves of untreated marker plants. An increase of GFP-Ah2880 signals was greatly increased in (**F**) plants exposed to heat shock (HS), at 45 °C, for 11 h, where the GFP fluorescence was mainly detected in nuclei and membranes and in (**G**) plants sampled 1 day after recovery from HS, where the GFP signal was detected in membranes and chloroplasts. Scale bars = 50 µm in (**A**) and (**B**); 25 µm in (**C**) and 20 µm in (D), (E), (F), and (G)

The prevalence of certain elements in the promoter region of the *Ah2880* gene (Table S2) was in agreement with the widespread tissue and organ expression pattern observed in transgenic plant lines harboring the GUS marker gene (Fig. 2). Thus, elements able to recognize homeobox proteins, similar to those present in L1 proteins and DOF TFs known to participate in the regulation of several vegetative and reproductive development processes, were prominent. Moreover, the extended expression pattern of the *GUS* marker gene, although less intense than the basal *GUS* response observed in leaves of untreated marker plants, was maintained during the first half of the HS treatment, consistent with the presence of motifs associated with thermotolerance genes (Table S2; Fig. 3). The sub-cellular localization of the GFP-Ah2880 marker in roots was predominantly observed in the nuclei of cortical cells and in the vascular cylinder (Fig. 4B and C). In contrast to the extended activation of the *Ah2880* promoter observed in untreated *A. thaliana* leaves, the signal of the Ah2880-GFP marker proteins was restricted to the nuclei and nucleolus of relatively few epidermal and stomatal guard cells (Fig. 4D and E). However, greatly increased GFP fluorescence was detected at HS-11, mostly in the nuclei, nucleolus and cell membranes (Fig. 4F). At R-1, the GFP signal became more compartmentalized and was localized mainly in the membranes and young guard cells, probably reflecting the differentiation of new stomatal structures in plants recovering from HS. A more pronounced localization in the cell membrane was also observed (Fig. 4G).

### Determination of HS tolerance in *AhHAB4-PAI-1* and *Ah2880* OE *A. thaliana* plants

**Fig. 5:**
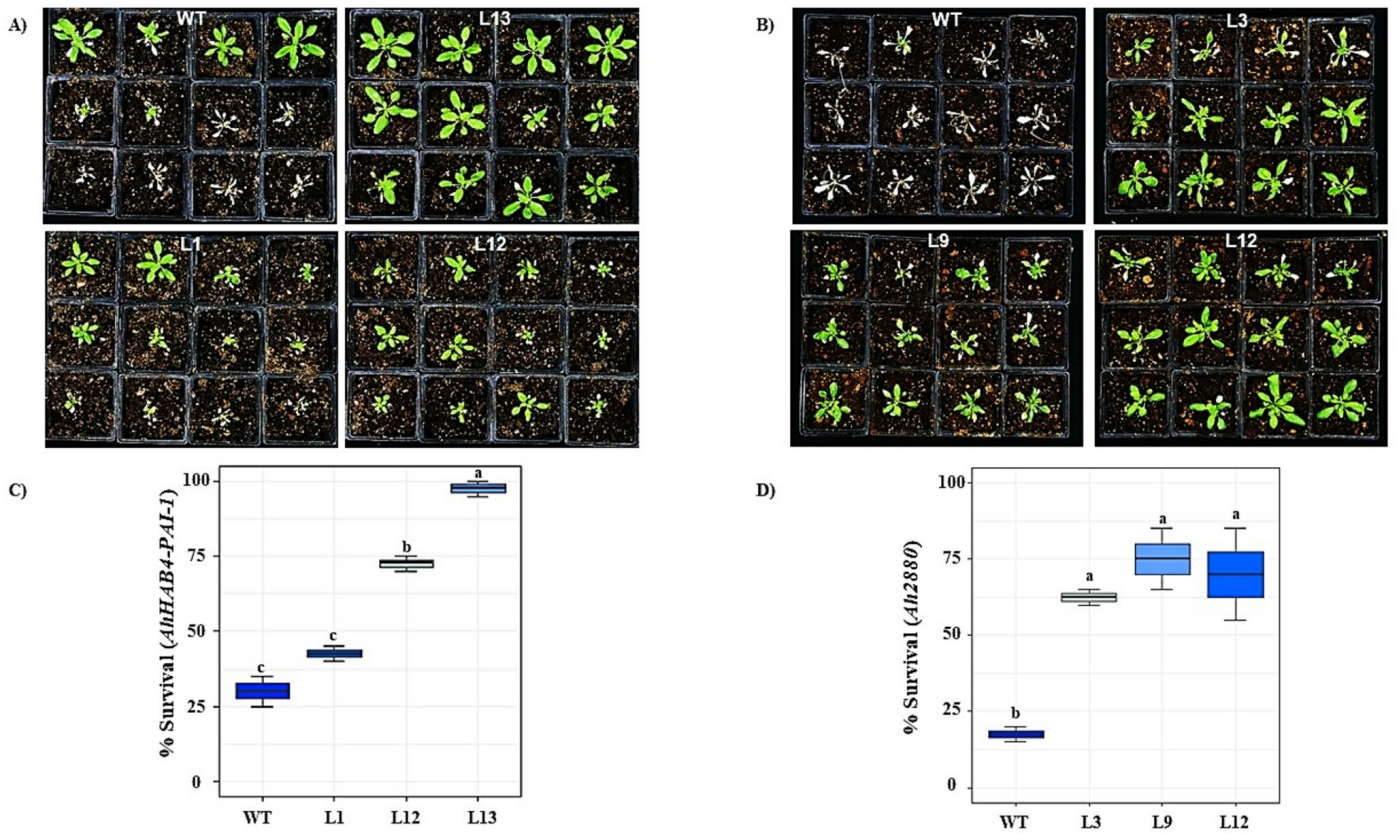
The overexpression of *AhHAB4-PAI-1* or *Ah2880* in *Arabidopsis thaliana* plants increases their capacity to recover from heat shock conditions recreated inside a growth chamber. Images of untransformed (WT) and 3 independent lines of transgenic plants overexpressing (OE) the (**A**) *AhHAB4-PAI-1* (lines 1, 12 and 13) and the (**B**) *Ah2880* (lines 3, 9 and 12) grain amaranth genes, respectively, as recorded after a 7-day recovery period in optimal growing conditions (22 °C; 16 h/ 8 h light: dark photoperiod) following a 22 h heat-shock treatment at 45 °C under constant illumination. The percent of WT and transgenic plants that fully recovered from the heat shock applied is shown for the (**C**) *AhHAB4-PAI-1* and (**D**) *Ah2880* OE lines. Different letters over the box-and-whisker plots represent statistically significant differences at *P* ≤ 0.05 (One-way ANOVA, Tukey Kramer test; *n* = 20). The results shown are those obtained from a representative experiment that was repeated thrice, with similar results

Transgenic plants overexpressing either the *AhHAB4-PAI-1* or *Ah2880* genes (Fig. S1A and B) showed a significantly higher capacity to recover from HS than WT plants (Fig. 5). The survival percentage to HS was significantly higher in most transgenic lines tested. Recovery was transgene dose-dependent in *AhHAB4-PAI-1* OE plants, whereas the HS recovery rates detected in the *Ah2880* OE plants were largely transgene dosage-independent (Fig. 5C and D).

### DNA-RNA abundance and localization patterns in leaves of transgenic *A. thaliana* plants overexpressing the *AhHAB4-PAI-1* or *Ah2880* genes during HS and subsequent recovery

**Fig. 6:**
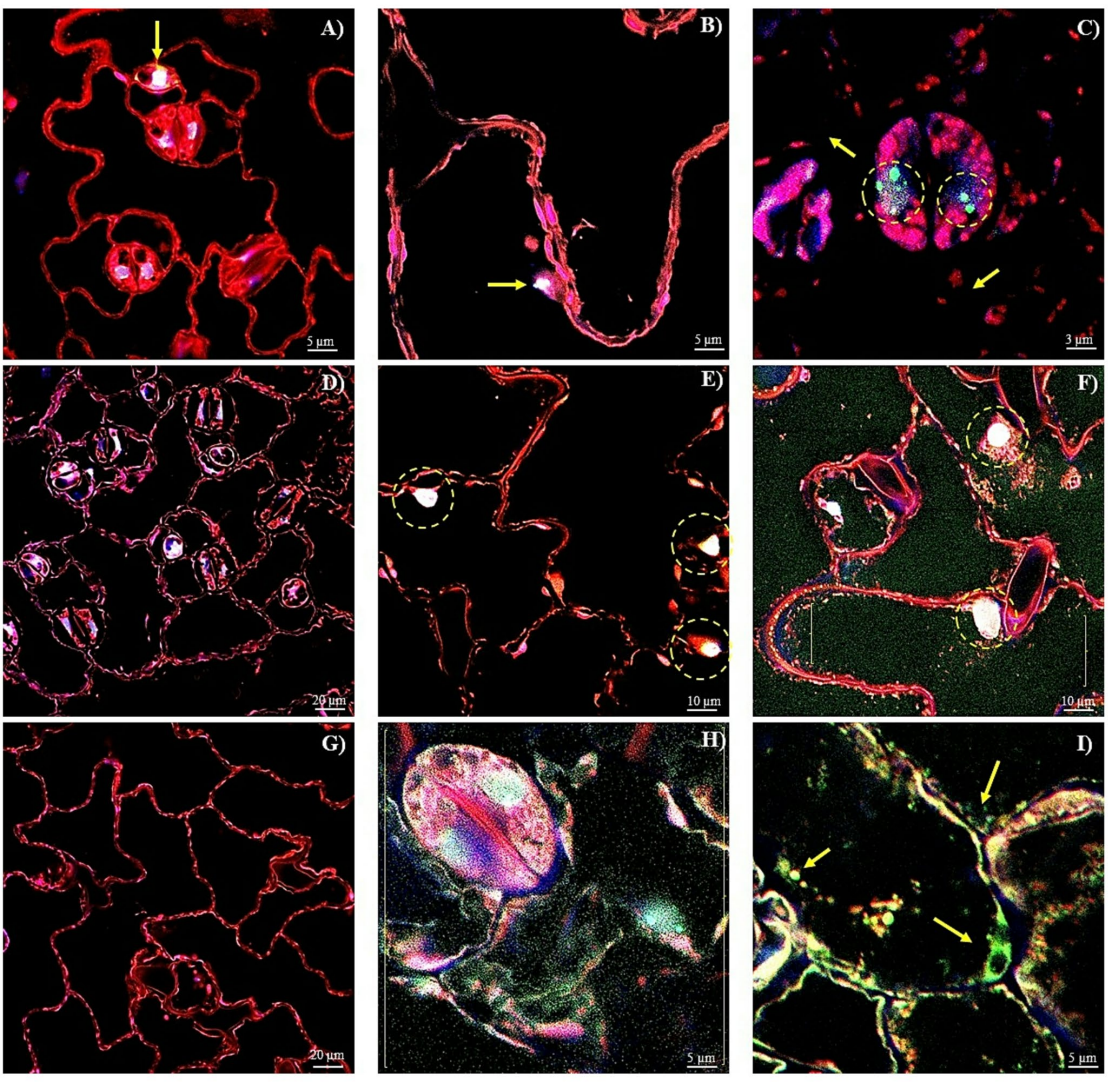
RNA accumulation and GFP localization patterns in leaf cells of *AhHAB4-PAI-1 and Ah2880* overexpressing (OE) *Arabidopsis thaliana* plants, respectively, during heat shock stress and subsequent recovery. Acridine orange and DAPI staining was used to detect the accumulation of RNA/DNA molecules in different compartments in cells of leaves sampled from **A** untransformed WT plants or from of **D** *Ah2880* (line 12) or **G** *AhHAB4-PAI-1* (line 13) OE plants sampled in optimal conditions. Leaves of identical plant groups, i.e., **B** WT; **E** *Ah2880* OE and **H** *AhHAB4-PAI-1* OE, were also analyzed after an 11 h heat-shock treatment at 45 °C under constant illumination and after 1 day recovery under optimal conditions, i.e., **C** WT; **F** *Ah2880* OE and **I** *AhHAB4-PAI-1* OE. The use of arrows, dotted circles and brackets was intended to highlight important aspects of the respective images. Scale bars = 20 µm in (**D**) and (**G**); 10 µm in (**E**) and (**F**); 5 µm in (**A**), (**B**), (**H**) and (**I**) and 5 µm in (**C**)

Membrane integrity in leaf cells was evident in untreated WT plants (Fig. 6A, red fluorescence). In some stomatal cells an RNA/DNA fluorescence overlap was observed (Fig. 6A, yellow arrow). At HS-11, the cell membrane structure was disrupted and the nucleus decreased in size, where it was possible to appreciate fluorescence emitted by small RNA bodies (Fig. 6B, yellow arrow). At R-1, the presence of RNA was concentrated in the nuclei of stomatal cells, forming green spherical structures that were dissociated from DNA (Fig. 6C, dotted circles). Here, discontinuous membrane integrity was indicative of prominent cell membrane damage (Fig. 6C, yellow arrows). Sinuous cell membranes in *Ah2880* OE controls appeared unaltered. However, contrary to WT, nucleic-acid related fluorescent signals were practically absent (Fig. 6D). At HS-11, a slight increase in RNA fluorescence was detected, mostly in co-localization with DNA in the nuclei of dispersed cells (Fig. 6E, dotted circles). Cell membrane structure was also affected, as shown by the appearance of numerous interstitial spaces between cells, probably resulting from acute dehydration. After R-1, the size of the nuclei increased, concomitant with stronger RNA/DNA fluorescence signals. Some nuclei appeared to be saturated with RNA (Fig. 6F, yellow dotted circles), while the cell membranes were thicker and fused to endomembrane organelles (Fig. 6F, white arrow). The cytoplasm was thoroughly occupied by minute green dots that were representative of granular RNA accumulation (Fig. 6F, section enclosed within brackets). Nucleic acid-related fluorescent signals were conspicuously absent in *AhHAB4-PAI-1* OE untreated controls, and no membrane damage was observed (Fig. 6G). At HS-11, RNA-related fluorescent punctual signals, similar to those observed in *Ah2880* OE at R-1 were greatly abundant in practically all cellular compartments (Fig. 6H, section enclosed within brackets). At R-1, these signals were localized in nuclei, other organelles and in the vicinity of the membranes (Fig. 6I, yellow arrows).

### *A. thaliana* plants overexpressing the *AhHAB4-PAI-1* or *Ah2880* genes yield highly contrasting transcriptomic responses during HS and subsequent recovery

**Fig. 7:**
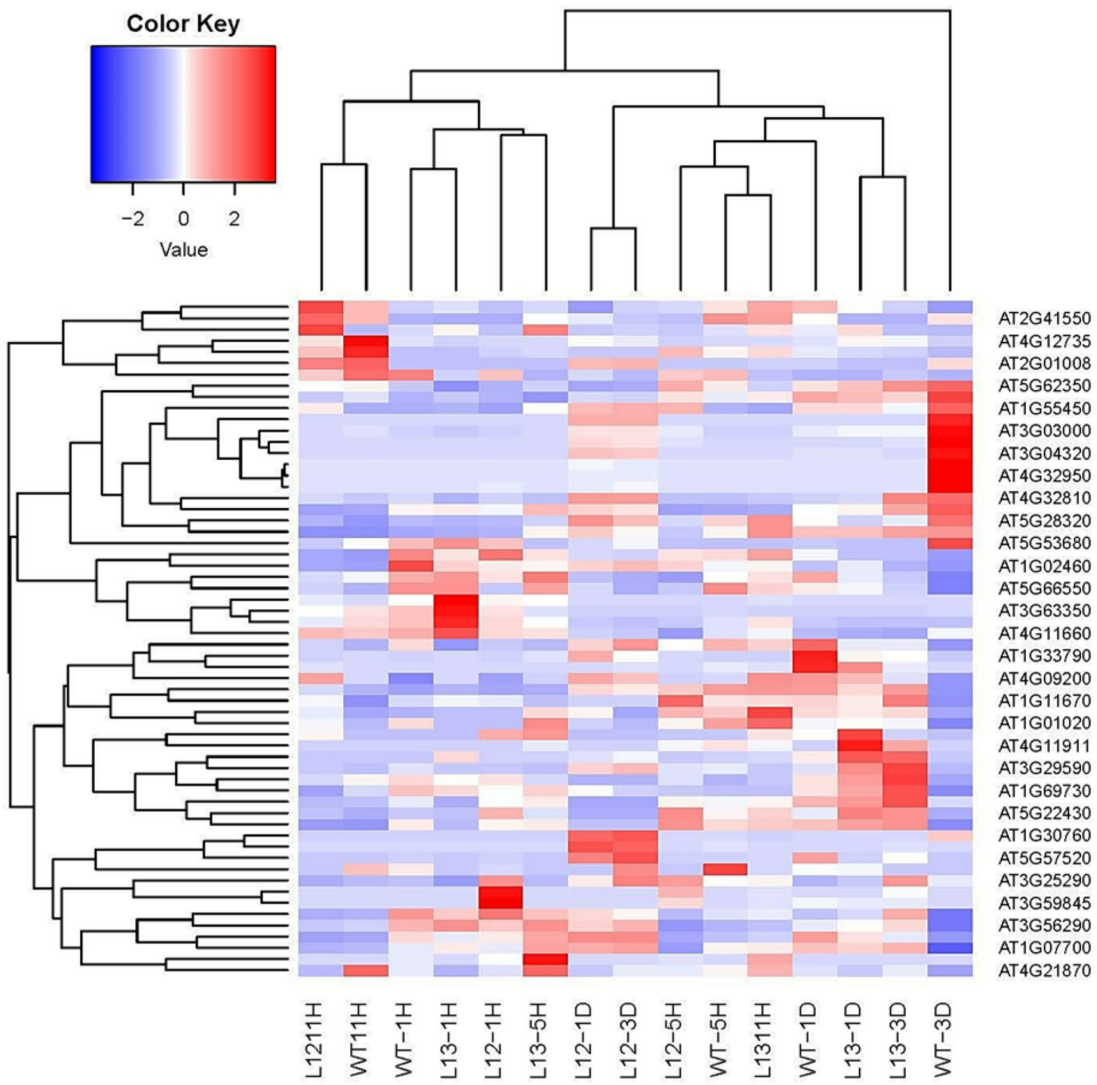
Heat map of differentially expressed genes in leaves of *AhHAB4-PAI-1* and *Ah2880* overexpressing (OE) *Arabidopsis thaliana* plants, respectively, during heat shock stress and subsequent recovery. The heat map shows the average standardized expression of the 60 genes selected for analysis (rows; refer to Table S6) in the 15 treatments (columns) included in this study. The expression *per* gene was standardized over treatments and the Color Key legend shows the intensity of the average expression *per* treatment. This heat map was generated by, firstly, estimating the average of FPKM expression of the 60 genes presented in Table S6 in the two biological replicates of each of the 15 treatments that conformed the analysis. Secondly, the matrix of average expression was standardized by rows (genes) to give a comparable measure of expression per gene. This produced a matrix of 60 rows (genes) and 15 columns (treatments) in which each row has a mean of zero and a standard deviation of one, allowing the visual comparison of expression per treatment. The function “heatmap.2” of the R package gplots (version 3.2.0) was applied to the standardized matrix to obtain this figure. The dendrograms presented in the margins of the figures were obtained from the Euclidean distances using the “hclust” function with the method “complete”

The strategy employed to analyze the transcriptomic data derived from the two transgenic *A. thaliana* plants was based on the identification of 13 water stress-associated TF groups (refer to Supplemental Experimental Procedures contained in the Supplemental Information file). This strategy was successful since it revealed patent differences in the type and temporality of the genes expressed during the HS and recovery phases in the *AhHAB4-PAI-1* and *Ah2880* OE plants. The differential gene expression patterns were in agreement with the result of the heat map generated with the standardized FPKM values estimated for 60 strongly modified genes, in the 15 treatments included in this study (Fig. 7; Table S6). The genes identified were divided into 8 relevant categories (CATs), as follows: CAT 1, group defining TFs and associated TFs; CAT 2, protein-related responses involving HSFs, HSPs and co-chaperones, protein post-translational modifications, transport, folding, oriented degradation and others; CAT 3, DNA repair, non-coding RNA activity, epigenetic regulation, DNA methylation, histone modifications, chromatin remodeling, epigenetic memory, RNA alternative splicing and others; CAT 4, cell wall/membrane modifications and ROS-associated responses; CAT 5 intercellular transport, carbon–nitrogen and secondary metabolism; CAT 6, ribosomal, chloroplast- and mitochondria-associated responses; CAT 7, growth, development, reproduction and phytohormone-related events, and CAT 8, molecular weight complex formation. The complete information derived from this transcriptomic analysis strategy can be found in Tables S3 to S5 in the Supplemental Information file. Here, the totality of the differentially induced transcripts detected using this strategy during early, i.e., 1 and 5 h (Table S3) and intermediate, i.e., 11 h (Table S4) HS phases and during the R1 and R3 recovery process, (Table S5), were grouped in one of the 8 categories mentioned above, together with their respective accession numbers and pertinent references. The rest of this section will be devoted to the most HS-relevant transcripts detected in each transgenic plant line during the HS and R phases, mentioned above.

#### Early response: HS-1 and HS-5

Here, an accumulation of differential transcripts, in practically every category, in *AhHAB4-PAI-1* OE plants, was observed. In contrast and except for a few categories, much diminished transcript types and numbers were detected in *Ah2880* OE plants. CAT 1 (group 8 TFs; Table S3) in *AhHAB4-PAI-1* OE plants was constituted by a few transcripts coding for bZIP62, MYB96 and NUCLEAR FACTOR Y, SUBUNIT A5 TFs, the latter of which promotes ABA-regulated wax synthesis to increase drought and oxidative stress tolerance. In *Ah2880* OE plants, several TFs (groups 6 and 10 TFs; Table S3) that mediate ABA-independent signaling cascades activated during heat stress, such as ETHYLENE-RESPONSE FACTOR and DREB TFs, in addition to the NGATHA-LIKE PROTEIN 3 TF that mediates heat stress responses through NINE-CIS-EPOXYCAROTENOID DIOXYGENASE 3, an ABA biosynthetic enzyme, were detected.

CAT 2 transcripts in *AhHAB4-PAI-1* OE plants code for proteins that are rapidly up-regulated in response to heat stress, like HSF7, HSFA2, HSP18.5, HSP60, HSP70T-2, HSP89.1, ATJ3 and DnaJ-domain chaperones and associated carboxylate-clamp (CC)-tetratricopeptide repeat (TPR) proteins. Also present were transcripts coding for the SOC3 protein involved in temperature-activated cell death. In CAT 3, salient transcripts code for heat-protective small nucleolar RNAs that cause pseudo-uridylation of RNA targets; SR45, a serine/arginine rich-like protein that regulates stress-related alternative splicing, and RAD50 ATPase, a DNA double-strand break repair enzyme. In *Ah28880* OE plants, the sole CAT 2 and CAT 3 HS-applicable transcripts code for the COP1-INTERACTING PROTEIN modulator of stress responses via its interaction with an E3 small ubiquitin-related modifier (SUMO) ligase, while the only relevant CAT 4 transcripts detected in *AhHAB4-PAI-1* OE plants code for galactinol synthase 2, an attenuator of stress-related ROS accumulation and lipid peroxidation. Conversely, CAT 4 transcripts in *Ah2880* OE plants that code for lignin and wax biosynthetic proteins predominated, including P bodies NAC SECONDARY WALL THICKENING PROMOTING FACTOR1, LTPG1, CER1 and others, such as miR775, known to reduce stress-induced ROS levels via an ethylene (ET)-ABA crosstalk, and DELTA (2)-ENOYL COA ISOMERASE 3, a heat stress-activated fatty acid degrader and modifier of membrane lipid composition.

Representative CAT 5 transcripts detected in *Ah2880* OE plants code for P-GLYCOPROTEIN 2, a heat-responsive ABC transporter, and for COPPER TRANSPORTER 1 and COPPER ION BINDING/ ELECTRON CARRIER PROTEIN, needed to provide the copper cofactor of several heat stress-activated enzymes and proteins. Salient CAT 5 transcripts in *AhHAB4-PAI-1* OE plants, code for a UREASE ACCESSORY PROTEIN, a stress activator of optimized N-use via urea recycling. Relevant CAT 6 transcripts in *Ah2880* OE plants code for a chloroplast HSP40 that mediates the farnesylation of DNA/RNA repair/modification heat stress-responsive proteins.

Both OE transgenic *A. thaliana* plants shared the accumulation of several transcripts, corresponding to CAT 7, coding for proteins involved in ABA-related signaling such as P-GLYCOPROTEIN 2, a heat-regulated transporter of ABA, and for the RESPONSIVE TO DEHYDRATION 22 and ABA 8’-HYDROXYLASE contributors to stress tolerance, respectively. Additional phytohormone-related transcripts that increased in *AhHAB4-PAI-1* OE plants code for the AtCKX7 cytokinin degrading enzyme recognized to enhance heat stress tolerance and for the SGT1b-like protein that participates in the stabilization of jasmonic acid (JA)-, auxin- and gibberellic acid (GA)-mediated receptors via complexation with HSP70-HSP90. CAT 8 transcripts coding for various subunits of the mitochondrial ATP synthase complex required for heat stress tolerance were relevant in *AhHAB4-PAI-1* OE plants.

#### Intermediate response: HS-11

The defining CAT 1 transcripts in *AhHAB4-PAI-1* OE plants (groups 3 and 5; Table S4) code for the MYB94 regulator of wax-biosynthesis; the CYTOKININ RESPONSE FACTOR 2 and TITANIA 2 abiotic stress modulators of cytokinin and auxin signaling; bHLH2/SAWTOOTH1, that controls both leaf morphology and heat stress tolerance, and EARLY FLOWERING 3, a temperature sensor and thermo-morphogenic factor. CAT 1 in *Ah2880* OE plants (group 7; Table S4) was characterized by transcripts coding for TF regulators of ABA-independent stress responses such as DREB AND EAR MOTIF PROTEIN 3, DREB2A and MYB1, and for an ABA-responsive HIT zinc finger TF, also present in the *Suaeda liaotungensis* halophyte.

Representative CAT 2 transcripts in *AhHAB4-PAI-1* OE plants were abundant. The most emblematic ones code for the Golgi apparatus complex-localized VPS54 HOMOLOG, known to increase thermotolerance and membrane integrity under heat stress; NEDD8/RUB ubiquitin-like protein, involved in the post-translational modification of proteins by neddylation; ATE1, an ARGINYL-tRNA: PROTEIN TRANSFERASE linked to the N-end rule/Arg/N-degron protein degradation pathway of TFs and HSPs during heat stress; EMBRYO DEFECTIVE 3012 that participates in the stress-induced and ER-localized degradation of glycoproteins, in addition to several members of the TPR-like proteins known to participate in protein folding and/or degradation. HS-associated CAT 2 transcripts in *Ah2280* OE plants were fewer and code for the PLANT U-BOX 48 E3 ligase, a regulator of heat responsive signaling pathways, and for an ER-ADENINE NUCLEOTIDE TRANSPORTER 1 that controls ER stress and the unfolded protein response in rice.

CAT 3 transcripts also differentially accumulated in *AhHAB4-PAI-1* and *Ah2880* OE plants. Those belonging to the former code for various HS-relevant genes, including DAMAGED DNA BINDING PROTEIN 1B, a regulator of UV damage-repair and heat stress responses; AtTHO5, involved in miRNA and siRNA biosynthesis; SPT5-like, ATRX-like and SNF2 helicase-like proteins that participate in diverse DNA methylation mechanisms and PCF11P-SIMILAR PROTEIN 4, involved in thermo-morphogenic events via alternative polyadenylation. Additional significant transcripts were found to code for microRNAs known to affect the expression of CC-NBS-LRR receptor and of the AP2/ERF and AUXIN RESPONSIVE TF genes in heat stressed tomato plants; HEN1, a heat stress-promoter of miRNA and siRNA uridylation; an RNA polymerase III/ RNA polymerase C subunit, that hastens the synthesis of stress-related non-coding RNAs; Forkhead-associated domain protein, able to mediate DNA repair and transcriptional regulation; tRNA/rRNA METHYLTRANSFERASE, a catalyzer of tRNAs post-transcriptional modifications, and the LIKE AMP1 ER-metalloprotease that regulates translational repression by miRNAs. In contrast, the weak representation of germane CAT 3 transcripts in *Ah22880* OE plants code for MEDIATOR 19B of RNA polymerase II, involved in heat stress memory and associated histone modifications; a glycine-rich RNA-binding protein, associated with several heat tolerance-related RNA processes, and N-Lysine methyltransferase, a facilitator of histone methylation during stress responses.

CAT 4 transcripts also readily accumulated in *AhHAB4-PAI-1* OE plants. Pertinent ones code for CELLULOSE SYNTHASE INTERACTIVE 2, a mediator of stress-activated cellulose synthase activity; KCS21, involved in the biosynthesis of very long chain fatty acids; CLASS III PEROXIDASE that promotes tissue lignification; TRICHOME BIREFRINGENCE-LIKE, implicated in the synthesis and deposition of secondary wall cellulose, and the AtGGH2 activator of the tetrahydrofolate antioxidant synthesis. The most significant CAT 4 transcripts detected in *Ah2280* OE plants code for the N-ACETYLGLUTAMATE KINASE promoter of arginine-related metabolite accumulation and antioxidant activity under heat stress.

CAT 6 transcripts were more abundant at the HS-11 stage in *Ah2880* OE plants. They code, mostly, for numerous ribosomal proteins and only for a few other heat-stress-related proteins. Those found among the latter included MALONYL-ACP that participates in thylakoid plastoglobuli formation that regulate lipid metabolism-related stress responses; chloroplast-localized CHAPERONIN 10, an enabler of protein folding during heat stress; plastidial THIOREDOXIN isoform, a provider of reducing power to a variety of stress-related enzymes, and PROHIBITIN 2, needed for mitochondrial dysfunction-activated retrograde stress signaling. CAT 6 transcripts detected in *AhHAB4-PAI-1 OE* plants code for SIGNAL PEPTIDE PEPTIDASE 1, part of a protease complex involved in chloroplast heat acclimation processes, and for a TPR PLASTID PROTEIN FACTOR, engaged in stress-induced RNA editing, in mitochondria and plastids.

Transcripts corresponding to CAT 7 in *Ah2880* OE plants were found to code mostly for Ca^2+^ signaling-related proteins, while those coding for bioactive peptide hormones abounded in *AhHAB4-PAI-1* OE plants. Among the former were EVOLUTIONARILY CONSERVED C-TERMINAL REGION 1, that acts via its interaction with stress-responsive CALCINEURIN B-LIKE-INTERACTING PROTEIN KINASE1, and a CHCH domain protein that mediates mechano-transduction of Ca^2+^, ROS and electrical signals generated during (a)biotic stresses. Transcripts coding for the CONSTANS-LIKE 3 regulator of plant thermo-morphogenic responses, were also relevant. Transcripts detected in the latter plants code for RGFR3, a receptor kinase that binds to the ROOT GROWTH FACTOR peptide hormones; BUDDHA’S PAPER SEAL 2 kinase that interacts with RALF4/19 peptide ligands and CLAVATA3/ ESR-related 23, a peptide signal involved in the regulation of the shoot apical meristem.

Compared to the presence of transcripts coding for CIS-CINNAMIC ACID RESPONSIVE PROTEIN, a latex-like protein involved in ABA-related stress responses, in *Ah2880* OE plants, its counterpart showed the accumulation of several ABA signaling-related transcripts coding for the PROTEIN PHOSPHATASE 2C and clade A PROTEIN PHOSPHATASE TYPE 2C negative ABA-regulators, and for the RPK1 receptor-like kinase, ABI 5 binding protein 3, ABA-INSENSITIVE PROTEIN KINASE 1 and ABA INSENSITIVE 1. Other indicative phytohormone-related transcripts detected in these plants code for the SNF1-related protein kinase 2 regulator of gibberellin, auxin, ET and cytokinin signaling; TAGK2 kinase, a controller of GA signaling acting through the inactivation of an E3 ubiquitin ligase; CML16, an ERF48-activated Ca^2+^ sensing protein that enhances stress tolerance, and the temperature-responsive AUXIN RESPONSE FACTOR 18.

Important in the context of their possible role in the generation of SGs, as proposed by the results shown previously in Fig. 6F and H, were a number of transcripts belonging to CAT 8 that accumulated in *AhHAB4-PAI-1* OE plants. These code for the STRESS RESPONSE SUPPRESSOR1 DExH box helicase; two RRM/RBD/RNP RNA binding proteins and a glycine-rich RNA binding protein, similar to other RNA-binding proteins that form part of SGs; an EXONUCLEASE 4-like protein detected in RNA processing bodies formed under heat stress, and various WD40 and ankyrin-repeat family proteins known to mediate protein–protein interactions.

#### Response during recovery: R-1 and R-3

Transcript accumulation was significantly increased in *AH2880* OE plants during recovery. CAT 1 transcripts (groups 1 and 2; Table S5) code for several different TFs belonging to the MYB (4), ERF/DREB/AP2 (12), WRKY and stress-responsive WRKY-interacting and VQ proteins (9), bHLH (8), and DOF/cDOF (3) families. Also represented were 9 NAC TFs, some of which enhance heat tolerance by inducing the expression of HSFs, HSPs and ROS scavenging protein genes; a bromodomain-containing TF, similar to others that maintain gene expression during heat stress by combining with RNA polymerase II kinases; the SCARECROW-LIKE PROTEIN 1 and 3 GRAS TFs involved in gibberellin-signaling; zinc-binding PLATZ TF, associated with membrane protection, antioxidant enzymes activation and up-regulated expression of development and cell proliferation genes via the activation of cyclin, gibberellin and other phytohormone biosynthetic genes; ALFIN1-like, SOD7 and DP-E2F-like 2 TFs, able to control plant development, partly via cell cycle control, and various other TFs known to regulate circadian rhythms, particularly PSEUDO-RESPONSE REGULATOR 7, a component of the temperature sensitive circadian system that regulates several stress-recovery responses and the stimulation of heat-induced phase separation. CAT 1 transcripts in *AhHAB4-PAI-1* OE plants (group 4; Table S5), were found to code predominantly for MYB TFs.

The number of CAT 2 transcripts also increased significantly during recovery in *Ah2880* OE plants. They code for several proteins associated with ER-related functions and autophagy. Among them were RING finger E3 ubiquitin ligase acting on a temperature-responsive ER membrane-bound auxin binding protein; PRT1 E3 ubiquitin ligase, a component of the N-end rule pathway; VACUOLAR SORTING RECPTOR 7, a ER-Golgi transport regulator and contributor to heat stress tolerance; VPS9a, a component of the autophagosome-based protein degradation system; LIPID DROPLET AND ER-ASSOCIATED PROTEIN, an organizer of autophagy receptors for ER turnover; UDP-GALACTOSE TRANSPORTER 6, part of the ER quality control mechanism to ensure proper glycoproteins folding; HEAT STRESS TRANSCRIPTION FACTOR A4A, that induces stress-associated gene transcription via MPK3/6 and MPK4 phosphorylation cascades; SMAX1- LIKE 6, a PHYTOCHROME-INTERACTING FACTOR 4-regulated chaperonin required for thermotolerance; AUTOPHAGY-RELATED 2 that targets heat-denatured HSPs and other proteins for degradation; the ATG8-INTERACTING PROTEIN 2 and CLATHRIN LIGHT CHAIN autophagy-related proteins that promote the assembly of the Golgi apparatus, in addition to the TPRKB-LIKE protein, an inducer of heat stress-activated autophagy in the *Pleurotus giganteus* mushroom.

CAT 3 to 6 transcripts were also much more abundant in *Ah2880* OE plants. Significant CAT 3 transcripts code for ESSENTIAL MEIOTIC ENDONUCLEASE 1B and MMZ3/UEV1C DNA repair proteins; class I POLY A-BINDING PROTEIN that regulates HS-responsive poly(A) tail length and HSP70 expression; CHROMATIN REMODELLING 12 and two SET domain containing proteins implicated in epigenetic and chromatin-based adaptations required for heat stress thermo-memory; METHYLTRANSFERASE G, a mediator of ribosomal RNA methylation and ribosome assembly during temperature stress; LUC7, a stress-responsive RNA alternative splicing regulator; miR163, a controller of heat stress-related gene expression, and the LOW protein, an RNA helicase that maintains rRNA homeostasis under high temperatures.

Relevant CAT 4 transcripts that accumulated in *Ah2880* OE plants code for numerous proteins that participate in enzymatic and non-enzymatic antioxidant stress-activated processes. Also detected were transcripts coding for the PDS1 plastoquinone and tocopherols biosynthetic enzyme; two TLD-domain containing nucleolar proteins that enhance photosynthesis efficiency via oxidative stress amelioration, and NUDIX HYDROLASE HOMOLOG 18, an adjuster of redox homeostasis and thermotolerance. Others associated with cell wall-related events code for several proteins involved in xyloglucan biosynthesis/ metabolism processes linked to the control of cell wall extensibility; an auxin- and stress-inducible EPOXIDE HYDROLASE involved in cutin monomer synthesis; WSD6, required for cuticular wax formation linked to enhanced thermotolerance; Golgi membrane-localized CELLULOSE SYNTHASE, a generator cell wall structural changes in response to heat stress in cotton; an ER-localized SPHINGOID LONG-CHAIN BASE-1-PHOSPHATE LYASE, involved in signaling pathways leading to programmed cell death, and several more transcripts coding for lignin biosynthetic enzymes. Additional transcripts code for FASCICLIN-LIKE ARABINOGALACTAN PROTEIN, a mediator of stress-related changes in secondary cell walls, and others coding for membrane-related proteins including MYOTUBULARIN 1, required for membrane-associated stress responses; the ALA-INTERACTING SUBUNIT 5 lipid transporter that modifies membranes as part of heat-adaptation lipid signaling events, and the SEC14P-LIKE phosphatidylinositol transfer protein that confers heat stress tolerance by altering membrane lipid composition. The much-reduced CAT 4 representation detected in *AhHAB4-PAI-1* OE plants included transcripts coding for the EXTENSIN PROLINE-RICH 1 cell wall modifier; MAPK 8, a stress-activator of Ca^2+^ release and ROS accumulation; GALACTOSE OXIDASE-LIKE enhancer of cell wall mechanical strength, and POLLEN OLE E1 proteins, involved in stress-responsive cell wall modifications.

Similarly abundant CAT 5 transcripts detected in *Ah2880* OE plants code for various heat stress-activated and ER-localized aquaporins; the NRT1.8, SLAH2, NIN Like Protein 7 and NLP7 modulators of nitrate transport, sensing and metabolism; PEPTIDE TRANSPORTER 1, CATIONIC AMINO ACID TRANSPORTER and ALANINE AMINOTRANSFERASE 2 that regulate N uptake, transport and utilization; the PLASMA MEMBRANE PROTON ATPase 2 cation channel that regulates Ca^2+^ influx during heat shock; three CYSTEINE-RICH RECEPTOR-LIKE PROTEIN KINASE modulators of UV radiation and heat stress responses via Ca^2+^ transport and ROS accumulation, and SULFATE TRANSPORTER 3, a mediator of the ET, H_2_S, and S cross-talk during heat stress responses. C and N metabolism-related CAT 5 transcripts code for the SUCROSE SYNTHASE 1, SUCROSE TRANSPORTER 4 and SUCROSE-PROTON SYMPORTER controllers of stress-regulated C allocation and growth during stress; the TREHALOSE-6-PHOSPHATE SYNTHASE 8 mediator of the trehalose 6-phosphate-related thermotolerance linked to C partitioning and sucrose homoeostasis; GLUCOKINASE, a controller of autophagy and/or programmed cell death; GLUTAMATE SYNTHETASE, positively correlated with heat tolerance in grasses;, and CHOLINE SYNTHASE, a glycine-betaine precursor that enhances antioxidant enzyme activity and PSII stability during heat stress. The much fewer CAT 5 related transcripts detected in *AhHAB4-PAI-1* OE plants were found to code mostly for N and C transporters, including the SWEET13 sucrose efflux transporter that mediates gibberellin uptake for plant development. Also found in *AhHAB4-PAI-1* OE plants were transcripts that code for PHENYL ALANINE AMMONIA-LYASE 3, involved in the stress-activated biosynthesis of protective secondary metabolites, and ANTHRANILATE SYNTHASE 2, linked to the protective accumulation of tryptophan and phenylalanine in heat stressed plants.

CAT 6 and CAT 7 were similarly represented in both transgenic plants. Germane CAT 6 transcripts in *Ah2880* OE plants code for CLPX, a CLP protease regulatory subunit that stabilizes HSP and ClpC1 chaperones in chloroplasts. In *AhHAB4-PAI-1* OE plants, the most apropos CAT 6 transcripts code for the COLD REGULATED 414 THYLAKOID MEMBRANE 1 protein that maintains chloroplast photosynthetic activity after stress-related damage, and the chloroplast-localized LON DOMAIN-CONTAINING PROTEIN 1 that controls H_2_O_2_ levels and lignin polymerization.

CAT 7 was abundantly represented in both transgenic plants in this phase. In the *Ah2880* OE plants, most of the relevant transcripts detected code for proteins involved in ABA biosynthesis, signaling and cross-talk with other phytohormones such as NDR1/HIN1-like 6, an activator of ABA signaling and biosynthesis; the HVA22 modulator of antioxidant responses; COPPER AMINE OXIDASE1, a mediator ABA- and polyamine-induced NO biosynthesis, and ABA-IMPORTING TRANSPORTER 1. Closely related transcripts code for DWARF 14, essential for strigolactone signaling, activation of temperature-stress responses and regulation of ABA and GA levels; CDL1, a regulator of brassinosteroids (BR) signaling and heat stress responses via a cross-talk with ABA, and two HSD1 11-β-hydroxysteroid dehydrogenases that mediate the BR-regulation of plant development. Others included CYP94B3, a jasmonoyl-isoleucine-12-hydroxylase attenuator of JA signaling and its involvement in plant thermo-tolerance; the GRETCHEN HAGEN 3.15 and ILR regulators of indole-3-acetic acid (IAA) and IAA conjugate levels, respectively, during heat stress; the SECRET AGENT O-GlcNAc transferase, RESPONSE REGULATOR 2 and MONOXYGENASE1 components of gibberellin, cytokinin and salicylic acid signaling, respectively and the ARGOS-LIKE TRANSMEMBRANE PROTEIN, ETHYLENE INSENSITIVE 1 and ETHYLENE INSENSITIVE 3 regulators of ET signaling during organ growth and/or thermotolerance. Other transcripts coding for heat stress-significant proteins included two NDR1/HIN1-like proteins, homologous to others induced in heat-stressed maize plants; PHYD, a red/far-red light photoreceptor that modulates the acclimation to extremely high temperatures; CPL, a circadian-regulated phosphatase induced in response to elevated temperatures; NIGHT LIGHT-INDUCIBLE AND CLOCK-REGULATED GENE 4, a circadian clock modulator of high-temperature tolerance; AtCP1, a Ca^2+^-binding protein that regulates growth and stress protection; PEROXIN11 that regulates peroxisomal H_2_O2 metabolism, among other functions, and the CASEIN KINASE I-LIKE 4 high-temperature TF-regulator.

In *AhHAB4-PAI-1* OE plants, detected CAT 7 transcripts mostly code for development-related proteins and peptide hormones. Among the most significative were the circadian clock-related SAP30 FUNCTION-RELATED 2 and SOUL heme-binding proteins. Others included DP-E2F-like 1, a preserver of the mitotic state of proliferating cells; JULGI1, an RNA-binding protein that enhances photosynthate allocation and plant growth; MEMBRANE-ASSOCIATED KINASE REGULATOR 3, which acts together with BRI1 KINASE INHIBITOR1 to influence phytohormones-mediated development; WALL-ASSOCIATED KINASE 2 that regulates cell growth and stress responses; LEUCINE-RICH REPEAT EXTENSIN 5, a controller of cell wall biogenesis and organization that interacts with RALF peptides; ROTUNDIFOLIA like 2 and ROTUNDIFOLIA like 16, small bioactive peptides that coordinate differentiation and growth; BES1/BZR1 homolog 1, BRASSINOSTEROID INSENSITIVE 1, BAK1-INTERACTING RECEPTOR-LIKE KINASE 3 and TCP DOMAIN PROTEIN 10 that regulate BR signaling during growth and stress responses; the GA-STIMULATED ARABIDOPSIS 6 peptide, antagonistically controlled by growth-promoting and stress-related hormones, respectively; ASPARTIC PROTEASE IN GUARD CELL 1, involved in ABA signaling and ROS degradation, and the SORTING NEXIN 2A regulator of ABA levels that operates together with the ATP BINDING CASETTE G25 ABA exporter.

CAT 8 transcripts were more prominently represented in *Ah2880* OE plants during the R phase. Interest was focused on transcripts coding for proteins possibly involved in SGs assembly, which are theorized to have formed in these plants during recovery, as shown in Fig. 6F. Two relevant transcript types were identified; one codes for an ankyrin-repeat family protein similar to others that mediate protein–protein interactions and are also known to be part of heat stress-induced protein-RNA complexes; the other, for ALBA1, which together with other ALBA proteins, phase separate into SGs and processing bodies under heat stress. Also detected were three WD40 repeat-like and PHOX4 CC-TPR proteins known to mediate protein–protein interactions, the latter by interacting with the Hsp90/Hsp70 co-chaperones.

## Discussion

This study revealed that the overexpression of two unknown function grain amaranth genes in *A. thaliana* plants significantly increased their capacity to recover from HS. The protective effect observed was in agreement with data suggesting their contribution to the high rates of survival observed in heat-shocked grain amaranths (Reyes-Rosales et al. 2023).

AhHAB4-PAI-1 is an RNA- and hyaluronan-binding protein. It is also similar to other proteins known to interact with CDC13, a single-strand telomeric DNA-binding protein and to associate with ribosomes and nuclear telomere cap complexes, a characteristic that supports its participation in several biological processes including abiotic stress responses (Van Dyke et al. 2004; Ambrosone et al. 2015). Likewise, the heat-responsiveness of *AhHAB4-PAI-1* in grain amaranth (Reyes-Rosales et al. 2023) as well as the protective effect against HS conferred to transgenic *A. thaliana* OE plants could be associated with the topology of its promoter region, which was found to be enriched in motifs previously identified in the regulatory region of several other heat stress-responsive plant genes. The presence of other distinctive motifs was also indicative of the often-found association between high light and heat stress (Zandalinas et al. 2020) (Table S2). Conversely, the abundance of DNA-binding One Zinc Finger (DOF) motifs in the promoter region of the *Ah2880* gene was congruent with findings reporting the heat-related induction of DOF TFs in wheat, Chinese cabbage and spinach (Table S2).

Likewise, the HS tolerance of *AhHAB4-PAI-1* OE plants was in agreement with evidence linking RNA-binding proteins with plant stress resistance, including extreme heat (Ambrosone et al. 2012, 2015; Nakaminami et al. 2012; Zhu et al. 2022). For instance, the glycine-rich D2 and D4 RNA-binding proteins have been found to be implicated in the heat stress-activated phase separation process that precedes the formation and subsequent accumulation of SGs that shield mRNA and proteins from damage during stressful conditions to permit subsequent adaptive and post-stress recovery cellular responses (Jain et al. 2016; Riback et al. 2017; Markmiller et al. 2018; Maruri-López et al. 2021; Zhu et al. 2022).Thus, SG formation is theorized to have contributed to the HS protection observed in *AhHAB4-PAI-1* OE plants. This argument is supported by the accumulation of fluorescent RNA-related granular signals observed in heat-shocked *AhHAB4-PAI-1* OE plants at HS-11, and less intensely, at R-1. In this sense, the transcriptomic data obtained at HS-11 and R-1 revealed the expression of a number of genes coding for possible SG protein components. It also supported the possibility that the enhanced HS tolerance shown by *AhHAB4-PAI-1* OE plants was further promoted by other properties attributed to RNA-binding proteins, such as AhHAB4-PAI-1. These entail their role in the activation of genes involved in the regulation of RNA metabolism, processing and transport (Muleya and Marondedze 2020) that was reflected by part of the transcriptomic data obtained at HS-11. Additional transcript accumulation patterns opened the possibility that complementary supporting mechanisms contributed to the increased HS tolerance observed in these plants, such as the rapid and sustained activation of the nuclear- and ER-centered HSFs and subsequent HSPs accumulation, together with the inducement of the unfolded protein response and several other protein-related responses associated with heat stress. Other likely scenarios incorporate epigenetic, miRNA, RNA splicing regulatory and/or protective mechanisms and diverse phytohormone signaling pathways, similar to others that have been found to be crucial for an efficient heat stress response in various plant models (Zhao et al. 2020; Doroodian and Hua 2021; Andrási et al. 2021; Lu et al. 2021; Singh et al. 2021).

The *Ah2880* grain amaranth gene is induced by various biotic and abiotic stress conditions (Délano-Frier et al. 2011; Cabrales-Orona and Délano-Frier 2021; Reyes-Rosales et al. 2023). This property was in agreement with the HS tolerance conferred to *Ah2880* OE plants. In this regard, it could be argued that the protective effect observed was somehow associated with the widespread expression of the GUS reporter gene during HS and with the distinct heat stress-dependent localization of GFP-Ah2880 proteins, mostly in membranes and nuclei of leaf cells, at HS-11, and in membranes and chloroplasts, at R-1. This time-course pattern was consistent with the temporality of *Ah2880’s* gene expression in heat-shocked grain amaranths (Reyes-Rosales et al. 2023). Correspondingly, the presence of a polyQ segment, embedded within the disordered region of Ah2880, which resembles those found in PrLDs, could have promoted thermotolerance through a mechanism similar to the one employed by EARLY FLOWERING 3 (ELF3) to regulate the circadian clock via light quality cues (Ronald et al. 2022). Furthermore, and relevant to the present study, was the association found between an integral polyQ repeat in ELF3 and its ability to act as a temperature sensor in *A. thaliana*, a role that involved phase separation and subsequent formation of liquid droplets, resembling SG formation (Jung et al. 2020; Hayes et al. 2021).

On the other hand, the Ah2880 protein secondary structure, predicted to consist of an N-terminal alpha-helix followed by an extended disordered region (Cabrales-Orona and Délano Frier 2021) was reminiscent of the structure of the OsDOF27 TF that was recently reported to confer thermotolerance in yeast and rice by still undefined mechanisms (Gandass et al. 2022). Moreover, the disordered C-terminal region of Ah2880 may have contributed to HS tolerance by protecting the integrity of membranes, similar to the *A. thaliana* VESICLE INDUCING PROTEIN IN PLASTID 1 (Zhang et al. 2016a), or by participating in SGs formation, which are rich in disordered proteins, and which appeared to accumulate in heat-shocked *Ah2880* OE plants at R-1. Additionally, transcriptomic data indicated that gene expression in these plants reached maximum levels during the HS-11 and R-1 phases. Transcript abundance at HS-11 was concentrated on three distinctive groups of genes involved in: i) cell wall/membrane re-modelling through secondary cell wall biogenesis, lignification, pectin modifications, cuticular wax deposition and other chemical modifications associated with changes in the plasticity, fluidity and/or rigidity of the cell wall/membrane structures known to enhance heat stress tolerance (Dos Santos et al. 2022; Xiang and Rathinasabapathi 2022); ii) reactive oxygen species-modulated heat stress acclimation responses acting together with ABA and other phytohormones to activate antioxidant and other heat-stress protective mechanisms (Devireddy et al. 2021), and iii) the accumulation of RPLs known to play an important role in stress-amelioration functions (Moin et al. 2016 and references therein). This property has been attributed to their participation as structural components of high-molecular weight complexes formed together with HSPs and/or as protein and RNA chaperones during heat stress conditions (Kang et al. 2016; Xiong et al. 2021). Some of the induced RPL genes also code for proteins required for proper chloroplast development and photosynthesis (Zhang et al. 2016b). Besides, transcript abundance detected in the R phase reflected a significant increase in the expression of genes encoding a great diversity of TFs, DNA repair and related mechanisms, cell wall modification and C and N metabolism proteins. This pattern suggested that the overexpression of *Ah2880* in *A. thaliana* plants provided an efficient heat stress-protective effect, by still to be defined mechanisms, that allowed them to resume vigorous growth under optimal post-stress conditions.

The present study generated data that proposed diverse mechanisms by means of which two unknown function grain amaranth proteins provided an increased tolerance to HS when overexpressed in *A. thaliana*. Their identification and validation will require further experimentation, which has the potential to uncover novel and meaningful strategies used by plants to withstand high temperature stress.

## Conclusions

This study revealed that the *AhHAB4-PAI-1* and *Ah2880* grain amaranth genes promote plant thermotolerance when overexpressed in *A. thaliana*. Microscopy analysis data suggested a link between the enhanced formation of RNA-enriched granular zones and the increased thermotolerance recorded in both transgenic plants. Moreover, a deep transcriptomic analysis provided evidence that the overexpression of *Ah2880* and *AhHAB4-PAI-1* activated widely different gene expression profiles in terms of the nature, abundance and temporality of the genes induced during the HS and R stages, a finding that supported the proposal that the heat stress-protective mechanisms observed in these plants were activated and executed by different mechanisms.

Finally, the heat stress-protective nature of the two unknown-function grain amaranth proteins tested was in accordance with their main distinctive characteristics, i.e., RNA-binding capacity and possession of an intrinsically disordered structure, respectively.

## Supplementary Information

Below is the link to the electronic supplementary material.

Supplementary Material 1

## Data Availability

All transcriptomic data (30 libraries as well as the text table containing the count matrix for each gene in each library) were deposited at the NCBI within “Gene Expression Omnibus” (GEO) with the number of accession “GSE277320”. The URL for the accession is: https://www.ncbi.nlm.nih.gov/geo/query/acc.cgi?&acc=GSE277320
