# Supplementary material for: Grain amaranth genes coding for an RNA-binding and a small, unknown function protein, respectively, enhance thermotolerance when overexpressed in Arabidopsis thaliana
Source: Physiol Mol Biol Plants. 2026 Jan 8;32(1):59–80. doi: 10.1007/s12298-025-01696-x (PMC12886626; doi:10.1007/s12298-025-01696-x)
Supplement: Supplementary file 1 — Supplementary Material 1 [file 12298_2025_1696_MOESM1_ESM.pdf]

Supplemental Figures:

(A) (B) (C)

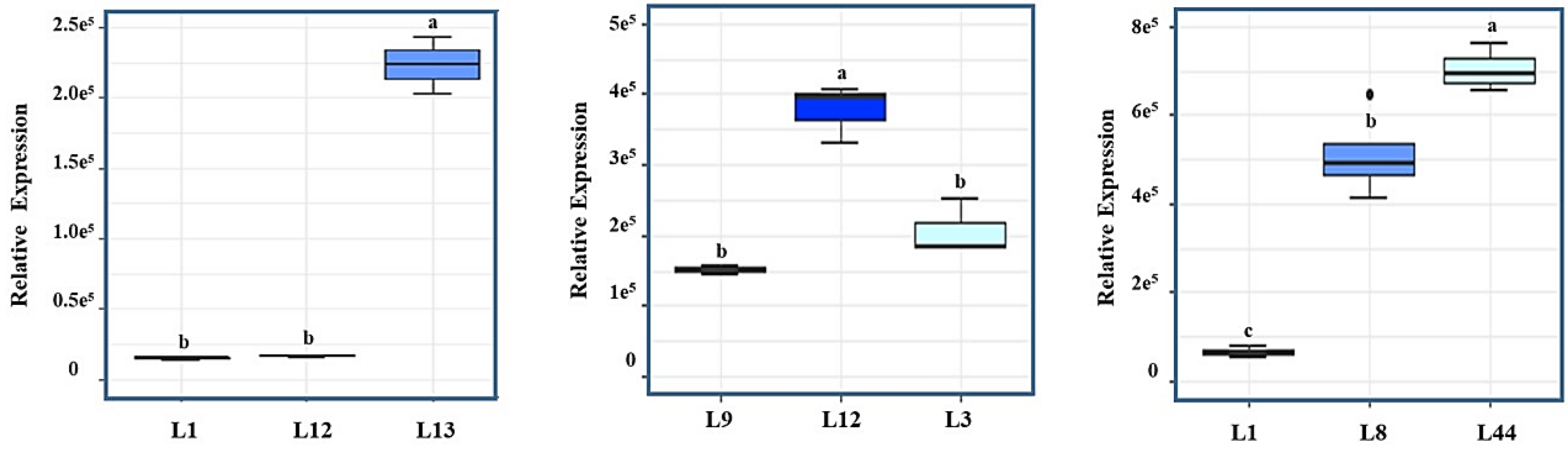

Figure S1 Expression levels of (A) *AhHAB4-PAI-1,* (B) *Ah2880* and (C) *Ah2880::GFP* in three independent lines of transgenic *Arabidopsis thaliana* plants. Relative expression levels shown were quantified based on the $2^{-\Delta\Delta C_T}$ calculation (Livak and Schmittgen 2001). C_T values for all genes were normalized to the C_T value of the *AtACT7* and *AtEF1a* housekeeping genes. Significantly different expression levels at $P \leq 0.05$ are indicated by different letters over the bars (One-way ANOVA, Tukey HSD test)

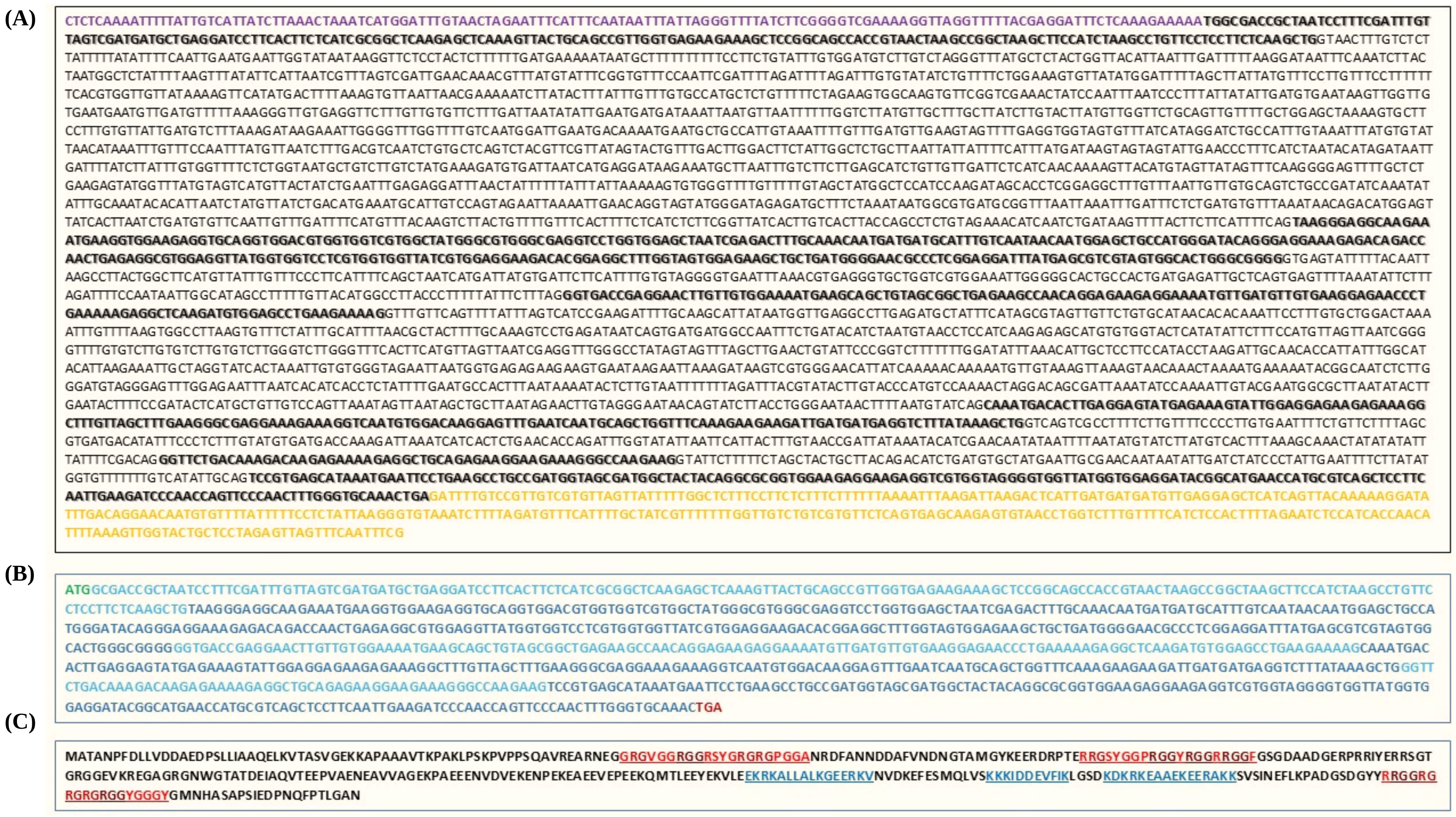

Figure S2 (**A**) The nucleotide sequence of the *AhHAB4-PAI-1* gene, containing six exons (shown in highlighted text). The 5'- and 3´-UTR are also shown in purple and orange text, respectively. (**B)** The coding sequence of the *AhHAB4-PAI-1* gene. The start and end codons are highlighted in green and red letters, respectively, whereas the assembled exons are shown in contrasting tones of blue. (**C**) Amino acid sequence of the AhHAB4-PAI-1 protein. The nucleic acid binding domains consisting of KR basic patches and RGG/RG motifs are shown in blue and red underlined text, respectively

(A)

CCATCAAACCGTAACCTTTCATCATCATTCATACCCATCTTCTCTCTTAGGACCAATAAAAAGAAAAAAGAAAAAAAGAAAAAAAAAAACAGGGGAAAAGGAAAGGGATAGATATTTGGATTGGAAGGATAGACTT
TGTAGGAAAGGGGTGGAAAAAACATGGCTAATGTATTGTAATTGTAATGTCCTCCAAACAAACAAAGCTCATTCATTATCATTCATTTTCTTTCTGAAAAAATATATTCCATAAATACCCTCCCTTGTTGCTTGATTTCT
GAGAGAATGGGTGATTGCCACACCCACAATGCCCCCCATTTCTATCCAAGTTCCACCTTCATCACAAGCCACCAACTTTCCTCTTCTCATATATTTTTTCTTGGGCCCTTTTTTGGGTTTTCACTTCCTCAACTTCTCTTCCA
TTCAATGTTTCCACAGAAAAAAAGAGAAAGTGAGAAACTTCAGCCTTCTTCTCTCTATATTATCACTTTTCACACCTTCTCTCTTTCTCTCTCTCTTATTATTATTCTTCTTCTTCTTTTCTCTGAAATCTTATCTCTTTTCTCT
GTTCTTCCTTTTTATTAGGGTTTTCTTCTTTTTTCTCTGTTTTCCTCCTGTTCTAGGGTTTGTGTTTTCGGTTGATTCTTGCAGATTTGGGGGAAATTGGTTGATGGGTATTTGATTCGATCGAAAGATTGTTAAGGGTTTT
C**ATGGATTCTCGCCGATCAACTTCTGTTGACGAGATTGCTAAAATTAGGGAAAAACATCAGTCTTTGTTGCAAGATTACTTGGTCTTGCAAAAGG**TTTGTCCTTTTTCTCTGTTTCTTTCTGTTATTGTTTTGATTTGATTG
ATTTTCTCTGGTTTTTCTTAGTTTTATAAGTTTTGGTTTGTTTGATTATGAGGCAATCGTGATCTGTTGTGGAATTGGTTTGTCTTTCTTTCTTAATTCTTGAATCTGACTTTTCGAGGTATTTGAATTTGTGTAACTTTTGAGTT
AATTATGATTATTCAACCTGTTTGGTTTAATTTGAATAAAATTCTGAAACTTGTTGAAGTTTGTGTTGATCAAAGGTAGGTGTTATACTTGATAATGTTTTGATTTCATTGGGATTATAATTGTGGTCAAAACACTGATTAAA
AGTATAAAAAATAATAAAATTAAGGAGATGAAAGGGCTTATTTGAAATTGGGATTTTTTATTAATCTTTGAGAAATTTAGATTAGTATTGATTATGTGTTGGACATGTGATTTAACTGCTTCACTTTGTTTGTAAAATTAGG
ATTGTGTATCAAAGAAGAGAAAGCTAAAGGAAACAAATGAAAAGAAAGAGACTCTTTTGGATGAAATTAGGTATGTGTGCTAAAACATTGATACTCATGAGCAAAGTTCTCATTTTATAAATTGTATACCGAAATCTAT
ATTGGTATACGATTGTTTACCAATTTTAGTCGGTTATTGTTTACAG**GTTTCTAAAGCGTAGACGAAATCTGTTGTCGAAGTTGAAATCACAAAAGCTTCAACCACAACAAGATACCATTCAGTTACAAAAAGCACCTCTT**
CAACATGAAGTTGGACAAGGTGGAAGTCGCGCAAGCACGAGTGAGCCACAATTGCAAACTCCATTGTTAGCAGTTGGTTCGATTTGGAATTCGGTAAGACATAAGCATTGGATAATGTCTTTGCAAACATAGAATTTG
AAGGTTGTTCTTGATTGATTTTCAATTGTGTTGCAG**GCTAGCCTCCCTAGAGAGGAAGTTGGTTTTCCGTCGGTGAAATTAGGGAAGAAGTCCAAGGATTTGTTTAGTAATGGCAAAAGAGTTGAGAAGAGGAAAATT**
TCTTGGCAAGATCAGTTGGCGTTGAAGGTTTAAGTGTTGAAACTTAGTTTTGTGCAGAAAACAGGAGTTGACTGATTCTTTCTTTTATCAATAGCTTATTTCTATAGAATACATAGAGTGTTAAATATGTGTCTTAGTA
GCAATGCCTTGGAACTTGTTGGATATGTGATGGATCAGTTTTATATGAAATTTTCAATGAATTGTTGTCAAATAATAAGAGGATTATTTTCAGTGTTTAGTGCTTGATGGTGTTTAGGATGGCATTTTATCAAACCAAT
ACCAGTAGATTTTTATTTTTTATTTGTGAAGTTTAAGATGATATTACCACCACAAGAGTCAATCGGAAAACTTGTTTGTTATCACAGACATGAATAAGATTTTCGTACATCCAACCCCTTCAAACCC

(B)

ATGGATTCTCGCCGATCAACTTCTGTTGACGAGATTGCTAAAATTAGGGAAAAACATCAGTCTTTGTTGCAAGATTACTTGGTCTTGCAAAAGGATTGTGTATCAAAGAAGAGAAAGCTAAAGGAAACAAATGAAA
AGAAAGAGACTCTTTTGGATGAAATTAGGTTTCTAAAGCGTAGACGAAATCTGTTGTCGAAGTTGAAATCACAAAAGCTTCAACCACAACAAGATACCATTCAGTTACAAAAAGCACCTCTTCAACATGAAGTTGGA
CAAGGTGGAAGTCGCGCAAGCACGAGTGAGCCACAATTGCAAACTCCATTGTTAGCAGTTGGTTCGATTTGGAATTCGGCTAGCCTCCCTAGAGAGGAAGTTGGTTTTCCGTCGGTGAAATTAGGGAAGAAGTCCA
AGGATTTGTTTAGTAATGGCAAAAGAGTTGAGAAGAGGAAAATTTCTTGGCAAGATCAGTTGGCGTTGAAGGTTTAA

(C)

MDSRRSTSVDEIAKIREKHQSLLQDYLVLQKDCVSKKRKLKETNEKKETLLDEIRFLKRRRNLLSKLKSQKLQPQQDTIQLQKAPLQHEVGQGGSRASTSEPQLQTPLLAVGSIWNSASLPREEVGFPSVKLG
KKSKDLFSNGKRVEKRKISWQDQLALKV

Figure S3 (**A**) The nucleotide sequence of the *Ah2880* gene; it contains four exons (shown in highlighted text) The 5’- and 3´-UTR are also shown in purple and orange text, respectively. (**B**) The coding sequence of the *Ah2880* gene. The start and end codons are highlighted in green and red letters, respectively whereas the assembled exons are shown in contrasting tones of blue. (**C**)Amino acid sequence of the Ah2880 protein. Glutamine (Q) residues hypothesized to be part a polyQ repeat involved in thermo-responsiveness are highlighted in red

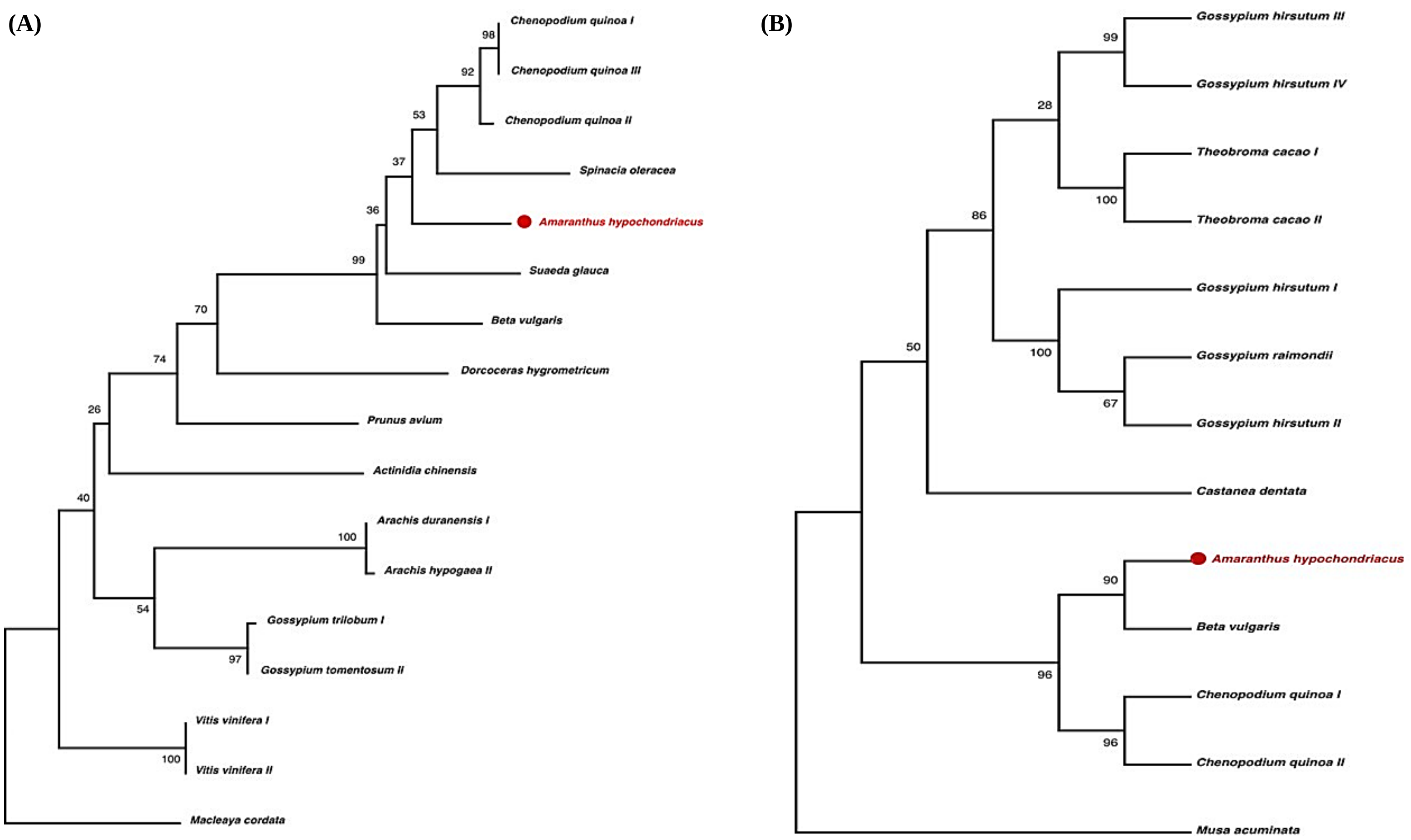

Figure S4 Phylogenetic analysis of the AhHAB4-PAI-1 and Ah2880 proteins. The phylogenies shown, obtained by the maximum likelihood method, show the relationship of the (**A**) AhHAB4-PAI-1 and (**B**) Ah2880 proteins**,** represented in the trees as *Amaranthus hypochondriacus*, in red text and marked with a red circle, with related proteins reported in closely related plant species (i.e., *Beta vulgaris*, *Chenopodium quinoa*, *Suaeda glauca* and *Spinacia oleracea*) and other dicot plants. The tree was built using MEGA software (version 11.0) using the maximum like-hood method based on the JTT matrix-based model (Jones et al. 1992). The bootstrap value next to the branches was estimated using the bootstrap test (1000 replicates)

Supplemental tables:

Table S1 Oligonucleotides utilized for the PCR amplifications required for the functional characterization of the *Ah2880* and *AhHAB4-PAI-1* genes in *A. thaliana*

**Experimental objective**	**Oligonucleotide sequence**	**Size (bp)**[a]
**Generation of** *Ah2880 and AhHAB4-PAI-1* **transgenic OE and** ***GFP::Ah2880 Arabidopsis thaliana*** **(*At*) plants**		
35S::*Ah2880* full length cDNA + UTR and N-*GFP-Ah2880* (F)	TCGATCGAAAGATTGTTAAGGGTTTTC	27
35S::*Ah2880* full length cDNA + UTR and N-*GFP-Ah2880* (R)	TTAAACCTTCAACGCCAACTGATCTT	26
35S::*AhHAB4-PAI-1* full length cDNA + UTR (F)	TCAATTCATCCTTAAACTAAATCC	24
35S::*AhHAB4-PAI-1* full length cDNA + UTR (R)	CACCCAAAGTTGCAAAGT	18
**Quantitative gene (qPCR) expression in transgenic *At***		
35S::*Ah2880* (F)	GCAGTTGGTTCGATTTGGA	19
35S::*Ah2880* (R)	AACCTTCAACGCCAACTGAT	20
35S::*AhHAB4-PAI-1* (F)	CTACACCTCTTCCACCTTCG	20
35S::*AhHAB4-PAI-1* (R)	TCAAGAGCTCAAAGTTACTGC	21
AtActin (F)	AATCACAGCACTTGCACC	18
AtActin (R)	ATTCCTGGACCTGCCTC	17
AtEF-1α (F)	TGCTGTTCTTATCATTGACTCC	22
AtEF-1α (R)	TTCATCGTACCTAGCCTTGG	20
**Generation of *GUS* reporter gene constructions in *At***		
pAh2880::GUS (F)	TGATCAAGAGTTCATGGCCGCTTTGTT	27
pAh2880::GUS (R)	AACAGAAGTTGATCGGCGAGAATCCAT	27
Green fluorescent protein **(transformation marker) in *At***		
eGFP (F)	CTGGTCGAGCTGGACGGCGA	20
eGFP (R)	CACGAACTCCAGCAGGACCA	20

[a]bp = base pairs

Table S2 Most abundant cis-regulatory elements present in the 1600 bp up-stream promoter region of the *AhHAB4-PAI-1* and *Ah2880* genes

Ah2880	
**Family**	**Function**
**HBP** (Recognized by homeobox proteins)	Regulation of genes involved in cell differentiation and development and reproductive processes involving meristem initiation and growth, expansion and organization of roots leaves and vascular tissues (xylem and phloem), vegetative to reproductive phase transition and the definition of bilateral symmetry.<br>Also present in nitric oxide-, auxin-, gibberellin- and abscisic acid-responsive genes controlling growth, development, and responses to blue light and environmental stress conditions (i.e., excess salt and water deprivation) and glucosinolate metabolism (Trindade et al. 1999; Mukherjee et al. 2009; Shi et al. 2011; Bolduc et al. 2012; Douglas et al. 2017; Meng et al. 2020; Zhao et al. 2021).
**GT-**Box (Identified in light-regulated promoters)	Abundant in genes that control plant growth regulation and responses to numerous stress conditions. Found in the defense-related chalcone synthase gene in in soya and common bean plants. Involved in shoot system and stomatal complex development; trichome morphogenesis; seed maturation and germination (Lawton et al. 1991**;** Terzaghi and Cashmore 1995; Majewska et al. 2022).
**L1- Box** (specific motifs for the expression of L1 proteins)	L1 proteins a known to control leaf, cotyledon and flower development and identity. It is relevant in the proline-rich Proteoderma factor 1 protein, exclusive of the L1 layer of the apical meristem, the protoderm and organ primordia cotyledon development; seed germination and dormancy (Abe et al. 2001; Lau et al. 2012; Rombolá-Caldentey et al. 2014).
**DOF** (DNA-binding One Zinc Finger)	The DOF family of TFs is involved in many plant-specific physiological processes including light responsiveness, seed maturation and germination, tissue differentiation, phytochrome and metabolic regulation in addition to resistance to drought stress (Noguero et al. 2013; Cheng et al. 2018; Sun et al. 2021). Regarding the latter, *Dof* genes were induced in Chinese cabbage (*Brassica rapa*) by several abiotic stresses, including heat and drought treatments (Ma et al. 2015); most *TaDof* genes were significantly upregulated by heat stress in wheat (Liu et al. 2020), whereas augmented expression of *SoDof22*, *SoDof3* and *SoDof15* was indicative of their possible role in the responses to heat, cold and drought stresses in spinach (*Spinace oleraceae*; Yu et al. 2021).
**MYB, MYBL (MYB** and **MYB-like** TF response elements)	See below.
AhHAB4-PAI-1	
**Plastid response element (PRE)**	Consensus sequence of PRE (*plastid response element*) in the regulatory regions of *HSP70A* promoters in *Chlamydomonas*; involved in induction of *HSP70A* gene by both Mg-protoporphyrin IX and light (van Gromoff et al. 2006). A forward genetic screen in *A. thaliana* supported the role of these factors in the retrograde signaling able to coordinate maximal thermotolerance, with highest HSP70 expression during the day, thereby linking chloroplast generated light signals with HSFA1 and heat shock gene expression (Dickinson et al. 2018).
**Initiator (Inr)** elements	"Inr (initiator)" elements found in the tobacco *psaDb* gene promoter without TATA boxes; Light- responsive transcription of *psaD*b depends on Inr, but not TATA box (Nakamura et al. 2002). Considered to play important regulatory roles in the expression of photosynthesis nuclear genes. Underlines the fact that the normal function of chloroplasts is dependent on the assembly and homeostasis of a large number of nucleus-encoded proteins (Ling et al. 2019).

**MYB CORE**	The MYB and MYB-like TF family comprises numerous proteins involved in the regulation of biotic and abiotic stress responses, as well as development, differentiation, metabolism and defense (Abdullah-Zawawi *et al.* 2021); *OsMYB55* improved tolerance to heat stress and drought in maize plants (El-Kereamy et al. 2012; Casaretto et al. 2016). Binding site for at least two Arabidopsis plant MYB proteins ATMYB1 and ATMYB2. ATMYB2 found to be involved in regulation of genes that are responsive to water stress. A petunia MYB protein (MYB.Ph3) is involved in regulation of flavonoid biosynthesis (Urao et al.1993; Solano et al. 1995). MYB30 was found to participate in oxidative and heat stress responses *via* the regulation of calcium signaling occurring through the repression of *ANN* (*ANNEXIN*) genes, which encode membrane Ca^{2+} transporter proteins that control cytosolic calcium concentrations and related calcium signatures (Liao et al. 2017).<br><br>The overexpression of the R2R3-OsMYB55 MYB protein in rice and maize was found to enhanced heat tolerance during vegetative growth (El-Kereamy et al. 2012; Casaretto et al. 2016). In cotton, binding of the GhMYB4 transcription factor protein to the promoter region (ProGhCKI) of a cotton casein kinase (*GhCKI*) gene in cotton was linked to the high temperature stress-related male sterility syndrome caused by defective anther development involving alterations in sugar and auxin signaling pathways and in DNA methylation (Min et al. 2014; Ma et al. 2018; Li et al. 2022).
**GCC CORE**	Core of GCC-box found in many pathogen-responsive genes such as *PDF1.2*, *Thi2.1*, and *PR4*. It has been shown to regulate jasmonate-responsive gene expression and to function as an ethylene- responsive element. (Brown et al. 2003; Chakravarthy et al. 2003).
**AGC BOX**	Enhancer activity, ethylene responsiveness, and binding of nuclear proteins depend on the integrity of two copies of the AGC box; AGCCGCC present in the promoters of several ethylene-responsive genes (e.g., Arabidopsis AtERF proteins that function as stress signal-response factors), including a large number of pathogenesis-related genes induced in response to pathogen attack (e.g., tobacco class I beta-1,3-glucanase gene) (Hart et al. 1993; Sato et al. 1996; Fujimoto et al. 2000; Ohme- Takagi et al. 2000; Rushton et al. 2002; Cheong et al. 2003; Zhang et al. 2004a).
**MYC CONSENSUS**	MYC recognition sites, also known as R response elements (RRE; Abe et al. 2003; Chinnusamy et al. 2003; Chinnusamy et al. 2004; Oh et al. 2005; Lee et al. 2005; Hartmann et al.2005; Agarwal et al. 2006) are found in the promoters of the dehydration-responsive gene *RD22* able to bind the ATMYC2 TF (previously known as rd22BP1). MYC recognition sequences are also found in the promoter regions of the *CBF3* (dehydration-responsive element binding factors 1 (DREB1s)/C- repeat-binding factor3) and ICE1 (inducer of CBF expression 1) genes that regulate the transcription of CBF/DREB1 genes in the cold in Arabidopsis ICE1 in tomato (*Solanum lycopersicum*) is a basic helix–loop–helix (bHLH) transcription factor encoding gene that shares similarity with *Arabidopsis* ICE1. The OE of *AtICE1* and *SlICE1* in *Arabidopsis* and tomato plants enhanced tolerance to freezing and provided chilling tolerance via the enhanced expression of cold-responsive genes as well as an accumulation of ascorbic acid., respectively (Chinnusamy et al. 2003; Lee et al. 2005; Miura et al. 2012a, 2012b).
**SURE CORE**	Core of sulfur-responsive element (SURE) found in the promoter of *SULTR1* coding for a high-affinity sulfate transporter gene that is upregulated in response to sulfur (S) deprivation in Arabidopsis. SURE also contains a auxin response factor (ARF) binding sequence (GAGACA) (Maruyama-Nakashita et al. 2004, 2005). Interestingly, of the 15 reported genes having the SURE element in their promoter (Maruyama-Nakashita et al. 2005), 13 genes were dependent on SULFUR LIMITATION1/ ETHYLENE-INSENSITIVE3-LIKE3 (EIL3) under S-deficiency (Dietzen et al. 2020) (see below), suggesting that SLIM1/EIL3 might also be recognized by the SURE element. This is a further indication of the dual participation of ethylene and hydrogen sulfide (H2S) in signaling pathways designed to trigger defense responses against heat stress through several mechanisms, some of which involve S-containing compounds such as glutathione, cysteine, reactive sulfur species and others (Sehar et al. 2002; Ristova et al. 2022).

**WRKY71**	A core of TGAC-containing W-box is present in the Amy32b gene promoter. Also, recognized as the binding site of rice WRKY71, which acts as a transcriptional repressor of the gibberellin signaling pathway and of parsley WRKY regulatory proteins that bind specifically to TGAC-containing W box elements within the Pathogenesis-Related Class10 (PR-10) genes (Zhang et al. 2004b; Xie et al. 2005; Eulgem et al. 1999; Eulgem et al. 2000). The rice *Os*WRKY71 was further proposed to play a significant role in plant abiotic stress resistance, including heat stress (Deeba et al. 2020), while an up-regulated expression of Musa*WRKY71* expression in banana plantlets was produced in response to cold, dehydration, salt, ABA, H_2O_2, ethylene, salicylic acid and methyl jasmonate (Shekhawat et al. 2011). Other WRKY TF members, e. g. AtWRKY30 and TaWRKY33, significantly increased heat tolerance when over expressed in wheat and Arabidopsis plants, respectively (El-Esawi et al. 2019).
**ABRE (LATE ERD1); ACGTAT (ERD1)**	ABRE-like sequence (from -199 to -195) required for etiolation-induced expression of *ERD1 (EARLY RESPONSIVE TO DEHYDRATION*) in Arabidopsis (Simpson et al. 2003; Nakashima et al. 2006). ACGT sequence (from -155 to -152) required for etiolation-induced expression of ERD1 (*EARLY RESPONSIVE TO DEHYDRATION*) in Arabidopsis (Simpson et al. 2003). AtERD1is known to induce the expression of *AtERD2* and *AtERD8* coding for two heat shock proteins: HSP70T-1 and HSP81.2 (HSP90.1) (Wu et al. 2022).
**GT-1, GmSCAM4**	"GT-1 motif" found in the promoter of the *SCaM-4* gene coding for a pathogen- and salt-induced the CaM isoform in soybean (*Glycine max*) (Park et al. 2004). GT-1 encodes a plant transcription factor that binds to one of the cis-acting elements, BoxII, which resides within the upstream promoter region of light-responsive genes. GT-1 presumably acts as a molecular switch modulated through Ca^{2+}-dependent phosphorylation/dephosphorylation in response to light signals. GT-1 and GT-2 are nuclear factors that interact with light-responsive gene promoters and are homologous within their functionally defined DNA-binding domains. GT-2 differs structurally from GT-1 in that it has twin DNA- binding domains whereas GT-1 possesses only one trihelix DNA-binding motif (Gilmartin et al. 1992; Smalle et al. 1998). Recently, GT-1 was identified as a key mediator linking nitric oxide-dependent signal perception to the activation of cellular heat responses (He et al. 2022).
**SORLIPs**	One of five "Sequences Over-Represented in Light-Induced Promoters" computationally identified in *Arabidopsis thaliana* as phyA-induced motifs (from SORLIP1 to SORLIP5) (Hudson et al. 2003). Required for high light (HL) induction of genes coding for "Early Light Induced Proteins" (ELIPs) belonging to the light harvesting complex (LHC) superfamily of proteins, some of which are thought to act as a photo-protectants against the damaging effects of HL (Rus Alvarez-Canterbury et al. 2014). Similar motifs were found to regulate ultraviolet B radiation and cold stress responses in *A. thaliana* (Hayami et al. 2015). The duplication and triplication of a SORLIP1 motif present in the promoter of the light-inducible protein gene (LIP) of *Dunaliella salina* dramatically increased its high light response (Baek et al. 2016). SORLIP motifs might be responsible for the observed light- expression pattern of phototropins in *Brachypodium distachyon* (Krzeszowiec et al. 2020).

Abdullah-Zawawi M.R., Ahmad-Nizammuddin N.F., Govender N., Harun S., Mohd-Assaad N., Mohamed-Hussein Z.A., 2021. Comparative genome-wide analysis of WRKY, MADS-box and MYB transcription factor families in *Arabidopsis* and rice. Sci. Rep. 11, 19678. doi: 10.1038/s41598-021-99206-y

Abe M., Takahashi T., Komeda Y., 2001. Identification of a cis-regulatory element for L1 layer-specific gene expression, which is targeted by an L1-specific homeodomain protein. Plant J. 26, 487-94. doi: 10.1046/j.1365-313x.2001.01047.x

Abe H., Urao T., Ito T., Seki M., Shinozaki K., Yamaguchi-Shinozaki, K., 2003. Arabidopsis AtMYC2 (bHLH) and AtMYB2 (MYB) function as transcriptional activators in abscisic acid signaling. Plant Cell 15, 63-78. doi: 10.1105/tpc.006130

Agarwal M., Hao Y., Kapoor A., Dong C.H., Fujii H., Zheng X., Zhu J.K., 2006. A R2R3 type MYB transcription factor is involved in the cold regulation of CBF genes and in acquired freezing tolerance. J. Biol. Chem. 281, 37636-37645. doi: 10.1074/jbc.M605895200

Baek K., Lee Y., Nam O., Park S., Sim S.J., Jin E., 2016. Introducing *Dunaliella LIP* promoter containing light-inducible motifs improves transgenic expression in *Chlamydomonas reinhardtii*. Biotechnol. J. 11, 384-392. doi: 10.1002/biot.201500269.

Bolduc N., Yilmaz A., Mejia-Guerra M.K., Morohashi K., O'Connor D., Grotewold E., Hake S., 2012. Unraveling the KNOTTED1 regulatory network in maize meristems. Genes Dev. 26, 1685-1690. http://www.genesdev.org/cgi/doi/10.1101/gad.193433.112.

Brown R.L., Kazan K., McGrath K.C., Maclean D.J., Manners J.M., 2003. A role for the GCC-box in jasmonate-mediated activation of the *PDF1.2* gene of Arabidopsis. Plant Physiol. 132, 1020- 1032. doi: 10.1104/pp.102.017814

Casaretto J.A., El-Kereamy A., Zeng B., Stiegelmeyer S.M., Chen X., Bi Y.M., Rothstein S.J., 2016. Expression of *OsMYB55* in maize activates stress-responsive genes and enhances heat and drought tolerance. BMC Genomics 2016 17, 312. doi: 10.1186/s12864-016-2659-5

Chakravarthy S., Tuori R.P., D'Ascenzo M.D., Fobert P.R., Despres C., Martin G.B., 2003. The tomato transcription factor Pti4 regulates defense-related gene expression via GCC box and non- GCC box cis elements. Plant Cell 15, 3033-3050. doi: 10.1105/tpc.01757

Cheng Z., Hou D., Liu J., Li X., Xie L., Ma Y., Gao J., 2018. Characterization of moso bamboo (*Phyllostachys edulis*) Dof transcription factors in floral development and abiotic stress responses. Genome 61, 151-156. doi: 10.1139/gen-2017-0189

Cheong Y.H., Moon B.C., Kim J.K., Kim C.Y., Kim M.C., Kim I.H., Park C.Y., Kim J.C., Park B.O., Koo S.C., Yoon H.W., Chung W.S., Lim C.O., Lee S.Y., Cho J., 2003. BWMK1, a rice mitogen- activated protein kinase, locates in the nucleus and mediates pathogenesis-related gene expression by activation of a transcription factor. Plant Physiol. 132, 1961-1972. doi: 10.1104/pp.103.023176 Chinnusamy V., Ohta M., Kanrar S., Lee B.H., Hong X., Agarwal, M. Zhu J.K., 2003. ICE1: a regulator of cold-induced transcriptome and freezing tolerance in Arabidopsis. Genes Dev. 17, 1043- 1054. doi: 10.1101/gad.1077503

Chinnusamy V., Schumaker K., Zhu, J.K., 2004. Molecular genetic perspectives on cross-talk and specificity in abiotic stress signalling in plants. J. Exp. Bot. 55, 225-236. doi: 10.1093/jxb/erh005 Deeba F., Sultana T., Majeed N., Naqvi S., 2020. Heterologous expression of a plant WRKY protein confers multiple stress tolerance in *E. coli*. Turkish J. Biochem. 45, 483. doi: 10.1515/tjb-2018- 0483

Dickinson P.J., Kumar M., Martinho C., Yoo S.J., Lan H., Artavanis G., Charoensawan V., Schöttler M.A., Bock R., Jaeger K.E., Wigge P.A., 2018. Chloroplast signaling gates thermotolerance in Arabidopsis. Cell Rep. 22, 1657-1665. doi: 10.1016/j.celrep.2018.01.054

Dietzen C., Koprivova A., Whitcomb S.J., Langen G., Jobe T.O., Hoefgen R., Kopriva S., 2020. The transcription factor EIL1 participates in the regulation of sulfur-deficiency response. Plant Physiol. 184, 2120-2136. https://doi.org/10.1104/pp.20.01192

Douglas S.J., Li B., Kliebenstein D.J., Nambara E., Riggs C.D., 2017. A novel *Filamentous Flower* mutant suppresses brevipedicellus developmental defects and modulates glucosinolate and auxin levels. PLoS ONE 12, e0177045. doi: 10.1371/journal.pone.0177045.

El-Esawi M.A., Al-Ghamdi A.A., Ali H.M., Ahmad M., 2019. Overexpression of *AtWRKY30* transcription factor enhances heat and drought stress tolerance in wheat (*Triticum aestivum* L.). Genes (Basel) 10, 163. doi: 10.3390/genes10020163

El-Kereamy A., Bi Y.M., Ranathunge K., Beatty P.H., Good A.G., Rothstein S.J., 2012. The rice R2R3-MYB transcription factor OsMYB55 is involved in the tolerance to high temperature and modulates amino acid metabolism. PLoS ONE 7, e52030. doi: 10.1371/journal.pone.0052030

Eulgem T., Rushton P.J., Robatzek S., Somssich I.E., 2000. The WRKY superfamily of plant transcription factors. Trends Plant Sci. 5, 199-206. doi: 10.1016/s1360-1385(00)01600-9

Eulgem T., Rushton P.J., Schmelzer E., Hahlbrock K., Somssich I.E., 1999. Early nuclear events in plant defence signalling: rapid gene activation by WRKY transcription factors. EMBO J. 18, 4689- 4499. doi: 10.1093/emboj/18.17.4689

Fujimoto S.Y., Ohta M., Usui A., Shinshi H., Ohme-Takagi M., 2000. Arabidopsis ethylene-responsive element binding factors act as transcriptional activators or repressors of GCC box-mediated gene expression. Plant Cell 12, 393-404. doi: 10.1105/tpc.12.3.393

Gilmartin P.M., Memelink J., Hiratsuka K., Kay S.A., Chua N.H., 1992. Characterization of a gene encoding a DNA binding protein with specificity for a light-responsive element. Plant Cell 4, 839- 849. doi: 10.1105/tpc.4.7.839

Hart C.M., Nagy F., Meins F. Jr., 1993. A 61 bp enhancer element of the tobacco beta-1,3-glucanase B gene interacts with one or more regulated nuclear proteins. Plant Mol. Biol. 21, 121-131. doi: 10.1007/BF00039623

Hartmann U., Sagasser M., Mehrtens F., Stracke R., Weisshaar B., 2005. Differential combinatorial interactions of cis-acting elements recognized by R2R3-MYB, BZIP, and BHLH factors control light-responsive and tissue-specific activation of phenylpropanoid biosynthesis genes. Plant Mol. Biol. 57, 155-171. doi: 10.1007/s11103-004-6910-0

Hayami N., Sakai Y., Kimura M., Saito T., Tokizawa M., Iuchi S., Kurihara Y., Matsui M., Nomoto M., Tada Y., Yamamoto Y.Y., 2015. The responses of Arabidopsis *Early Light-Induced Protein2* to ultraviolet B, high light, and cold stress are regulated by a transcriptional regulatory unit composed of two elements. Plant. Physiol. 169, 840-855. https://doi.org/10.1104/pp.15.00398

He N.Y., Chen L.S., Sun A.Z., Zhao Y., Yin S.N., Guo F.Q., 2022. A nitric oxide burst at the shoot apex triggers a heat-responsive pathway in Arabidopsis. Nat. Plants. 8, 434-450. doi: 10.1038/s41477- 022-01135-9.

Hudson M.E., Quail, P.H., 2003. Identification of promoter motifs involved in the network of phytochrome A-regulated gene expression by combined analysis of genomic sequence and microarray data. Plant Physiol. 133, 1605-1616. doi: 10.1104/pp.103.030437.

Krzeszowiec W., Novokreshchenova M., Gabryś, H., 2020. Chloroplasts in C3 grasses move in response to blue-light. Plant Cell Rep. 39, 1331-1343. doi: 10.1007/s00299-020-02567-3

Lau S., Slane D., Herud O., Kong J., Jürgens G., 2012. Early embryogenesis in flowering plants: Setting up the basic body pattern. Annu. Rev. Plant Biol. 63, 483-506. https://doi.org/10.1146/annurev- arplant-042811-105507

Lawton M.A., Dean S.M., Dron M., Kooter J.M., Kragh K.M., Harrison M.J.,

Yu L., Tanguay L., Dixon R.A., Lamb C.J., 1991. Silencer region of a chalcone synthase promoter contains multiple binding sites for a factor, SBF-1, closely related to GT-1. Plant Mol. Biol. 16, 235-249. doi: 10.1007/BF00020555

Li Y., Li Y., Su Q., Wu Y., Zhang R., Li Y., Ma Y., Ma H., Guo X., Zhu L., Min L., Zhang X., 2022. High temperature induces male sterility via MYB66-MYB4-Casein kinase I signaling in cotton. Plant Physiol. 189, 2091-2109. doi: 10.1093/plphys/kiac213

Liao C.C., Zheng Y., Guo Y., 2017. MYB30 transcription factor regulates oxidative and heat stress responses through ANNEXIN-mediated cytosolic calcium signaling in Arabidopsis. New Phytol. 216, 163-177. doi: 10.1111/nph.14679

Lee B.H., Henderson D.A., Zhu, J.K., 2005. The Arabidopsis cold-responsive transcriptome and its regulation by ICE1. Plant Cell 17, 3155-3375. doi: 10.1105/tpc.105.035568

Ling Q., Broad W., Trösch R., Töpel M., Demiral Sert T., Lymperopoulos P., Baldwin A., Jarvis R.P., 2019. Ubiquitin-dependent chloroplast-associated protein degradation in plants. Science 363, eaav4467. doi: 10.1126/science.aav4467

Liu Y., Liu N., Deng X., Liu D., Li M., Cui D., Hu Y., Yan Y., 2020. Genome-wide analysis of wheat DNA-binding with one finger (Dof) transcription factor genes: evolutionary characteristics and diverse abiotic stress responses. BMC Genomics 21, 276. doi: 10.1186/s12864-020-6691-0

Ma J., Li M.Y., Wang F., Tang J., Xiong A.S., 2015. Genome-wide analysis of Dof family transcription factors and their responses to abiotic stresses in Chinese cabbage. BMC Genomics 16, 33. https://doi.org/10.1186/s12864-015-1242-9

Majewska M., Kuźma Ł., Szymczyk P., 2022. Isolation and comprehensive in silico characterisation of a new 3-hydroxy-3-methylglutaryl-coenzyme a reductase 4 (HMGR4) gene promoter from *Salvia miltiorrhiza*: comparative analyses of plant *HMGR* promoters. Plants (Basel), 11, 1861. doi: 10.3390/plants11141861

Maruyama-Nakashita A., Nakamura Y., Watanabe-Takahashi A., Inoue E., Yamaya T., Takahashi H., 2005. Identification of a novel cis-acting element conferring sulfur deficiency response in Arabidopsis roots. Plant J. 42, 305-314. doi: 10.1111/j.1365-313X.2005.02363.x

Maruyama-Nakashita A., Nakamura Y., Watanabe-Takahashi A., Yamaya T., Takahashi H., 2004. Induction of SULTR1;1 sulfate transporter in Arabidopsis roots involves protein phosphorylation/dephosphorylation circuit for transcriptional regulation. Plant Cell Physiol. 45, 340-345. https://doi.org/10.1093/pcp/pch029.

Meng L., Liu X., He C., Xu B., Li Y., Hu Y., 2020. Functional divergence and adaptive selection of *KNOX* gene family in plants. Open Life Sci. 15, 346-363. doi: 10.1515/biol-2020-0036

Min L., Li Y., Hu Q., Zhu L., Gao W., Wu Y., Ding Y., Liu S., Yang X., Zhang X., 2014. Sugar and auxin signaling pathways respond to high-temperature stress during anther development as revealed by transcript profiling analysis in cotton. Plant Physiol. 164, 1293-308. doi: 10.1104/pp.113.232314

Miura K., Sato A., Shiba H., Kang S.W., Kamada H., Ezura H., 2012b. Accumulation of antioxidants and antioxidant activity in tomato, *Solanum lycopersicum*, are enhanced by the transcription factor *SlICE1*. Plant Biotechnol. 29, 261-269. http://dx.doi.org/10.5511/plantbiotechnology.12.0303b

Miura K., Shiba H., Ohta M., Kang S.W., Sato A., Yuasa T., Iwaya-Inoue M., Kamada H. Ezura H., 2012a. SlICE1 encoding a MYC-type transcription factor controls cold tolerance in tomato, *Solanum lycopersicum*. Plant

Biotechnol. 29, 253-260. doi: 10.5511/plantbiotechnology.12.0303a
Mukherjee K., Brocchieri L., Bürglin T.R., 2009. A comprehensive classification and evolutionary analysis of plant homeobox genes. Mol. Biol. Evol. 26, 2775-2794. doi: 10.1093/molbev/msp201 Nakamura M., Tsunoda T., Obokata, J., 2002. Photosynthesis nuclear genes generally lack TATA-boxes: a tobacco photosystem I gene responds to light through an initiator. Plant J. 29, 1-10. doi: 10.1046/j.0960-7412.2001.01188.x
Nakashima K., Fujita Y., Katsura K., Maruyama K., Narusaka Y., Seki M., Shinozaki K., Yamaguchi-Shinozaki K., 2006. Transcriptional regulation of ABI3- and ABA-responsive genes including RD29B and RD29A in seeds, germinating embryos, and seedlings of Arabidopsis. Plant Mol. Biol. 60, 51-68. doi: 10.1007/s11103-005-2418-5
Noguero M., Atif R.M., Ochatt S., Thompson R.D., 2013. The role of the DNA-binding One Zinc Finger (DOF) transcription factor family in plants. Plant Sci. 209, 32-45. doi: 10.1016/j.plantsci.2013.03.016
Oh S.J., Song S.I., Kim Y.S., Jang H.J., Kim S.Y., Kim M., Kim Y.K., Nahm B.H., Kim J.K., 2005. Arabidopsis CBF3/DREB1A and ABF3 in transgenic rice increased tolerance to abiotic stress without stunting growth. Plant Physiol. 138, 341-351. doi: 10.1104/pp.104.059147
Ohme-Takagi M., Suzuki K., Shinshi H., 2000. Regulation of ethylene-induced transcription of defense genes. Plant Cell Physiol. 41, 1187-1192. https://doi.org/10.1093/pcp/pcd057 Park H.C., Kim M.L., Kang Y.H., Jeon J.M., Yoo J.H., Kim M.C.,
Park C.Y., Jeong J.C., Moon B.C., Lee J.H., Yoon H.W., Lee S.H., Chung W.S., Lim C.O., Lee S.Y., Hong J.C., Cho M.J., 2004. Pathogen- and NaCl-induced expression of the SCaM-4 promoter is mediated in part by a GT-1 box that interacts with a GT-1-like transcription factor. Plant Physiol. 135, 2150-2161. doi: 10.1104/pp.104.041442
Ristova D., Kopriva S., 2022. Sulfur signaling and starvation response in Arabidopsis. iScience 25, 104242. doi: 10.1016/j.isci.2022.104242
Rombolá-Caldentey B., Rueda-Romero P., Iglesias-Fernández R., Carbonero P., Oñate-Sánchez L., 2014. Arabidopsis DELLA and two HD-ZIP transcription factors regulate GA signaling in the epidermis through the L1 box cis-element. Plant Cell 26, 2905-2919. doi: 10.1105/tpc.114.127647
Rus Alvarez-Canterbury A.M., Flores D.J., Keymanesh K., To K., Brusslan J.A., 2014. A double SORLIP1 element is required for high light induction of ELIP genes in *Arabidopsis thaliana*. Plant Mol. Biol. 84, 259-267. https://doi.org/10.1007/s11103-013-0130-4
Rushton P.J., Reinstädler A., Lipka V., Lippok B., Somssich I.E., 2002. Synthetic plant promoters containing defined regulatory elements provide novel insights into pathogen- and wound-induced signaling. Plant Cell 14, 749-762. doi: 10.1105/tpc.010412
Sato F., Kitajima S., Koyama T., Yamada, Y., 1996. Ethylene-induced gene expression of osmotin-like protein, a neutral isoform of tobacco PR-5, is mediated by the AGCCGCC cis-sequence. Plant Cell Physiol. 37, 249-255. doi: 10.1093/oxfordjournals.pcp.a028939
Sehar Z., Gautam H., Iqbal N., Alvi A.F., Jahan B., Fatma M., Albaqami M., Khan N.A., 2022. The functional interplay between ethylene, hydrogen sulfide, and sulfur in plant heat stress tolerance. Biomolecules 12, 678. https:// doi.org/10.3390/biom12050678
Shekhawat U.K., Ganapathi T.R., Srinivas L., 2011. Cloning and characterization of a novel stress-responsive WRKY transcription factor gene (*MusaWRKY71*) from *Musa* spp. cv. Karibale Monthan (ABB group) using transformed banana cells. Mol. Biol. Rep. 38, 4023-4035. doi: 10.1007/s11033-010-0521-4
Shi C.L., Stenvik G.E., Vie A.K., Bones A.M., Pautot V., Proveniers M., Aalen R.B., Butenko M.A., 2011. Arabidopsis class I KNOTTED-like homeobox proteins act downstream in the IDA-HAE/HSL2 floral abscission signaling pathway. Plant Cell 23, 2553-2567. doi: 10.1105/tpc.111.084608
Simpson S.D., Nakashima K., Narusaka Y., Seki M., Shinozaki K., Yamaguchi-Shinozaki K., 2003. Two different novel cis-acting elements of erd1, a clpA homologous Arabidopsis gene function in induction by dehydration stress and dark-induced senescence. Plant J. 33, 259-270. doi: 10.1046/j.1365-313x.2003.01624.x
Smalle J., Kurepa J., Haegman, M., Gielen J., Van Montagu M., Van Der Straeten D., 1998. The trihelix DNA-binding motif in higher plants is not restricted to the transcription factors GT-1 and GT-2. Proc. Natl. Acad. Sci. USA 95, 3318-3322. doi: 10.1073/pnas.95.6.331
Solano R., Nieto C., Avila J., Cañas L., Diaz I., Paz-Ares J., (1995. Dual DNA binding specificity of a petal epidermis-specific MYB transcription factor (MYB.Ph3) from *Petunia hybrida*. EMBO J. 14, 1773-1784. doi: 10.1002/j.1460-2075
Sun S., Wang B., Jiang Q., Li Z., Jia S., Wang Y., Guo H., 2021. Genome-wide analysis of *BpDof* genes and the tolerance to drought stress in birch (*Betula platyphylla*). PeerJ. 9, e11938. doi: 10.7717/peerj.11938
Trindade M., Tada M., Smith, J.C., 1999. DNA-binding specificity and embryological function of Xom (Xvent-2). Dev. Biol. 216, 442-456. doi: 10.1006/dbio.1999.9507
Terzaghi W.B., Cashmore A.R., 1995. Light-regulated transcription**.** Annu Rev. Plant Physiol. Plant Mol. Biol. 46, 445-474. https://doi.org/10.1146/annurev.pp.46.060195.002305
Urao T., Yamaguchi-Shinozaki K., Urao S., Shinozaki K., 1993. An Arabidopsis MYB homolog is induced by dehydration stress and its gene product binds to the conserved MYB recognition sequence. Plant Cell 5, 1529-1539. doi: 10.1105/tpc.5.11.1529
von Gromoff E.D., Schroda M., Oster U., Beck C.F., 2006. Identification of a plastid response element that acts as an enhancer within the *Chlamydomonas* HSP70A promoter. Nucleic Acids Res. 34, 4767-4779. doi: 10.1093/nar/gkl602
Wu G., Tian N., She F., Cao A., Wu W., Zheng S., Yang N., 2022. Characteristics analysis of *Early-Responsive-to-Dehydration* genes in *Arabidopsis thaliana* (*AtERD*). Plant Signal. Behav. 2, 2105021. doi: 10.1080/15592324.2022.2105021
Xie Z., Zhang Z.L., Zou X., Huang J., Ruas P., Thompson D., Shen Q.J., 2005. Annotations and functional analyses of the rice WRKY gene superfamily reveal positive and negative regulators of abscisic acid signaling in aleurone cells. Plant Physiol. 137, 176-189. doi: 10.1104/pp.104.054312
Yu H., Ma Y., Lu Y., Yue J., Ming, R., 2021. Expression profiling of the Dof gene family under abiotic stresses in spinach. Sci. Rep. 11, 14429. doi: 10.1038/s41598-021-93383-6
Zhang H., Huang Z., Xie B., Chen Q., Tian X., Zhang X., Zhang H., Lu X., Huang D., Huang R., 2004a. The ethylene-, jasmonate-, abscisic acid- and NaCl-responsive tomato transcription factor JERF1 modulates expression of GCC box-containing genes and salt tolerance in tobacco. Planta 220, 262-270. doi: 10.1007/s00425-004-1347-x
Zhang Z.L., Xie Z., Zou X., Casaretto J., Ho T.H., Shen Q.J., 2004b. A rice WRKY gene encodes a transcriptional repressor of the gibberellin signaling pathway in aleurone cells. Plant Physiol. 134, 1500-1513. doi: 10.1104/pp.103.034967
Zhao H., Wang Y., Zhao S., Fu Y., Zhu L., 2021. HOMEOBOX PROTEIN 24 mediates the conversion of indole-3-butyric acid to indole-3-acetic acid to promote root hair elongation. New Phytol. 232, 2057-2070. https://doi.org/10.1111/nph.17719

Table S3. Early transcriptional responses (1 and 5 h) to heat shock in *AhHAB4-PAI-1* and *Ah2880* overexpressing *A. thaliana* plants.

***Ah2880*: groups 6 and 10**	***AhHAB4-PAI-1*: group 8**
**Category 1: Group-defining transcription factors**	
MYB52 (AT1G17950); ETHYLENE WAX INDUCER 1 (AT1G15360); DRE BINDING PROTEIN 1B (AT4G25490); ETHYLENE RESPONSE FACTOR 7 (ERF7, AT3G20310); DREB subfamily A-4 (AT1G01250).	bZIP62 (AT1G19490); MYB DOMAIN PROTEIN 96 (AT5G62470); NUCLEAR FACTOR Y, SUBUNIT A5 (AT1G54160).
**Associated transcription factors/ transcription regulators**	
NGATHA-LIKE PROTEIN 3 (AT5G06250; Guo et al. 2020); ERF 018 (AT1G74930), ERF61 (AT1G64380) and DREB2A (AT2G40350) (Hwang et al. 2012; Wu et al. 2022); RHOMBOID-LIKE 2 (RBL2), a protease that mediates the release of the ANAC013 TF release under hypoxia (Eysholdt-Derzsó et al. 2023).	
**Category 2: Heat tress response-proteins including HSFs, HSPs and co-chaperones, protein modifications and others**	
PLANT U-BOX 18 (AT1G10560) a U-box and an ARM domain protein that has E3 ubiquitin ligase activity; RING/U-box superfamily protein (AT4G13100); COP1-INTERACTING PROTEIN 8 (AT5G64920) (Kim et al., 2016).	Carboxylate clamp (CC)-tetratricopeptide repeat (TPR) proteins (AT4G12400; AT5G48570, AT1G62740, AT3G25230) (Mishra et al., 2018); heat shock factors and proteins HSF7 (AT4G11660), HSFA2 (AT2G26150), HSP18.5 (AT2G19310), HSP60 (AT3G23990), HSP70T-2 (AT2G32120); HSP89.1 (AT3G07770); ATJ3 (AT3G44110); chaperone DnaJ-domain proteins (AT1G71000 AT2G33735); RAB geranylgeranyl transferase alpha subunit 2 (AT5G41820) (Ku et al., 2022); START lipid-binding domain-containing protein (AT5G07260) (Zhang et al., 2022); SOC3 protein (AT1G17600; (Zhang et al., 2017; Kim et al., 2021); RING/U-box superfamily protein (AT1G55530).
**Category 3: Regulation of HS responses involving DNA repair non-Coding RNAs, epigenetic regulation, DNA methylation, histone modification, chromatin remodeling, epigenetic memory and RNA alternative splicing**	
	Small nucleolar RNA genes (AT4G39366 AT4G39361 AT4G39364; Rodor et al. 2010); serine/arginine rich-like protein, *SR45a* (AT1G07350; Ling et al. 2021); DNA double-strand break repair *RAD50* ATPase (AT5G12900; Syed et al. 2018).
**Category 4: Cell wall/membrane modifications and ROS-associated responses**	
CASPARIAN STRIP MEMBRANE DOMAIN PROTEIN 1 (AT2G36100), recruiter of lignin-biosynthetic enzymes for Casparian strip formation in roots (Yang et al. 2022); P bodies *NAC SECONDARY WALL THICKENING PROMOTING FACTOR1* (AT4G25670) participates in the regulation of secondary cell wall formation in vascular tissues (Zhang et al. 2020); O- glycosyl hydrolases family 17 protein (AT1G66250) that includes many cellulose-degrading enzymes; three *3-KETOACYL-COA SYNTHASE* genes (AT2G26250, AT5G43760 and AT1G07720), that are part of the endoplasmic reticulum (ER) multienzyme complex involved in the synthesis of long- and very-long chain fatty acids wax precursors (Joubès et al. 2008; Liu et al., 2022); *LTPG1* (AT1G27950), lipid transfer protein having a glycosylphosphatidylinositol-anchor domain that promotes cuticular lipid accumulation (Lee et al. 2009); *CER*1 (AT1G02205), a wax biosynthetic enzyme that may catalyze key chemical conversions between long-chain aldehydes, alkanes, secondary alcohols and ketones (Rahman et al. 2021; Shaheenuzzamn et al. 2021); *POLYGALACTURONASE 1* (AT1G60390); *MUC169*	*GOLS2* galactinol synthase 2 (AT1G56600) raffinose family oligosaccharide (RFOs) biosynthetic enzyme, presumably involved in the mitigation of stress- induced growth inhibition (Salvi et al. 2018).

(AT1G27440) or Tubby-like protein 2 involved in mucilage polysaccharide biosynthesis (Wang et al. 2019); *HYR1* (AT3G21760), an inhibitor of cell expansion (Zhao et al. 2022); *DELTA (2)-ENOYL COA ISOMERASE 3* (AT4G14440) that degrades unsaturated fatty acids and triacylglycerols in response to heat stress to alter membrane lipid composition or to channel fatty acids to peroxisomal ß-oxidation for energy and amino acid production (Shiva et al. 2020; Korte et al. 2023); *NUDIX HYDROLASE HOMOLOG 24* (AT5G19470) that regulates stress responses through the maintenance of NAD+ and ATP levels (Ogawa et al. 2009); *miRNA775A* (AT1G78206) whose regulation of *GALACTOSYLTRANSFERASE 9* enhances the recovery from submergence stress by reducing RBOHD activity and ROS accumulation (Mishra et al. 2022) and plant *L-ASCORBATE OXIDASE* (AT5G21100).	
**Category 5: Transport, Carbon/ nitrogen metabolism and secondary metabolism**	
Phosphorus (P) stress-inducible *DUF506* gene family member (AT3G07350); *ATP BINDING CASSETTE (ABC) B2*; P-glycoprotein 2 (AT4G25960; Zhang et al. 2012). COPPER TRANSPORTER 1 (AT5G59030) and Copper ion binding / electron carrier protein (AT1G45063; Printz et al. 2016; Kumar et al. 2021). Dihydroorotate dehydrogenase (AT5G23300) involved in the pyrimidine reductive catabolic pathway activated in response to abiotic stress (Lopez et al. 2023).	Large subunit of *RUBISCO* (ATCG00490); *PSII L* protein (ATCG00560); *UREASE ACCESSORY PROTEIN* (AT2G35035; (Polacco et al. 2013).
**Category 6: Ribosomal, chloroplast- and mitochondria-associated proteins**	
RBD1 (AT1G54500), a thylakoid membrane-bound iron-binding protein required for the proper assembly of photosystem II (Song et al. 2021); CAAX amino terminal protease family protein (AT2G20725), that participates in the formation and maintenance of the chloroplast genetic system; HSP40 family protein containing a CAAX box (At3g44110) recently found to be required to mediate the protein farnesylation-dependent response to heat stress (Wu et al. 2019; Dalal 2022).	Chloroplast ribosomal proteins s2 and s12 (ATCG00160, ATCG00065; Hu et al. 2020); Armadillo repeat protein interacting with chloroplast chaperone CLPC1 (AT1G23180).
**Category 7: Growth, development and phytohormone-related events**	
RECEPTOR-LIKE PROTEIN KINASE 2, RPK2 (AT3G02130), involved in CLV3-mediated peptide regulation meristem differentiation and proliferation (Xie at al., 2022; Zhu et al., 2023); ATP BINDING CASSETTE B2: P-glycoprotein 2 (AT4G25960; (Zhang et al. 2012); ABA-mediated RESPONSIVE TO DEHYDRATION 22 (AT5G25610).	ABA 8'-hydroxylase (AT5G45340) whose expression levels define drought tolerance through ABA-related control of the transpiration rate (Iqbal et al. 2021; Sinha et al. 2023); AtCKX7 (AT3G63440; Lubovská et al. 2014; Liu et al. 2023); SGT1b-like protein (AT4G23570; Zhang et al. 2015).
**Category 8: High MW complex formation**	
Ankyrin repeat family proteins (AT5G50140, AT5G04730).	Subunit of ATPase complex CF0 (ATCG00150); ATP synthase, beta, delta and epsilon subunits (ATCG00480 ATCG00470) of the mitochondrial ATP synthase complex required for growth and heat stress tolerance (Liu et al. 2021).

Dalal, V. K. (2022). Transcriptome of laser micro dissected tissue from SAM of tomato reveals genes responsible for chloroplast biogenesis. Int. J. Agri. Res. Env. Sci. 2: 1-6. doi: 10.51626/ijares.2022.03.00014.

Eysholdt-Derzsó, E., Renziehausen, T., Frings, S., Frohn, S., von Bongartz, K., Igisch, C.P., Mann, J., Häger, L., Macholl, J., Leisse, D., et al. (2023). Endoplasmic reticulum-bound ANAC013 factor is cleaved by RHOMBOID-LIKE 2 during the initial response to hypoxia in *Arabidopsis thaliana*. Proc. Natl. Acad. Sci. USA. 120: e2221308120. doi: 10.1073/pnas.2221308120

Guo T, Wang S, Li Y, Yuan J, Xu L, Zhang T, Chao Y, and Han L. (2020). Expression of a *NGATHA1* gene from *Medicago truncatula* delays flowering time and enhances stress tolerance. Int. J. Mol. Sci. 21:2384. doi: 10.3390/ijms21072384.

Hu, S., Ding, Y., and Zhu, C. (2020). Sensitivity and responses of chloroplasts to heat stress in plants. Front. Plant Sci. 11: 375. doi: 10.3389/fpls.2020.00375

Hwang, J.E., Lim, C.J., Chen, H., Je, J., Song, C., and Lim, C.O. (2012). Overexpression of *Arabidopsis* dehydration- responsive element-binding protein 2C confers tolerance to oxidative stress. Mol. Cells. 33: 135-140. doi: 10.1007/s10059-012-2188-2.

Iqbal, N., Umar, S., Khan, N.A., and Corpas, F.J. (2021). Crosstalk between abscisic acid and nitric oxide under heat stress: exploring new vantage points. Plant Cell Rep. 40: 1429-1450. doi: 10.1007/s00299-021-02695-4.

Joubès, J., Raffaele, S., Bourdenx, B., Garcia, C., Laroche-Traineau, J., Moreau, P., Domergue, F., and Lessire, R. (2008). The VLCFA elongase gene family in *Arabidopsis thaliana*: phylogenetic analysis, 3D modelling and expression profiling. Plant Mol. Biol. 67: 547-66. doi: 10.1007/s11103-008-9339-z
Kim, M.G., Macoy, D.M., Lee, J.Y., Cha, J.Y., and Kim, W.Y. (2021). Interactions between plant immunity, temperature, light, and circadian rhythm. J. Plant Biochem. Physiol. 9: 260.
Kim, J.Y., Jang, I.C., and Seo, H.S. (2016). COP1 controls abiotic stress responses by modulating AtSIZ1 function through Its E3 ubiquitin ligase activity. Front. Plant Sci. 7: 1182. doi: 10.3389/fpls.2016.01182.
Korte, P., Unzner, A., Damm, T., Berger, S., Krischke, M., and Mueller, M.J. (2023). High triacylglycerol turnover is required for efficient opening of stomata during heat stress in *Arabidopsis*. Plant J. 115: 81-96. doi: 10.1111/tpj.16210.
Ku, Y.S., Cheng, S.S., Cheung, M.Y., Law, C.H., and Lam, H.M. (2022). The re-localization of proteins to or away from membranes as an effective strategy for regulating stress tolerance in plants. Membranes (Basel) 12: 1261. doi: 10.3390/membranes12121261.
Kumar, V., Pandita, S., Singh Sidhu, G.P., Sharma, A., Khanna, K., Kaur, P., Bali, A.S., and Setia, R. (2021). Copper bioavailability, uptake, toxicity and tolerance in plants: A comprehensive review. Chemosphere 262: 127810. doi: 10.1016/j.chemosphere.2020.127810
Lee, S.B., Go, Y.S., Bae, H.J., Park, J.H., Cho, S.H., Cho, H.J., Lee, D.S., Park, O.K., Hwang, I., and Suh, M.C. (2009). Disruption of glycosylphosphatidylinositol-anchored lipid transfer protein gene altered cuticular lipid composition, increased plastoglobules, and enhanced susceptibility to infection by the fungal pathogen Alternaria brassicicola. Plant Physiol. 150:42-54. doi: 10.1104/pp.109.137745.
Ling, Y., Mahfouz, M.M., Zhou, S. (2021) Pre-mRNA alternative splicing as a modulator for heat stress response in plants. Trends Plant Sci. 26: 1153-1170. doi: 10.1016/j.tplants.2021.07.008.
Liu, T., Arsenault, J., Vierling, E., and Kim, M. (2021). Mitochondrial ATP synthase subunit d, a component of the peripheral stalk, is essential for growth and heat stress tolerance in Arabidopsis thaliana. Plant J. 107: 713-726. doi: 10.1111/tpj.15317.
Liu, M., Cui, Y., Peng, F., Wang, S., Cui, R., Liu, X., Zhang, Y., Huang, H., Fan, Y., Jiang, T., et al. (2023). Antioxidant system was triggered to alleviate salinity stress by cytokinin oxidase/dehydrogenase gene GhCKX6b-Dt in cotton. Environ. Sci. Eur. 35: 82. https://doi.org/10.1186/s12302-023-00788-3.
Liu, X., Zhao, Z., Yang, Y., Xu, H., Bi, Q., and Wang, L. (2022). Genome-wide identification and expression analysis of the KCS gene family in yellow horn reveal their putative function on abiotic stress responses and wax accumulation. Horticulturae 9: 25. https://doi.org/10.3390/horticulturae9010025.
Lopez, A.J., Narvaez-Ortiz, H.Y., Rincon-Benavides, M.A., Pulido, D.C., Fuentes Suarez, L.E., and Zimmermann, B.H. (2023). New Insights into rice pyrimidine catabolic enzymes. Front. Plant Sci. 14: 1079778. doi: 10.3389/fpls.2023.1079778. PMID: 36818891.
Lubovská Z, Dobrá J, Storchová H, Wilhelmová N, Vanková R. (2014). Cytokinin oxidase/dehydrogenase overexpression modifies antioxidant defense against heat, drought and their combination in Nicotiana tabacum plants. J. Plant Physiol. 171: 1625-1633. doi: 10.1016/j.jplph.2014.06.021
Mishra, D., Shekhar, S., Chakraborty, S., and Chakraborty, N. (2018). Carboxylate clamp tetratricopeptide repeat (TPR) domain containing Hsp90 cochaperones in Triticeace: An insight into structural and functional diversification. Environ. Exp. Bot. 155: 31-44. https://doi.org/10.1016/j.envexpbot.2018.06.020
Mishra, V., Singh, A., Gandhi, N., Sarkar Das, S., Yadav, S., Kumar, A., Sarkar, A.K. (2022). A unique miR775-GALT9 module regulates leaf senescence in Arabidopsis during post-submergence recovery by modulating ethylene and the abscisic acid pathway. Development 149: dev199974. doi: 10.1242/dev.199974.
Ogawa, T., Ishikawa, K., Harada, K., Fukusaki, E., Yoshimura, K., and Shigeoka, S. (2009). Overexpression of an ADP-ribose pyrophosphatase, AtNUDX2, confers enhanced tolerance to oxidative stress in Arabidopsis plants. Plant J. 57: 289-301. doi: 10.1111/j.1365-313X.2008.03686.x
Polacco, J.C., Mazzafera, P., and Tezotto, T., (2013). Opinion: nickel and urease in plants: still many knowledge gaps. Plant Sci. 199-200: 79-90. doi: 10.1016/j.plantsci.2012.10.010. Printz, B., Lutts, S., Hausman, J.F., and Sergeant, K. (2016). Copper trafficking in plants and its implication on cell wall dynamics. Front. Plant Sci. 7: 601. doi: 10.3389/fpls.2016.00601.
Rahman, T., Shao, M., Pahari, S., Venglat, P., Soolanayakanahally, R., Qiu, X., Rahman, A., and Tanino, K. (2021). Dissecting the roles of cuticular wax in plant resistance to shoot dehydration and low-temperature stress in Arabidopsis. Int. J, Mol. Sci. 22 :1554. doi: 10.3390/ijms22041554.
Rodor, J., Letelier, I., Holuigue, L., and Echeverria, M. (2010). Nucleolar RNPs: from genes to functional snoRNAs in plants. Biochem. Soc. Trans. 38: 672-676. doi: 10.1042/BST0380672.
Salvi, P., Kamble, N.U. and Majee, M. (2018). Stress-inducible galactinol synthase of chickpea (CaGOLS) is implicated in heat and oxidative stress tolerance through reducing stress-induced excessive reactive oxygen species accumulation. Plant Cell Physiol. 59: 155-166. doi: 10.1093/pcp/pcx170.
Shaheenuzzamn, M., Shi, S., Sohail, K., Wu, H., Liu, T., An, P., Wang, Z., and Hasanuzzaman, M. (2021). Regulation of cuticular wax biosynthesis in plants under abiotic stress. Plant Biotechnol. Rep. 15: 1-12. https://doi.org/10.1007/s11816-020-00656-z.
Shiva, S., Samarakoon, T., Lowe, K.A., Roach, C., Vu, H.S., Colter, M., Porras, H., Hwang, C., Roth, M.R., Tamura, P., et al. (2020). Leaf lipid alterations in response to heat stress of Arabidopsis thaliana. Plants (Basel) 9: 845. doi: 10.3390/plants9070845.
Sinha, R., Induri, S.P., Peláez-Vico, M.Á., Tukuli, A., Shostak, B., Zandalinas, S.I., Joshi, T., Fritschi, F.B., and Mittler, R. (2023). The transcriptome of soybean reproductive tissues subjected to water deficit, heat stress, and a combination of water deficit and heat stress. Plant J. 116: 1064-1080. doi: 10.1111/tpj.1622.
Song, Y., Feng, L., Alyafei, M.A.M., Jaleel, A., and Ren, M. (2021). Function of chloroplasts in plant stress responses. Int. J. Mol. Sci. 22: 13464. doi: 10.3390/ijms222413464.
Wang, M., Xu, Z., Ahmed, R.I., Wang, Y., Hu, R., Zhou. G., and Kong, Y. (2019). Tubby-like Protein 2 regulates homogalacturonan biosynthesis in Arabidopsis seed coat mucilage. Plant Mol. Biol. 99: 421-436. doi: 10.1007/s11103-019-00827-9.
Syed, A., Tainer, J.A. (2018) The MRE11-RAD50-NBS1 complex conducts the orchestration of damage signaling and outcomes to stress in DNA replication and repair. Annu. Rev. Biochem. 87: 263-294. doi: 10.1146/annurev-biochem-062917-012415.
Wu, J.R., Wang, T.Y., Weng, C.P., Duong, N.K.T., and Wu, S.J. (2019). AtJ3, a specific HSP40 protein, mediates protein farnesylation-dependent response to heat stress in Arabidopsis. Planta 250: 1449-1460. doi: 10.1007/s00425-019-03239-7.
Wu, Y., Li, X., Zhang, J., Zhao, H., Tan, S., Xu, W., Pan, J., Yang, F., and Pi, E. (2022). ERF subfamily transcription factors and their function in plant responses to abiotic stresses. Front. Plant Sci. 13: 1042084. doi: 10.3389/fpls.2022.1042084.
Xie, H., Zhao, W., Li, W., Zhang, Y., Hajný, J., and Han, H. (2022). Small signaling peptides mediate plant adaptions to abiotic environmental stress. Planta 255: 72. doi: 10.1007/s00425-022-03859- 6.
Yang, X., Xie, H., Weng, Q., Liang, K., Zheng, X., Guo, Y., and Sun, X. (2022). Rice OsCASP1 orchestrates Casparian strip formation and suberin deposition in small lateral roots to maintain nutrient homeostasis. Front. Plant Sci. 13: 1007300. doi: 10.3389/fpls.2022.1007300.
Zhang, X.C., Millet, Y.A., Cheng, Z., Bush. J, and Ausubel, F.M. (2015). Jasmonate signalling in Arabidopsis involves SGT1b-HSP70-HSP90 chaperone complexes. Nat. Plants 1: 15049. doi: 10.1038/nplants.2015.49.
Zhang, X., Li, J., Liu, A., Zou, J., Zhou, X., Xiang, J., Rerksiri, W., Peng, Y., Xiong, X., and Chen, X. (2012). Expression profile in rice panicle: insights into heat response mechanism at reproductive stage. PLoS ONE 7: e49652. doi: 10.1371/journal.pone.0049652
Zhang, Q., Luo, F., Zhong, Y., He, J., and Li, L. (2020). Modulation of NAC transcription factor NST1 activity by XYLEM NAC DOMAIN1 regulates secondary cell wall formation in Arabidopsis. J. Exp. Bot. 71: 1449-

1458. doi: 10.1093/jxb/erz513.
Zhang, Y., Wang, Y., Liu, J., Ding, Y., Wang, S., Zhang, X., Liu, Y., and Yang, S. (2017). Temperature-dependent autoimmunity mediated by chs1 requires its neighboring TNL gene SOC3. New Phytol. 213: 1330-1345. doi: 10.1111/nph.14216.
Zhang, H., Zhang, K., Liu, T., Zhang, Y., Tang, Z., Dong, J., and Wang, F. (2022). The characterization and expression analysis under stress conditions of PCST1 in Arabidopsis. Plant Signal. Behav. 17: 2134675. doi: 10.1080/15592324.2022.2134675
Zhao, Y., Qin, Q., Chen, L., Long, Y., Song, N., Jiang, H., and Si, W. (2022). Characterization and phylogenetic analysis of multiple C2 domain and transmembrane region proteins in maize. BMC Plant Biol. 22: 388. doi: 10.1186/s12870-022-03771-x
Zhu, Q., Feng, Y., Xue, J., Chen, P., Zhang, A., and Yu, Y. (2023) Advances in receptor-like protein kinases in balancing plant growth and stress responses. Plants (Basel) 12: 427. doi: 10.3390/plants1203

Table S4. Intermediate transcriptional responses (11 h) to heat shock in *AhHAB4-PAI-1* and *Ah2880* overexpressing *A. thaliana* plants.

***Ah2880*: group 7**	***AhHAB4-PAI-1*: groups 3 and 5**
**Category 1: Group-defining transcription factors**	
The homeodomain *OVEREXPRESSOR OF CATIONIC PEROXIDASE 3* (AT5G11270); *DREB* AND *EAR MOTIF PROTEIN 3* (AT2G23340); *DREB2A* (AT5G05410).	*FLOWERING bHLH 3* (AT1G51140); *DREB1A* (AT4G25480); *MYB94* (AT3G47600); C2H2-type zinc finger *YIN YANG 1* TF (AT4G06634); *bZIP12/ ENHANCED EM LEVEL* (AT2G41070); *ANAC72* (AT4G27410); *DC3 PROMOTER-BINDING FACTOR 5* (AT4G34000).
**Associated transcription factors/ transcription regulators**	
MYB1 (AT3G09230); Zinc finger/*BTB DOMAIN PROTEIN* (AT1G33810); *HIT- TYPE* Zinc finger family protein (AT5G63830); *PAPA-1-like family protein* / zinc finger (HIT type) family protein (AT3G0666; Li et al. 2014).	*DREB1C* (AT4G25470); *CYTOKININ RESPONSE FACTOR 2/ TARGET OF MONOPTEROS 3* (AT4G23750) and *TITANIA 2* (AT3G63500) (Hallmark and Rashotte 2019; Chen et al. 2024); *bHLH2/ SAWTOOTH1* (AT4G36870; He et al. 2022); *MYB3R1* (AT4G32730); *AGAMOUS-LIKE MADS- box protein* (AT2G41440); *bZIP70* (AT5G60830); *bZIP G-BOX BINDING FACTOR 3 TF* (AT2G46270); NAC-domain TF gene (AT4G36160) expressed in developing xylem; C2H2 zinc finger TF gene (AT3G05760); *PLETHORA 5/ AINTEGUMENTA-LIKE 5* (AT5G57390), an AP2 TF that mediates the transition between the embryonic and vegetative phases (Han et al., 2022); *AGAMOUS-LIKE 16* (AT3G57230); HOMEOBOX 53 (AT5G66700); *EARLY FLOWERING 3*, a transcriptional repressor involved in temperature sensing and thermo-morphogenesis (Jung et al. 2020).
**Category 2: Heat tress response-proteins including HSFs, HSPs and co-chaperones, protein modifications and others**	
PLANT U-BOX 48 (AT5G18340; Zhou et al., 2024); *HSP70-17* (AT4G16660); vacuolar protein sorting-associated protein (AT5G43950); gene (AT5G17400; Zhang et al. 2016); a carboxylate clamp (CC)-tetratricopeptide repeat (TPR) protein (AT1G62390) able to interact with Hsp90/Hsp70 as a co-chaperone (Mishra et al. 2018).	*MAG2* protein (AT3G47700) involved in ER-to-Golgi anterograde and intra-Golgi trafficking (Ren et al. 2022); *VPS54 HOMOLOG* (AT4G19490; Wang et al., 2011); *PATELLIN2* (AT3G55480) a phosphatidylinositol transfer protein involved in the biogenesis and function of lytic vacuoles (Zhou et al. 2019); endosomal targeting *BRO1-like* domain- containing protein (AT1G73390) that regulates endosomal membrane function (Mehdi et al. 2023); *Got1/Sft2-like* vesicle transport protein family (AT3G49420) a Golgi localized membrane protein that interact with SNARE proteins to regulate the fusion to vesicle target membranes (Hertig et al. 2023); F-box proteins that recognize the Skp1-Rbx1-Cul1-F-box protein ubiquitin ligases (AT3G62430, AT4G14103, AT3G03030) (Shao et al. 2013; Qu et al., 2020); several *RING/U-box*, *RING/FYVE/PHD* and *RING-H2* superfamily protein genes (AT2G01150, AT1G15165, AT4G09130, AT2G35420), a ubiquitin carboxyl-terminal hydrolase family protein gene (AT5G45790) and *PUB19* an ARA-responsive U-box Armadillo repeat protein gene (AT1G60190) having E3 ubiquitin ligase activity (Bergler and Hoth 2011); *NEDD8/RUB*-activating enzyme (AA042766; Wei et al. 2023); *ATE1* (AT5G05700; Deka et al. 2021; Zhang et al. 2024); *EMBRYO DEFECTIVE 3012* (AT5G40480) that together with SET1, a histone 3 Lys 4 methyl transferase, control thermogenic flowering time (Ma et al. 2016); several members of the TPR-like superfamily protein genes (AT2G24310, AT2G27800, AT3G15130, AT5G14080,

	AT2G15980, AT1G53600, AT2G37320, AT3G15250) that mediate protein interactions regulating development-, phytohormone- and stress-responsive processes including protein transport, folding and/or damaged-protein degradation (Sharma and Pandey 2016).
**Category 3: Regulation of HS responses involving DNA repair non-Coding RNAs, epigenetic regulation, DNA methylation, histone modification, chromatin remodeling, epigenetic memory and RNA alternative splicing**	
MEDIATOR 19B of RNA polymerase II transcription subunit (AT5G19480; Crawford et al. 2024); *GLYCINE-RICH RNA-BINDING PROTEIN* (AT4G10330; Ma et al. 2021); *N-LYSINE METHYLTRANSFERASE* (AT1G78150; Zhou et al. 2020).	*DAMAGED DNA BINDING PROTEIN 1B* (AT4G21100; Schalk et al. 2016; Ban and Estelle 2021); *Agenet/Tudor* domain- containing protein gene (AT1G26540; Brasil et al. 2015); *AtTHO5* (AT5G42920; Khan et al. 2020); *SPT5-like* protein (AT5G04290; Wang and Dennis 2009*); POLYNUCLEOTIDE ADENYLYLTRANSFERASE* (AT5G23690) that catalyzes nucleotide modifications at the 3' end of RNA to regulate RNA and small RNAs stability (Kang et al. 2024); *ATRX-like* protein (AT5G53930; Hermann et al. 2004); *EARLY FLOWERING 8* (AT2G06210; Perrella et al. 2022); Pcf11p-similar protein 4 PCFS4 (AT4G04885; Xing et al. 2008; Shao et al. 2021); *MITOCHONDRIAL SPLICING FACTOR 2* (AT3G22670; Wang et al. 2018); a switch/sucrose non-fermentable *SNF2* family of helicase-like proteins (AT3G20010) involved in RNA-directed DNA methylation and inhibition of miRNA production (Liu et al., 2022), several PPRPs RNA editing proteins (AT1G02420, AT1G14470, AT2G17525, AT2G03380, AT5G08510, AT2G19280) including six others localized in either the chloroplasts (AT3G46790, AT5G08305) and mitochondria (AT2G20540, AT3G13880, AT1G56570, AT5G09950); *NUCLEOLIN-LIKE* protein (AT2G36420) that increases nucleolar activity and ribosome assembly (Darriere et al. 2022); three microRNAs known to re-program the transcription of genes coding for AP2/ERF TFs (miR172B, AT5G04275), CC-NBS-LRR receptors (miR472, AT1G12294) and auxin responsive TFs (miR160B, AT4G17788) in heat stressed tomato plants (Keller et al. 2020); *SILENCING MOVEMENT DEFICIENT 1* (AT4G11130; Brosnan and Voinnet 2011); *HEN1* (AT4G20910; Zuo et al. 2021; Singh et al. 2022); a subunit of *RNA POLYMERASE III/ RNA POLYMERASE C* (AT5G60040; Wu et al. 2012); *FORKHEAD-ASSOCIATED DOMAIN*-containing protein (AT5G07400; Mahajan et al. 2008); YTHDF protein (AT5G61020) that recognizes N6-methyladenosine in mRNAs for gene regulation (Arribas-Hernández et al. 2021); polynucleotidyl transferase, a ribonuclease H fold protein (AT2G32415; Arae et al. 2019); tRNA/rRNA methyltransferase (AT4G17610; Ramakrishnan et al. 2022; Ohira and Suzuki. 2024); polyadenylate-binding protein (AT2G36660); LIKE AMP1 (AT5G19740; Nobusawa et al. 2021).
**Category 4: Cell wall/membrane modifications and ROS-associated responses**	
TRICHOME BIREFRINGENCE-LIKE protein (AT1G60790) involved in the synthesis and deposition of secondary wall cellulose (Sun et al. 2020); N-*ACETYLGLUTAMATE KINASE* (AT3G57560; Shao et al. 2022).	*CELLULOSE SYNTHASE INTERACTIVE 2* (AT1G44120; Kesten et al. 2017); KCS21 (AT5G49070); cell wall-localized class III peroxidase (AT2G22420); *TRICHOME BIREFRINGENCE-LIKE* (AT5G15890; Li et al. 2023); *CSLA2*, a beta- mannan synthase (AT5G22740) that synthesizes the backbone of galactoglucomannans needed for mucilage architecture (Verhertbruggen et al. 2011): *ACYL-COA THIOESTERASE* (AT4G00520) that converts fatty acyl thioesters to free fatty acids (Kalinger et al. 2020); *AtGGH2* (AT1G78680; Gorelova et al. 2017).
**Category 5: Transport, Carbon/ nitrogen metabolism, secondary metabolism**	
GALACTOSE MUTAROTASE-LIKE superfamily protein (AT5G66530) involved in glycolysis, and responsive to flooding stress (Qiao et al. 2020).	*MAJOR FACILITATOR SUPERFAMILY PROTEIN* (AT1G72125); *BORIC ACID CHANNEL* (AT4G10380) preferentially localized in outer membrane domains of root cells. *PHOSPHOPANTOTHENOYLCYSTEINE DECARBOXYLASE* subunit (AT3G15357), a precursor of pantothenic acid and CoA required for fatty acid metabolism, the tricarboxylic acid cycle, and others (Yang et al. 2020); *CYTIDINE TRIPHOSPHATE SYNTHASE* (AT4G02120; Krämer et al. 2022); *O-GLYCOSYL HYDROLASES* family 17 (AT5G58480)

	involved in starch and sucrose metabolism; *PSII K* (ATCG00070); *GLUTAMINE AMIDOTRANSFERASE TYPE 1* family protein (AT5G57890).
**Category 6: Ribosomal, chloroplast- and mitochondria-associated proteins**	
RPL1p/L10e (AT1G08360); *RPL6* (AT1G33120); *RPL7A*e/L30e/S12e (AT2G47610); *RPL9* (AT1G33140); *RPL14p/L23e* (AT1G04480); *RPL18* (AT3G05590); *RPL19* (AT5G47190 and AT4G17560); *RPL21* (AT1G35680); *RPL22p/L17e* (AT1G27400) and *RPL30/L7* (AT2G44120); *RIBOSOMAL PROTEIN CS22* (AT3G52150), that provides enhanced tolerance to abiotic stress and has RNA chaperone activity (Robles and Quesada 2022); *JANUS* (AT2G18510) thar regulates embryonic pattern formation *via* WUSCHEL RELATED HOMEOBOX TF; auxin transport-related *PIN- FORMED7* (Xiong et al. 2019) also found to have RNA and protein chaperone activity in bacteria (Kovacs et al. 2009).<br>Thylakoid protein gene expressed during early development (AT1G62780; Fristedt 2017); plastid isoform of the *PHOSPHOGLUCOMUTASE* enzyme (AT5G51820) that needed for both starch synthesis and degradation to control carbon flux and triacylglycerol accumulation in oilseed plants (Periappuram et al. 2000); *SUPPRESSOR OF VARIEGATION 7* (AT4G16390), a pentatricopeptide repeat protein (PPR) required for FtsH- mediated chloroplast biogenesis (Robles and Quezada 2022); *MALONYL-ACP* (AT2G30200; Arzac et al. 2022); *CHLOROPLAST RNA-BINDING PROTEIN 31B* (AT5G50250) that stabilizes plastid mRNA during seedling development (Zeng et al. 2014); chloroplast-localized *CHAPERONIN 10* (AT2G44650; Pareek et al. 2021); plastidial *THIOREDOXIN* (TRX) isoform (AT3G06730; Zagorchev et al. 2013); chloride ion channel gene located in the stroma thylakoid membrane (AT3G61320) that adjusts electron transport as part of photo-protective mechanisms (Herdean et al. 2016); *PROHIBITIN 2* (AT1G03860; Herdean et al. 2016); *AtNG1* (AT1G62720; Yang et al. 2011; Wei and Han 2016); *RHOMBOID-like protein 4* (AT3G53780) a major class of chloroplast proteases that cleave intrinsic inner envelope proteins (Knopf and Adam 2012; Lavell et al. 2019).	*SIGNAL PEPTIDE PEPTIDASE 1* (AT1G73990; Trösch et al. 2022); *TPR PLASTID PROTEIN FACTOR* (AT3G63370; Hao et al. 2021).
**Category 7: Growth, development and phytohormone-related events**	
CIS-CINNAMIC ACID RESPONSIVE gene (AT2G01520; Guo et al. 2011; Li et al. 2023); plasmodesma (PD)-localized *FT INTERACTING PROTEIN 3* (AT3G57880) has close contact with the ER– plasma membrane to control transport and signaling during plant development (Zhao et al. 2022); *EVOLUTIONARILY CONSERVED C- TERMINAL REGION 1* (ECT1, AT3G03950; Ok et al. 2005); *CONSTANS-LIKE 3* (AT2G24790; Casal and Balasubramanian 2019); *CHCH DOMAIN PROTEIN* (AT5G64400; Wang et al. 2021).	*RGFR3* (AT3G26540; Ou et al. 2022); *BUDDHA'S PAPER SEAL 2* (AT2G21480; Zhu et al. 2018; Yu et al. 2022); *CLAVATA3/ ESR-related 23* (AT1G69970; Miwa et al. 2009); *ARABIDILLO-1* (AT2G44900; Mu et al. 2010); *TIC-LIKE PROTEIN* (AT3G63180; Qu et al. 2023); *CML16*, a calmodulin like protein (AT1G18530; Jung et al. 2017); *OXYSTEROL BINDING PROTEIN-RELATED PROTEIN 2B* (AT4G12460) a membrane constituent involved in the biosynthesis of steroid hormones or cell wall precursors (Saravanan et al. 2009); *PROTEIN PHOSPHATASE 2C* (AT3G11410) and clade A *PROTEIN PHOSPHATASE TYPE 2C* (AT5G59220); *AtCKX5* (AT5G21482); *TAGK2* protein kinase (AT3G19100); *AUXIN RESPONSE FACTOR 18* (AT3G61830; Du and Scheres 2018); *RPK1* (AT1G69270; Osakabe et al. 2010); *ABI five binding protein 3* (AT3G29575); *ABA INSENSITIVE 1* (AT4G26080); *ATP-BINDING CASETTE G25* (AT1G71960); *SNF1-RELATED PROTEIN KINASE 2* (AT4G40010; Praat et al., 2021); *ABA-INSENSITIVE PROTEIN KINASE 1* (AT3G50310).
**Category 8: High MW complex formation**	
ACTIN-BINDING FH2 (formin homology 2) family protein (AT3G05470).	Two *DEAD-BOX HELICASES* (AT2G40700 and AT4G14790) and *STRESS RESPONSE*

	SUPPRESSOR1, a DExH box helicase (AT1G31970; Kant et al. 2007; Kalinina et al. 2018); two RRM/RBD/RNP RNA binding protein genes (AT2G28540 and AT1G31600) and an additional GLYCINE-RICH RNA BINDING protein gene(AT4G27850; Yan et al. 2022); *EXONUCLEASE FAMILY PROTEIN* (AT2G48100), similar to *EXONUCLEASE 4* (Weber et al., 2008); *ATP BINDING MICROTUBULE (MT) MOTOR FAMILY PROTEIN* (AT5G42490) that modulates MT organization and structural interaction of MTs with other cell structures (Krtková et al. 2016); *Nemp1/TMEM194A* (AT3G49840; Fang and Gu 2023); *MINIYO* (AT4G38440) a regulator of molecule trafficking to the nucleus (Muñoz-Díaz and Sáez-Vasquez 2022); WD40 protein genes (AT1G49450, AT5G19920, AT3G21540, AT5G43930) and ankyrin-repeat family protein genes (AT2G04740, AT2G39580) (Sharma and Pandey 2016).

Arae, T., Morita, K., Imahori, R., Suzuki, Y., Yasuda, S., Sato, T., Yamaguchi, J., and Chiba, Y. (2019). Identification of *Arabidopsis* CCR4-NOT complexes with Pumilio RNA-binding proteins, APUM5 and APUM2. Plant Cell Physiol. 60: 2015-2025. doi: 10.1093/pcp/pcz089.

Arribas-Hernández, L., Rennie, S., Schon, M., Porcelli, C., Enugutti, B., Andersson, R., Nodine, M.D., and Brodersen, P. (2021). The YTHDF proteins ECT2 and ECT3 bind largely overlapping target sets and influence target mRNA abundance, not alternative polyadenylation. Elife 10: e72377. doi: 10.7554/eLife.72377.

Arzac, M.I., Fernández-Marín, B., and García-Plazaola, J.I. (2022). More than just lipid balls: quantitative of plastoglobule attributes and their stress-related responses. Planta 255: 62. doi: 10.1007/s00425-022-03848-9.

Ban Z, and Estelle, M. (2021). CUL3 E3 ligases in plant development and environmental response. Nat. Plants 7: 6-16. doi: 10.1038/s41477-020-00833-Bergler, J., and Hoth, S. (2011). Plant U- box armadillo repeat proteins AtPUB18 and AtPUB19 are involved in salt inhibition of germination in *Arabidopsis*. Plant Biol. 13: 725-730. doi: 10.1111/j.1438-8677.2010.00431.x.

Brasil JN, Cabral LM, Eloy NB, Primo LM, Barroso-Neto IL, Grangeiro LP, Gonzalez, N., Inzé, D., Ferreira, P.C., and Hemerly, A.S. (2015). AIP1 is a novel Agenet/Tudor domain protein from *Arabidopsis* that interacts with regulators of DNA replication, transcription and chromatin remodeling. BMC Plant Biol. 15: 270. doi: 10.1186/s12870-015-0641-z.

Brosnan, C.A., and Voinnet, O. (2011). Cell-to-cell and long-distance siRNA movement in plants: mechanisms and biological implications. Curr. Opin. Plant Biol. 14: 580-587. doi: 10.1016/j.pbi.2011.07.011.
Casal, J.J., and Balasubramanian, S. (2019). Thermomorphogenesis. Annu. Rev. Plant Biol. 70: 321-346.

Chen, M., Dai, Y., Liao, J., Wu, H., Lv, Q., Huang, Y., Liu, L., Feng, Y., Lv, H., Zhou, B., et al. (2024). TARGET OF MONOPTEROS: key transcription factors orchestrating plant development and environmental response. J. Exp. Bot. 75: 2214-2234. doi: 10.1093/jxb/erae005.

Crawford, T., Siebler, L., Sulkowska, A., Nowack, B., Jiang, L., Pan, Y., Lämke, J., Kappel, C., and Bäurle, I. (2024). The Mediator kinase module enhances polymerase activity to regulate transcriptional memory after heat stress in *Arabidopsis*. EMBO J. 43: 437-461. doi: 10.1038/s44318-023-00024-x.

Deka, K., and Saha, S. (2021). Heat stress induced arginylation of HuR promotes alternative polyadenylation of Hsp70.3 by regulating HuR stability and RNA binding. Cell Death Differ. 28:730-747. doi: 10.1038/s41418-020-00619-5.

Darriere, T., Jobet, E., Zavala, D., Escande, M.L., Durut, N., de Bures, A., Blanco-Herrera, F., Vidal, E.A., Rompais, M., Carapito, C., et al. (2022). Upon heat stress processing of ribosomal RNA precursors into mature rRNAs is compromised after cleavage at primary P site in Arabidopsis thaliana. RNA Biol. 19: 719-734. doi: 10.1080/15476286.2022.2071517.

Du, Y., Scheres. B. (2018) Lateral root formation and the multiple roles of auxin. J. Exp. Bot. 69:155-167. doi: 10.1093/jxb/erx223. PMID: 28992266.

Fang, Y., and Gu, Y. (2023). Dynamic nucleoskeleton in stress. Nat Plants 9: 1010-1011. doi: 10.1038/s41477-023-01458-1.

Fristedt, R. (2017). Chloroplast function revealed through analysis of GreenCut2 genes. J. Exp. Bot. 68: 2111-2120. doi: 10.1093/jxb/erx082. PMID: 28369575.

Gorelova, V., De Lepeleire, J., Van Daele, J., Pluim, D., Meï, C., Cuypers, A., Leroux, O., Rébeillé, F., Schellens, J.H.M., Blancquaert, D., et al. (2017). Dihydrofolate reductase/thymidylate synthase fine-tunes the folate status and controls redox homeostasis in plants. Plant Cell 29 :2831-2853. doi: 10.1105/tpc.17.00433.

Guo, D., Wong, W.S., Xu, W.Z., Sun, F.F., Qing, D.J., and Li, N. (2011). Cis-cinnamic acid-enhanced 1 gene plays a role in regulation of Arabidopsis bolting. Plant Mol. Biol. 75: 481-495. doi: 10.1007/s11103-011-9746-4.

Herdean, A., Teardo, E., Nilsson, A.K., Pfeil, B.E., Johansson, O.N., Ünnep, R., Nagy, G., Zsiros, O., Dana, S., Solymosi, K., et al. (2016). A voltage-dependent chloride channel fine-tunes photosynthesis in plants. Nat. Commun. 7: 11654. doi: 10.1038/ncomms11654.

Hallmark, H.T., and Rashotte, A.M. (2019). Review-cytokinin response factors: Responding to more than cytokinin. Plant Sci. 289: 110251. doi: 10.1016/j.plantsci.2019.110251.

Han, X., Liu, K., Yuan, G., He, S., Cong, P., and Zhang, C. (2022). Genome-wide identification and characterization of AINTEGUMENTA-LIKE (AIL) family genes in apple (Malus domestica Borkh.). Genomics 114: 110313. doi: 10.1016/j.ygeno.2022.110313.

Hao, W., Liu, G., Wang, W., Shen, W., Zhao, Y., Sun, J., Yang, Q., Zhang, Y., Fan, W., Pei, S., et al. (2021). RNA editing and its roles in plant organelles. Front. Genet. 12: 757109. doi: 10.3389/fgene.2021.757109.

He, Y., Yang, T., Yan, S., Niu, S., and Zhang, Y. (2022). Identification and characterization of the BEL1-like genes reveal their potential roles in plant growth and abiotic stress response in tomato. Int. J. Biol. Macromol. 200 :193-205. doi: 10.1016/j.ijbiomac.2021.12.175.

Hermann, A., Gowher, H., and Jeltsch, A. (2004). Biochemistry and biology of mammalian DNA methyltransferases. Cell Mol. Life Sci. 61: 2571-2587. doi: 10.1007/s00018-004-4201-1.

Hertig, C., Rutten, T., Melzer, M., Schippers, J.H.M., and Thiel, J. (2023). Dissection of developmental programs and regulatory modules directing endosperm transfer cell and aleurone identity in the syncytial endosperm of barley. Plants (Basel)12: 1594. doi: 10.3390/plants12081594.

Jung, H., Chung, P.J., Park, S.H., Redillas, M.C.F.R., Kim, Y.S., Suh, J.W., and Kim, J.K. (2017). Overexpression of OsERF48 causes regulation of OsCML16, a calmodulin-like protein gene that enhances root growth and drought tolerance. Plant Biotechnol. J. 15: 1295-1308. doi: 10.1111/pbi.12716.

Jung, J.H., Barbosa, A.D., Hutin, S., Kumita, J.R., Gao, M., Derwort, D., Silva, C.S., Lai, X., Pierre, E., Geng, F., et al. (2020). A prion-like domain in ELF3 functions as a thermo-sensor in Arabidopsis. Nature 585: 256-260. doi: 10.1038/s41586-020-2644-7.

Kalinger, R.S., Pulsifer, I.P., Hepworth, S.R., and Rowland, O. (2020). Fatty Acyl synthetases and thioesterases in plant lipid metabolism: Diverse functions and biotechnological applications. Lipids 55: 435-455. doi: 10.1002/lipd.12226.

Kalinina, N.O., Makarova, S., Makhotenko, A., Love, A.J., and Taliansky, M. (2018). The multiple functions of the nucleolus in plant development, disease and stress responses. Front. Plant Sci. 9: 132. doi: 10.3389/fpls.2018.00132. Kang, L., Li, C., Qin, A., Liu, Z., Li, X., Zeng, L., Yu, H., Wang, Y., Song, J., and Chen, R. (2024). Identification and expression analysis of the Nucleotidyl Transferase Protein (NTP) family in soybean (Glycine max) under various abiotic stresses. Int. J. Mol. Sci. 25: 1115. doi: 10.3390/ijms25021115.

Kant, P., Kant, S., Gordon, M., Shaked, R., and Barak, S. (2007). STRESS RESPONSE SUPPRESSOR1 and STRESS RESPONSE SUPPRESSOR2, two DEAD-box RNA helicases that attenuate Arabidopsis responses to multiple abiotic stresses. Plant Physiol. 145: 814-830. doi: 10.1104/pp.107.099895.

Keller, M., Schleiff, E., and Simm, S. (2020). miRNAs involved in transcriptome remodeling during pollen development and heat stress response in Solanum lycopersicum. Sci. Rep. 10: 10694. doi: 10.1038/s41598-020-67833-6.

Khan, G.A., Deforges, J., Reis, R.S., Hsieh, Y.F., Montpetit, J., Antosz, W., Santuari, L., Hardtke, C.S., Grasser, K.D., and Poirier, Y. (2020). The transcription and export complex THO/TREX contributes to transcription termination in plants. PLoS Genet. 16: e1008732. doi: 10.1371/journal.pgen.

Kesten, C., Menna, A., and Sánchez-Rodríguez, C. (2017). Regulation of cellulose synthesis in response to stress. Curr. Opin. Plant Biol. 40:106-113. doi: 10.1016/j.pbi.2017.08.010. Knopf, R.R., and Adam, Z. (2012). Rhomboid proteases in plants - still in square one? Physiol. Plant. 145:41-51. doi: 10.1111/j.1399-3054.2011.01532.x.

Kovacs, D., Rakacs, M., Agoston, B., Lenkey, K., Semrad, K., Schroeder, R., and Tompa, P. (2009). Janus chaperones: assistance of both RNA- and protein-folding by ribosomal proteins. FEBS Lett. 583: 88-92. doi: 10.1016/j.febslet.2008.11.049.

Krämer, M., Dörfer, E., Hickl, D., Bellin, L., Scherer, V., and Möhlmann, T. (2022). Cytidine triphosphate synthase four from Arabidopsis thaliana attenuates drought stress effects. Front. Plant Sci. 13: 842156. doi: 10.3389/fpls.2022.842156.

Krtková, J., Benáková, M., and Schwarzerová, K. (2016). Multifunctional microtubule-associated proteins in plants. Front. Plant Sci. 7: 474. doi: 10.3389/fpls.2016.00474.

Lavell, A., Froehlich, J.E., Baylis, O., Rotondo, A.D., and Benning, C. (2019). A predicted plastid rhomboid protease affects phosphatidic acid metabolism in Arabidopsis thaliana. Plant J. 99: 978- 987. doi: 10.1111/tpj.14377.

Li, J., Kong, D., Song, T., Hu, Z., Li, Q., Xiao, B., Kessler, F., Zhang, Z., and Xie, G. (2023). OsFBN7-OsKAS I module promotes formation of plastoglobules clusters in rice chloroplasts. New Phytol. 239:1771-1789. doi: 10.1111/nph.19081.

Li, X.L., Hu, Y.X., Yang, X., Yu, X.D., and Li Q.L. (2014). A novel zinc-finger HIT protein with an additional PAPA-1-like region from Suaeda liaotungensis K. enhanced transgenic Arabidopsis drought and salt stresses tolerance. Mol. Biotechnol. 56: 1089-1099. doi: 10.1007/s12033-014-9789-2.

Li, Z., Shi, Y., Xiao, X., Song, J., Li, P., Gong, J., Zhang, H., Gong, W., Liu, A., Peng, R., et al. (2023). Genome-wide characterization of trichome birefringence-like genes provides insights into fiber yield improvement. Front. Plant Sci. 14: 1127760. doi: 10.3389/fpls.2023.1127760.

Liu, Z.W., Simmons, C.H., and Zhong, X. (2022). Linking transcriptional silencing with chromatin remodeling, folding, and positioning in the nucleus. Curr. Opin. Plant Biol. 69: 102261. doi: 10.1016/j.pbi.2022.102261.

Ma, J., Wang, D., She, J., Li, J., Zhu, J.K., and She, Y.M. (2016). Endoplasmic reticulum-associated N-glycan degradation of cold-upregulated glycoproteins in response to chilling stress in Arabidopsis. New Phytol. 212:282-96. doi: 10.1111/nph.14014.

Ma, L., Cheng, K., Li, J., Deng, Z., Zhang, C., and Zhu, H. (2021). Roles of plant glycine-rich RNA-binding proteins in development and stress responses. Int. J. Mol. Sci. 22: 5849. doi: 10.3390/ijms22115849.

Mahajan, A., Yuan, C., Lee, H., Chen, E.S., Wu, P.Y., and Tsai, M.D. (2008). Structure and function of the phosphothreonine-specific FHA domain. Sci. Signal. 1: re12. doi: 10.1126/scisignal. Mehdi, S.M.M., Szczesniak, M.W., and Ludwików, A. (2023). The Bro1-like domain-containing protein, AtBro1, modulates growth and abiotic stress responses in Arabidopsis. Front. Plant Sci. 14: 1157435. doi: 10.3389/fpls.2023.1157435.

Mishra, D., Shekhar, S., Chakraborty, S., and Chakraborty, N. (2018). Carboxylate clamp tetratricopeptide repeat (TPR) domain containing Hsp90 cochaperones in Triticeace: An insight into structural and functional diversification. Environ. Exp. Bot. 155: 31-44. https://doi.org/10.1016/j.envexpbot.2018.06.020.

Miwa, H., Kinoshita, A., Fukuda, H., and Sawa, S. (2009). Plant meristems: CLAVATA3/ESR-related signaling in the shoot apical meristem and the root apical meristem. J. Plant Res. 122: 31-39. doi: 10.1007/s10265-008-0207-3. Mu, C., Chen, N., Li, X., Jia, P., Wang, Z., and Liu, H. (2010). F-box protein arabidillo-1 promotes lateral root development by depressing the functioning of GA3 in Arabidopsis. J. Plant Biol. 53: 374–380. https://doi.org/10.1007/s12374-010-9125-8.

Muñoz-Díaz, E., and Sáez-Vásquez, J. (2022). Nuclear dynamics: Formation of bodies and trafficking in plant nuclei. Front. Plant Sci. 23: 13:984163. doi: 10.3389/fpls.2022.984163.

Nobusawa, T., Kamei, M., Ueda, H., Matsushima, N., Yamatani, H., and Kusaba, M. (2021). Highly pleiotropic functions of CYP78As and AMP1 are regulated in non-cell-autonomous/organ-specific manners. Plant Physiol. 186: 767-781. doi: 10.1093/plphys/kiab067.

Ohira, T., and Suzuki, T. (2024). Transfer RNA modifications and cellular thermotolerance. Mol. Cell 84: 94-106. doi: 10.1016/j.molcel.2023.11.041.

Osakabe, Y., Mizuno, S., Tanaka, H., Maruyama, K., Osakabe, K., Todaka, D., Fujita, Y., Kobayashi, M., Shinozaki, K., and Yamaguchi-Shinozaki, K. (2010). Overproduction of the membrane-bound receptor-like protein kinase 1, RPK1, enhances abiotic stress tolerance in Arabidopsis. J. Biol. Chem. 285: 9190-9201. doi: 10.1074/jbc.M109.051938.

Ok, S.H., Jeong, H.J., Bae, J.M., Shin, J.S., Luan, S., and Kim, K.N. (2005). Novel CIPK1-associated proteins in Arabidopsis contain an evolutionarily conserved C-terminal region that mediates nuclear localization. Plant Physiol. 139: 138-150. doi: 10.1104/pp.105.065649.

Ou, Y., Tao, B., Wu, Y., Cai, Z., Li, H., Li, M., He, K., Gou, X., and Li, J. (2022). Essential roles of SERKs in the ROOT MERISTEM GROWTH FACTOR-mediated signaling pathway. Plant Physiol. 189 :165-177. doi: 10.1093/plphys/kiac036.

Pareek, A., Mishra, D., Rathi, D., Verma, J.K., Chakraborty, S., and Chakraborty, N. (2021). The small heat shock proteins, chaperonin 10, in plants: an evolutionary view and emerging functional diversity. Environ. Exp. Bot. 182: 104323. doi: 10.1111/ppl.12080.

Periappuram, C., Steinhauer, L., Barton, D.L., Taylor, D.C., Chatson, B., and Zou, J. (2000). The plastidic phosphoglucomutase from Arabidopsis. A reversible enzyme reaction with an important role in metabolic control. Plant Physiol. 122: 1193-1199. doi: 10.1104/pp.122.4.1193.

Perrella, G., Bäurle, I., and van Zanten, M. (2022). Epigenetic regulation of thermomorphogenesis and heat stress tolerance. New Phytol. 234: 1144-1160. doi: 10.1111/nph.17970.

Praat, M., De Smet, I., van Zanten, M. (2021) Protein kinase and phosphatase control of plant temperature responses. J. Exp. Bot. 72: 7459-7473. doi: 10.1093/jxb/erab345.

Qiao, D., Zhang, Y., Xiong, X., Li, M., Cai, K., Luo, H., and Zeng, B. (2020). Transcriptome analysis on responses of orchardgrass (Dactylis glomerata L.) leaves to a short-term flooding. Hereditas 157: 20. doi: 10.1186/s41065-020-00134-0.

Qu, L., Liu, M., Zheng, L., Wang, X., and Xue, H. (2023). Data-independent acquisition-based global phosphoproteomics reveal the diverse roles of casein kinase 1 in plant development. Sci. Bull. (Beijing) 68: 2077-2093. doi: 10.1016/j.scib.2023.08.017.

Qu, L., Sun, M., Li, X., He, R., Zhong, M., Luo, D., Liu, X., and Zhao, X. (2020). The Arabidopsis F-box protein FOF2 regulates ABA-mediated seed germination and drought tolerance. Plant Sci. 301: 110643. doi: 10.1016/j.plantsci.2020.110643.

Ramakrishnan, M., Rajan, K.S., Mullasseri, S., Palakkal, S., Kalpana, K., Sharma, A., Zhou, M., Vinod, K.K., Ramasamy, S., and Wei, Q. (2022). The plant epitranscriptome: revisiting pseudouridine and 2'-O-methyl RNA modifications. Plant Biotechnol. J. 20: 1241-1256. doi: 10.1111/pbi.13829.
Ren, Y., Wang, Y., Zhang, Y., Pan, T., Duan, E., Bao, X., Zhu, J., Teng, X., Zhangm, P., Gum C., et al. (2022). Endomembrane-mediated storage protein trafficking in plants: Golgi-dependent or Golgi-independent? FEBS Lett. 596: 2215-2230. doi: 10.1002/1873-3468.14374.
Robles, P., and Quesada, V. (2022). Unveiling the functions of plastid ribosomal proteins in plant development and abiotic stress tolerance. Plant Physiol. Biochem. 189: 35-45. doi: 10.1016/j.plaphy.2022.07.029.
Saravanan, R.S., Slabaugh, E., Singh, V.R., Lapidus, L.J., Haas, T., and Brandizzi, F. (2009). The targeting of the oxysterol-binding protein ORP3a to the endoplasmic reticulum relies on the plant VAP33 homolog PVA12. Plant J. 58: 817-830. doi: 10.1111/j.1365-313X.2009.03815.x.
Schalk, C., Drevensek, S., Kramdi, A., Kassam, M., Ahmed, I., Cognat, V., Graindorge, S., Bergdoll, M., Baumberger, N., Heintz, D., et al. (2016). DNA DAMAGE BINDING PROTEIN2 Shapes the DNA Methylation Landscape. Plant Cell 28: 2043-2059. doi: 10.1105/tpc.16.00474.
Shao, J., Huang, K., Batool, M., Idrees, F., Afzal, R., Haroon, M., Noushahi, H.A., Wu, W., Hu, Q., Lu, X., et al. (2022). Versatile roles of polyamines in improving abiotic stress tolerance of plants. Front. Plant Sci. 13: 1003155. doi: 10.3389/fpls.2022.1003155.
Shao, C., Wu, Q., Qiu, J., Jin, S., Zhang, B., Qian, J., Chen, M., and Meng, Y. (2013). Identification of novel microRNA-like-coding sites on the long-stem microRNA precursors in Arabidopsis. Gene 527: 477-483. doi: 10.1016/j.gene.2013.06.070.
Shao, Y.J., Zhu, Q.Y., Yao, Z.W., and Liu, J.X. (2021). Phosphoproteomic analysis of thermomorphogenic responses in Arabidopsis. Front. Plant Sci. 12: 753148. doi: 10.3389/fpls.2021.753148. Sharma, M., and Pandey, G.K. (2016). Expansion and function of repeat domain proteins during stress and development in plants. Front. Plant Sci. 6: 1218. doi: 10.3389/fpls.2015.01218.
Singh, R.K., Prasad, A., Maurya, J., and Prasad, M. (2022). Regulation of small RNA-mediated high temperature stress responses in crop plants. Plant Cell Rep. 41:765-773. doi: 10.1007/s00299- 021-02745-x.
Sun, A., Yu, B., Zhang, Q., Peng, Y., Yang, J., Sun, Y., Qin, P., Jia, T., Smeekens, S., and Teng, S. (2020). MYC2-Activated TRICHOME BIREFRINGENCE-LIKE37 acetylates cell walls and enhances herbivore resistance. Plant Physiol. 184: 1083-1096. doi: 10.1104/pp.20.00683.
Trösch, R., Ries, F., Westrich, L.D., Gao, Y., Herkt, C., Hoppstädter, J., Heck-Roth, J., Mustas, M., Scheuring, D., Choquet, Y., et al. (2022). Fast and global reorganization of the chloroplast protein biogenesis network during heat acclimation. Plant Cell 34: 1075-1099. doi: 10.1093/plcell/koab317.
Verhertbruggen Y, Yin L, Oikawa A, and Scheller, H.V. (2011). Mannan synthase activity in the CSLD family. Plant Signal. Behav. 6: 1620-1623. doi: 10.4161/psb.6.10.17989. Wang, M.B., and Dennis, E.S. (2009). SPT5-like, a new component in plant RdDM. EMBO Rep. 10: 573-575. doi: 10.1038/embor.2009.101.
Wang, C., Aubé, F., Quadrado, M., Dargel-Graffin, C., and Mireau, H. (2018). Three new pentatricopeptide repeat proteins facilitate the splicing of mitochondrial transcripts and complex I biogenesis in Arabidopsis. J. Exp. Bot. 69: 5131-5140. doi: 10.1093/jxb/ery275.
Wang, L., Sadeghnezhad, E., Guan, P., and Gong, P. (2021). Review: Microtubules monitor calcium and reactive oxygen species signatures in signal transduction. Plant Sci. 304: 110589. doi: 10.1016/j.plantsci.2020.110589.
Wang, L.C., Tsai, M.C., Chang, K.Y., Fan, Y.S., Yeh, C.H., and Wu, S.J. (2011). Involvement of the Arabidopsis HIT1/AtVPS53 tethering protein homologue in the acclimation of the plasma membrane to heat stress. J, Exp. Bot. 62: 3609-3620. doi: 10.1093/jxb/err060.
Weber, C., Nover, L., and Fauth, M. (2008). Plant stress granules and mRNA processing bodies are distinct from heat stress granules. Plant J. 56: 517-530. doi: 10.1111/j.1365-313X.2008.03623.x. Wei, K., and Han, P. (2016). Pentatricopeptide repeat proteins in maize. Mol. Breeding 36: 170. https://doi.org/10.1007/s11032-016-0596-2.
Wei, M., Duan, P., Zhao, S., Gou, B., Wang, Y., Yang, N., Ma, Y., Ma, Z., Zhang, G., and Wei B. (2023). Genome-wide identification of RUB activating enzyme and conjugating enzyme gene families and their expression analysis under abiotic stresses in Capsicum annuum. Protoplasma 260: 821-837. doi: 10.1007/s00709-022-01816-4.
Wu, J., Okada, T., Fukushima, T., Tsudzuki, T., Sugiura, M., and Yukawa, Y. (2012). A novel hypoxic stress-responsive long non-coding RNA transcribed by RNA polymerase III in Arabidopsis. RNA Biol. 9: 302-313. doi: 10.4161/rna.19101.
Xing, D., Zhao, H., Xu, R., and Li, Q.Q. (2008). Arabidopsis PCFS4, a homologue of yeast polyadenylation factor Pcf11p, regulates FCA alternative processing and promotes flowering time. Plant J. 54: 899-910. doi: 10.1111/j.1365-313X.2008.03455.x.
Xiong, F., Liu, H.H., Duan, C.Y., Zhang, B.K., Wei, G., Zhang, Y., and Li, S. (2019). Arabidopsis JANUS regulates embryonic pattern formation through Pol II-mediated transcription of WOX2 and PIN7. iScience 19: 1179-1188. doi: 10.1016/j.isci.2019.09.004.
Yan, Y., Gan, J., Tao, Y., Okita, T.W., and Tian, L. (2022). RNA-binding proteins: The key modulator in stress granule formation and abiotic stress response. Front. Plant Sci. 13: 882596. doi: 10.3389/fpls.2022.882596.
Yang, L., Peng, X., and Sun, M.X. (2011). AtNG1 encodes a protein that is required for seed germination. Plant Sci. 181: 457-464. doi: 10.1016/j.plantsci.2011.07.011.
Yang, S., Zhang, F., Wang, Y., Xue, H., Jiang, Q., Shi, J., Dai, H., Zhang, Z., Li, L., He, P., et al. (2020). MdHAL3, a 4'-phosphopantothenoylcysteine decarboxylase, is involved in the salt tolerance of autotetraploid apple. Plant Cell Rep. 39: 1479-1491. doi: 10.1007/s00299-020-02576-2.
Yu, T.Y., Xu, C.X., Li, W.J., and Wang, B. (2022). Peptides/ receptors signaling during plant fertilization. Front. Plant Sci. 13: 1090836. doi: 10.3389/fpls.2022.1090836.
Zagorchev, L., Seal, C.E., Kranner, I., and Odjakova, M. (2013). A central role for thiols in plant tolerance to abiotic stress. Int. J. Mol. Sci. 14: 7405-7432. doi: 10.3390/ijms14047405.
Zeng, Y., Pan, Z., Wang, L., Ding, Y., Xu, Q., Xiao, S., and Deng, X. (2014). Phosphoproteomic analysis of chromoplasts from sweet orange during fruit ripening. Physiol. Plant. 150: 252-270. doi: 10.1111/ppl.12080.
Zhang, H., Rundle, C., Winter, N., Miricescu, A., Mooney, B.C., Bachmair, A., Graciet, E., and Theodoulou, F.L. (2024). BIG enhances Arg/N-degron pathway-mediated protein degradation to regulate Arabidopsis hypoxia responses and suberin deposition. Plant Cell Apr 12:koae117. doi: 10.1093/plcell/koae117.
Zhang, J., Yuan, H., Yang, Y., Fish, T., Lyi, S.M., Thannhauser, T.W., Zhang, L., and Li, L. (2016). Plastid ribosomal protein S5 is involved in photosynthesis, plant development, and cold stress tolerance in Arabidopsis. J. Exp. Bot. 67: 2731-2744. doi: 10.1093/jxb/erw106.
Zhao, Y., Qin, Q., Chen, L., Long, Y., Song, N., Jiang, H., and Si, W. (2022) Characterization and phylogenetic analysis of multiple C2 domain and transmembrane region proteins in maize. BMC Plant Biol. 22: 388. doi: 10.1186/s12870-022-03771-x.
Zhou, H., Duan, H., Liu, Y., Sun, X., Zhao, J., and Lin, H. (2019). Patellin protein family functions in plant development and stress response. J. Plant Physiol. 234-235: 94-97. doi: 10.1016/j.jplph.2019.01.012.
Zhou, X., Li, Y., Wang, J., Zhao, Y., Wang, H., Han, Y., and Lin, X. (2024). Genome-wide identification of U-box gene family and expression analysis in response to saline-alkali stress in foxtail millet (Setaria italica L. Beauv). Front Genet. 15: 1356807. doi: 10.3389/fgene.2024.1356807.
Zhou, H., Liu, Y., Liang, Y., Zhou, D., Li, S., Lin, S., Dong, H., and Huang, L. The function of histone lysine methylation related SET domain group proteins in plants. Protein Sci. 29: 1120-1137. doi: 10.1002/pro.3849
Zhu, L., Chu, L.C., Liang, Y., Zhang, X.Q., Chen, L.Q., and Ye, D. (2018). The Arabidopsis CrRLK1L protein kinases BUPS1 and BUPS2 are required for normal growth of pollen tubes in the pistil. Plant J. 95: 474-486. doi: 10.1111/tpj.13963.
Zuo, Z.F., He, W., Li, J., Mo, B., and Liu, L. (2021). Small RNAs: The essential regulators in plant thermotolerance. Front. Plant Sci. 12: 726762. doi: 10.3389/fpls.2021.726762.

Table S5. Transcriptional responses during recovery (1 and 3 days-post-HS) in *AhHAB4-PAI-1* and *Ah2880* overexpressing *A. thaliana* plants.

***Ah2880*: group 1 and 2**	***AhHAB4-PAI-1*: group 4**
**Category 1: Group-defining transcription factors**	
MYB2 (AT2G47190); *ERF113* (AT1G43160); *NAC19* (AT1G52890); *DREB19* (AT2G38340); *WRKY57* (AT1G69310); *BROMODOMAIN* TF (AT3G15500; Zheng et al., 2025); *ABA-RESPONSIVE ELEMENT BINDING PROTEIN 2;* (AT3G19290); *ERF58* (AT1G22190); *ERF59* (AT1G78080); *ERF070* (AT1G71130); *ERF107* (AT5G61590); *NIN Like Protein* (AT4G24020); *WRKY 33* (AT2G38470); *RELATED TO AP2.1*, *RAP2.2* (AT1G46768); *MYELIN TF-like* proteins (AT2G47950, AT3G62990); *MYB84* (AT3G49690); AT-rich zinc-binding *PLATZ* TF (AT1G76590; (Li et al. 2015; Hu et al. 2023; Zhang et al. 2023).	MYB76 (AT5G07700); YB95 (AT1G74430); MYB29 (AT5G07690); MYB60 (AT1G08810); NUCLEAR FACTOR Y SUBUNIT B3 (AT4G14540); ERF016 (AT5G21960).
**Associated transcription factors/ transcription regulators**	
WITH NO LYSINE (K) KINASE (AT5G58350), involved in circadian rhythm, flowering time, and stress responses (Uchida et al. 2014); *NIGHT LIGHT-INDUCIBLE AND CLOCK-REGULATED 2*, agglutinin-like protein (AT3G54500);*PSEUDO-RESPONSE REGULATOR 7* (Yuan et al. 2021b); *SCARECROW-LIKE PROTEIN 3* (AT1G50420), a GRAS TF involved in gibberellin-mediated signaling (Waseem et al. 2022); *GRAS2* (AT1G07530), MYB84 (AT3G49690); DIV2, DIVARICATA2, R-R-type MYB TF CDF4 a DOF TF (AT2G34140) promotes leaf senescence and floral abscission (Xu et al. 2020); *DOF AFFECTING GERMINATION 1* (AT3G61850); *CYCLING DOF FACTOR 2* (AT5G39660). *bHLH163* (AT1G10585) and several other *bHLH* TF genes (AT4G25400, AT2G40200, AT5G51780, AT5G51790, AT5G51790); *HOMEODOMAIN-LIKE* and *DUPLICATED HOMEODOMAIN* TF genes (AT5G01380, AT3G10590); *ERF subfamily B-4* (AT2G33710); *ERF111* (AT5G64750); *WOUND INDUCED DEDIFFERENTIATION* 1, a DREB TF (AT1G78080); *ERF58 NAC03* (AT1G34180); *NAC19* (AT1G52890); *NAC48* (AT3G04420); *NAC55* (AT3G15500); *NAC75* (AT4G29230); *NAC80* (AT5G07680); *NAC84* (Xiong et al. 2025); *WRKY4* (AT1G13960); *WRKY15* (AT2G23320); *WRKY 28* (AT4G18170); *WRKY 33* (AT2G38470); *WRKY48* (AT5G49520); *WRKY61* (AT1G18860); two VQ motif-containing proteins (AT4G15120, AT2G22880), (Yuan et al. 2021a*); GLOBAL TRANSCRIPTION FACTOR GROUP E4*, (AT1G06230); *SMAD/FHA* domain-containing protein (AT3G07260); *TOPLESS* family of transcriptional co-repressors (AT5G27030 and AT3G15880); *ALFIN1-like* protein (AT1G14510; Jin et al. 2024); *SOD7* (AT1G69310; Zheng et al. 2023); *DP-E2F-like 2* (AT5G14960; Murray, 2004); *DEFECTIVE REGION OF POLLEN 1* (AT2G24260).	*bZIP34* (AT2G42380); *EMB3022/ ZP1*, *EMBRYO DEFECTIVE 3022* (AT4G17810; Riechmann 2023); *NIN-LIKE PROTEIN 2* (AT4G35270); *MYB28* (AT5G61420); *HOMOLOG OF BEE2 INTERACTING WITH IBH 1* (AT2G18300); *INDUCER OF CBF EXPRESSION 2* (AT1G12860; Li et al. 2025); *ERF34* (AT2G44940); *KIDARI/ PACLOBUTRAZOL RESISTANCE 6* (AT1G26945; Chen and Wang 2017); *GATA TF 20 P1R* bHLH TF (AT5G57780); *TANDEM ZINC FINGER 8* (AT5G12850); *GIBBERELLIC ACID INSENSITIVE* (AT1G14920); *ATBS1 INTERACTING FACTOR 4* (AT1G09250; Li et al. 2021); *PHD5*, a PHD finger-containing TF (AT5G61100); *ZAT4*, C2H2-type zinc finger TF (AT2G45120); *TARGET OF EARLY ACTIVATION 3* (AT5G67180; Jung et al. 2014); *ZINC FINGER PROTEIN 8* (AT2G41940).
**Category 2: Heat tress response-proteins including HSFs, HSPs and co-chaperones, protein modifications and others**	
RING/U-box superfamily protein genes (AT2G42360, AT3G10910, AT5G08139, AT1G76410 AT5G05530 AT5G37250 AT3G13430 AT5G41350 AT5G01450 AT3G19895 AT2G01275); RING- H2 finger protein *RHA1a* (AT4G11370; Han et al. 2022); similar to RING, zinc finger proteins (AT5G22920) known to control stomatal aperture; *RING FINGER E3 UBIQUITIN LIGASE* (AT4G28270; Gelová et al. 2021; Patnaik et al., 2024); *Plant U-box type E3 UBIQUITIN LIGASE* (AT5G01830); *E3 UBIQUITIN LIGASE* (AT5G49665) involved in root gravitropism; E3 ubiquitin-protein ligase *PRT1* (AT3G24800; Potuschak et al. 1998); ubiquitin-	*RING/U-box* superfamily protein gene (AT4G30400); *RING/ FYVE/ PHD zinc finger* superfamily protein gene (AT2G37950); *ENDOMEMBRANE PROTEIN 70 PROTEIN* (AT5G25100), part of the Golgi membrane complex (Vitale and Galili 2001).

specific proteas gene (AT3G11910); small ubiquitin-like modifier *SUMO E3 LIGASE* (AT5G60410), involved in the regulation of plant growth, drought responses and freezing tolerance. *VACUOLAR SORTING RECPTOR 7* (AT4G20110); part of the MAG2 ER complex (AT5G24350; Isono et al. 2021; Wang et al. 2020a, 2020b); *LIPID DROPLET AND ER-ASSOCIATED PROTEIN* (AT1G70680; Zhang et al. 2020); *Ypt/Rab-GUANOSINE TRIPHOSPHATASE* (AT4G29950), that regulators of protein transport via vesicle trafficking (Segev 2001); *VAMP72* (AT2G33110), an ER-localized SNARE protein, paralog of *LOG2* (AT5G19080); prenylated *RAB ACCEPTOR 1.B5* (AT5G01640), a possible escort protein for small GTPases during their movement through the endomembrane system (Figueroa et al. 2001); *CNIH3* (AT1G62880) an ER cargo receptor; *GOLGIN family A* (AT4G22320); *UDP-GALACTOSE TRANSPORTER 6* (AT3G59360; Reyes et al. 2006); *DNAJ* heat shock N-terminal domain-containing chaperone protein genes (AT2G05250, AT1G72070; AT3G47940); *HSP40/DnaJ PEPTIDE-BINDING* (AT1G11040); *HEAT STRESS TRANSCRIPTION FACTOR A4A* (AT4G18880; Andrási et al. 2019); *SMAX1- LIKE 6* (AT1G07200; Park et al. 2022). *AUTOPHAGY-RELATED 2* (AT3G19190; Thirumalaikumar et al. 2021; Zhang et al. 2021); yeast *AUTOPHAGY 18 F-LIKE* protein gene (AT5G54730); *ATG8-interacting protein 2* (AT4G00355), promotes the rapid vesiculation of the Golgi apparatus and the translocation of ATG8 onto single membrane organelles (Zheng et el. 2024); *TUMOR NECROSIS FACTOR RECEPTOR*-associated family protein gene (AT4G09770) that regulates autophagy via ubiquitination (Qi et al. 2017); *Paralog of LOG2* (AT5G19080), a ubiquitin ligase that regulates amino acid export; *CLATHRIN LIGHT CHAIN* protein gene (AT3G51890) recruited by ATG8 for Golgi reassembly (Zheng et al. 2024); EKC/KEOPS complex subunit *TPRKB-LIKE PROTEIN* (AT4G34412; Yang et al. 2023); *POLYPRENOL REDUCTASE*, involved in N-gylcosylation (AT2G16530).	
**Category 3: Regulation of HS responses involving DNA repair non-Coding RNAs, epigenetic regulation, DNA methylation, histone modification, chromatin remodeling, epigenetic memory and RNA alternative splicing**	
ESSENTIAL MEIOTIC ENDONUCLEASE 1B (AT2G22140; Modliszewski et al. 2018); MMZ3/UEV1C (AT2G36060; Zhao et al. 2011); polynucleotidyl transferase, ribonuclease H-like superfamily protein (AT3G25430), that maintains mRNA stability and/or translational efficiency (Arae et al., 2019); DNA mismatch repair *MUTS, type 2* protein gene (AT5G54090) that maintains genomic stability (Chirinos-Arias and Spampinato 2021); polyadenylate-binding protein (AT1G34140); *class I POLYA-BINDING PROTEIN* (AT1G71770; Zhou and Li 2023); *RNA-BINDING PROTEIN WITH A POLYADENYLATE-BINDING DOMAIN* (AT4G10610); pre-mRNA splicing *PRP18-INTERACTING FACTOR* (AT3G45950); *AMMECR1* (AT2G38710), possibly involved in tRNA 4-thiouridylation along with other proteins (Burroughs and Aravind 2014); *CHROMATIN REMODELLING 12* (AT3G06010), a SNF2/Brahma-type chromatin-remodeling protein (Haider et al. 2021); *SET DOMAIN CONTAINING* protein genes (AT4G15180, AT3G61740; Zhou et al. 2020); *HAF1*, a predicted histone acetyltransferase (AT1G32750; Hirakawa et al, 2023); ribosomal RNA small subunit *METHYLTRANSFERASE G* (AT5G57910; Ngoc et al. 2021); *0SGS3-LIKE, XH/XS DOMAIN-CONTAINING PROTEIN* (AT5G59390; Qin et al. 2009); *LUC7 N TERMINUS DOMAIN-CONTAINING PROTEIN* (AT5G51410; de Francisco Amorim et al. 2018); splicing factor 3A subunit (AT5G04860); histone-lysine N-methyltransferase *TRITHORAX*-	*DNA GLYCOSYLASE SUPERFAMILY PROTEIN* (AT5G44680), that recognizes and repairs small base lesions in DNA (Cecchini et al. 2022); *HISTONE 2B PROTEIN* (AT5G22880) that is mono-ubiquitinated to control transcriptional activation during stress conditions (Zarreen et al. 2022); *XRI1-like PROTEIN* (AT2G01990; Dean et al. 2009); *LOW PROTEIN*, ATP-dependent RNA helicase (AT1G15260) possibly needed to maintain rRNA homeostasis under high temperature (Pandey et al. 2020).

LIKE PROTEIN (AT5G40690); JMJ24 (AT1G09060), a nuclear-localized *JmjC DOMAIN CONTAINING PROTEIN* that demethylates the histone H3K9 site (Shang et al. 2021); *miR163* (AT1G66725) controlling the expression of stress responses, including heat (Kok et al. 2023); LOW protein, ATP-dependent RNA helicase (AT5G28610) that may maintain rRNA homeostasis under high temperatures (Pandey et al. 2020).	
**Category 4: Cell wall/membrane modifications and ROS-associated responses**	
GLUTATHIONE TRANSFERASES (AT5G62480, AT1G02920, AT2G02930, AT4G02520); *GLUTATHIONE PEROXIDASE* (AT2G31570); two *GLUTAREDOXINS*, including *GRXS13* (AT1G03850, AT3G28850); *PDS1* (AT1G06570); *L-ASCORBATE OXIDASE* (AT5G21105); *MAN3* (AT1G78830; Yu et al. 2021; Fortunato et al. 2023; Madhu et al. 2023); *L-GULONO-1,4-LACTONE OXIDASE* (AT2G46740); senescence-associated *Fe(II)/ascorbate oxidase* (AT1G17020); *TLD-DOMAIN CONTAINING NUCLEOLAR PROTEIN* (AT5G39590, AT4G39870; Colombatti et al. 2019; Ma et al. 2017); *METHIONINE SULFOXIDE REDUCTASE B3* (AT4G04800; Henriet et al., 2021); an auxin- and water-stress inducible *EPOXIDE HYDROLASE* (AT2G26740; Wang et al. 2020c); *PEROXIDASE* superfamily protein genes (AT2G37130, AT5G64110); *NUDIX HYDROLASE HOMOLOG 18* (AT1G14860; Paradiso et al. 2020); cytosolic *THIOREDOXINS* (AT1G19730, AT1G19730) acting either as disulfide reductases or as protein chaperones (Calderón et al. 2018); *β-glucosidase* involved in xyloglucan metabolism (AT5G20950); a bifunctional *ALPHA-L- ARABINOFURANOSIDASE/ β-D-XYLOSIDASE* (AT3G10740); xylose isomerase family protein (AT5G57655) and a secreted *β-D-XYLOSIDASE* (AT5G64570) that control cell wall extensibility by organizing xyloglucan-related cellulose- cellulose interactions (Ezquer et al. 2020); *XYLOSYLTRANSFERASE FAMILY 61* (AT3G57380), required for xyloglucan synthesis, the main hemicellulosic polysaccharides of primary cell walls (Cavalier et al. 2008); *WSD6* (AT3G49210; Kan et al. 2022); *SIP2*, a raffinose-specific alpha-galactosidase (AT3G57520) (Yan et al. 2022); *DEHISCENCE ZONE POLYGALACTURONASE 1* (AT3G57510), a polygalacturonase that releases cell wall-derived defense-inducing elicitors (Gallego-Giraldo et al. 2020); *PMEI9* pectin methyleseterase inhibitor (AT1G62770); an ER- localized *SPHINGOID LONG-CHAIN BASE-1-PHOSPHATE LYASE* (AT1G27980) involved in signal transduction pathways during dehydration stress that lead to programmed cell death (Lambour et al. 2022); Golgi membrane-localized *CELLULOSE SYNTHASE* (AT5G16910; Wang et al. 2023); lignin biosynthesis related enzymes cinnamyl alcohol dehydrogenase (AT4G34230), *4-COUMARATE: COA LIGASE* (4CL) (AT1G51680), *4-COUMARATE COENZYME A LIGASE* (AT3G21230), S-adenosyl-L-methionine: transcaffeoyl coenzyme A, 3-O-methyltransferase (AT1G67980), *CAFFEOYL SHIKIMATE ESTERASE* (AT1G52760), *P-COUMARYL ALCOHOL EXPORTER* (AT3G16340), *PINORESINOL REDUCTASE* (AT4G13660) and an unknown function protein involved in formation of the casparian strip (AT2G28670); an homolog of *KPP* (*KINASE PARTNER PROTEIN*) in tomato (AT5G02010) involved in the formation of secondary cell wall pits (Denzler 2021); *FASCICLIN-LIKE ARABINOGALACTAN PROTEIN* (AT1G15190), cell-wall related protein that participates in stress-related changes in secondary cell wall formation (He et al. 2019); plant *FLOTILLIN* (AT5G64870), a plasma membrane protein found in membrane nanodomains and	*PLECKSTRIN HOMOLOGY (PH) DOMAIN* superfamily protein gene (AT1G77730), a mediator of protein-membrane interactions through phosphatidylinositol phosphate (Naughton et al. 2018); *EXTENSIN PROLINE-RICH 1* (AT1G03820); *MITOGEN-ACTIVATED PROTEIN KINASE 8* (AT1G18150); *PECTIN LYASE-LIKE* superfamily protein genes (AT5G48900, AT5G55720); *GALACTOSE OXIDASE-LIKE 5* (AT1G14430; Šola et al. 2021); *COTTON FIBER* protein gene (AT5G06790); *EXPANSIN A6* (AT2G28950), that promotes cell enlargement *via* cell wall loosening (Bordoloi et al., 2021); pectin methylesterase inhibitor, *PMEI-PME51* (AT5G09760) and *PECTIN METHYLESTERASE 44* (AT4G33220;Coculo and Lionetti 2022); *POLLEN OLE E1* allergen and extensin family protein genes (AT5G41050, AT2G20515), pollen allergens possibly involved in stress responses through cell wall modifications (Chen et al., 2016); O-fucosyltransferase family protein (AT1G62330), possibly involved in development, cell expansion, adhesion, signaling and energy metabolism (Soto et al., 2019); *VITELLOGENIN-LIKE* protein genes (AT5G49100, AT3G06868); pectate lyase, *PLL19* (AT4G24780; Palusa et al. 2007).

endosomes (Daněk et al., 2016); *MYOTUBULARIN 1* (AT3G10550; Ding et al. 2012); *ALA-INTERACTING SUBUNIT 5* (AT1G79450), P4 ATPases, or lipid flippases that actively transport lipids to biological membranes in the secretory pathway that modulate their biological properties (López-Marqués et al. 2021); *Sec14p-like* phosphatidylinositol transfer family protein (AT1G75370), confers drought stress tolerance by altering membrane lipid composition and links specific lipid molecular species to enhance heat tolerance (Zhang et al. 2019).	
**Category 5: Transport, Carbon/ nitrogen metabolism and secondary metabolism**	
Belongs to a family of ER-localized plant aquaporins (AT5G18290), like *PIP1-1*, *PIP2-7*, *TIP2-1* (Qi et al. 2021); nodulin *MtN21-like* transporters (AT1G09380, AT3G53210, AT4G28040); nitrate transporter *NRT1.8* (AT4G21680); *SLAH2* (AT4G27970), a nitrate-specific anion channel; *NIN Like Protein 7*, *NLP7*, (AT4G24020; Sámano et al. 2024); *PEPTIDE TRANSPORTER 1* (AT3G54140; Diyang et al. 2023); *CATIONIC AMINO ACID TRANSPORTERS* subfamily of amino acid polyamine choline transporters (AT5G04770, AT1G58030; Islam et al., 2024); organic *CATION/CARNITINE TRANSPORTER 6* (AT1G16370); plasma-membrane-localized *CHOLINE TRANSPORTER* family protein gene (AT5G13760); *ALANINE AMINOTRANSFERASE 2,* (AT1G72330; Kishorekumar et al. 2020); *PUP1* (AT4G18195) and *PURINE PERMEASE 18* (AT1G57990), purine and cytokinin transporters; *ALANINE: GLYOXYLATE AMINOTRANSFERASE 3* (AT2G38400), found to be sensitive to N levels in roots of poplar trees (Li et al. 2023). *PLASMA MEMBRANE PROTON ATPASE 2* (AT4G30190); cation-transporting ATPase, *KAT3* (AT4G32650), a Shaker family of voltage-gated potassium channel subunits; *GMP-ACTIVATED NON-SELECTIVE CATION CHANNEL* (AT2G23980; Niu et al. 2020); *CATION-CHLORIDE CO-TRANSPORTER 1* (AT1G30450); *CYSTEINE-RICH PROTEIN* genes (AT1G05340, AT1G05340); *CYSTEINE/HISTIDINE-RICH* protein genes (AT4G01740, AT3G45530, AT4G13130, AT5G59930, AT4G11470); *CYSTEINE/ HISTIDINE-RICH C1 DOMAIN* family protein genes (AT3G45530, AT4G13130, AT5G59930, AT4G11470), *CYSTEINE-RICH RECEPTOR-LIKE PROTEIN KINASE 17* (AT4G23250), *CYSTEINE-RICH RECEPTOR-LIKE PROTEIN KINASE* genes (AT1G70530, AT4G23270, AT4G11530; Zeiner et al. 2023); *MAJOR FACILITATOR* superfamily protein gene (AT1G72120); multidrug and toxic compound extrusion *MATE* family genes (AT3G23560, AT3G26590); *MULTIDRUG-RESISTANCE-RELATED* protein subfamily gene (AT3G21250); *GDP-MANNOSE TRANSPORTER* (AT1G07290); *SUCROSE TRANSPORTER 4* (AT1G09960), a low affinity sucrose transporter across phloem sieve elements; *SFP1* sugar-porter family protein induced during leaf senescence (AT5G27350); sucrose-proton symporter (AT1G71880); SULFATE TRANSPORTER 3 (AT3G15990; Sehar et al. 2022). *RNA LIGASE/CYCLIC NUCLEOTIDE PHOSPHODIESTERASE* family protein gene (AT4G18940) may regulate P levels in plant cells; *MITOCHONDRIAL PHOSPHATE TRANSPORTER* (AT3G48850), regulates alternative Pi-dependent glycolysis and mitochondrial respiration during salt stress and drought (Bechtaoui et al. 2021). *SUCROSE SYNTHASE 1* (AT5G20830); *TREHALOSE-6-PHOSPHATE SYNTHASE 8* (AT1G70290; Wei et al. 2022; Reichelt et al. 2023); *TREHALASE*, member of glycoside hydrolase family 37 (AT4G24040; Luo et al., 2021); *GCK DOMAIN-CONTAINING PROTEIN* gene (AT5G57640) or glucokinase, also proposed	*EARLY NODULIN-LIKE PROTEIN 8* (AT1G64640); *NODULIN MTN21-LIKE* transporter family protein genes (AT2G37460, AT3G28070, AT1G11450); *NITRATE TRANSPORTER 1.11* (AT1G52190) expressed in the plasma membrane and in the phloem to control nitrate redistribution to young leaves; *AMMONIUM TRANSPORTER 1* (AT4G28700); *HEAVY METAL ATPASE 2* that also functions as a zinc transporter (AT4G30110); *SWEET13* sucrose efflux transporter (AT5G50800) that together with SWEET14, mediate cellular gibberellin (GA) uptake to modulate the GA-related development (Kanno et al. 2016). *RmlC-like CUPIN* protein genes (AT4G14710, AT3G56820; Khan et al. 2022); *ISOPROPYLMALATE ISOMERASE 1* (AT3G58990), involved in the Met chain elongation pathway and leucine biosynthesis (Chen et al., 2021); *CYTOCHROME P450, FAMILY 81* (AT4G37400), hydroxylates the glucosinolate indole ring during the 4-methoxy-indol-3-yl-methyl and 1- methoxy-indol-3-yl-methyl glucosinolate biosynthetic pathway (Pfalz et al. 2011); *FLAVIN-MONOOXYGENASE GLUCOSINOLATE S-OXYGENASE 3* (AT1G62560), catalyzes the conversion of methylthioalkyl glucosinolates to methylsulfinylalkyl glucosinolates; *PHENYL ALANINE AMMONIA-LYASE 3* (AT5G04230); *ANTHRANILATE SYNTHASE 2* (AT2G29690; Balfagón et al. 2022).

to act as a gene regulator and/or controller of autophagy or programmed cell death (Rodríguez-Saavedra et al. 2021); TRANSALDOLASE (AT5G13420) that contributes to ROS homeostasis in response to glucose during early seedling growth; *BASIC LEUCINE ZIPPER 63* (AT5G28770), controlling circadian phase changes in response to energy starvation (Viana et al., 2021). *RmlC-like CUPIN* protein genes (AT4G14710, AT3G56820), members of a seed storage protein superfamily (Khan et al. 2022); *ASPARAGINASE* (AT3G16150) degrades L- asparagine to L-aspartic acid and ammonia; *GLUTAMATE SYNTHETASE* (AT1G66200; Balfagón et al. 2022; Lei et al. 2022); cytoplasmic *NUCLEOSIDE HYDROLASE* (AT2G36310) and *XANTHINE DEHYDROGENASE* (AT4G34890) involved in purine and/or pyrimidine catabolism; *COFACTOR OF NITRATE REDUCTASE AND XANTHINE DEHYDROGENASE 2* (AT2G31955), participates in NR and NO biosynthesis; *MULTIFUNCTIONAL PROTEIN 2* (AT3G06860) acting in peroxisomal fatty acid beta oxidation; may promote fatty acid-fueled respiration for heat- induced stomatal opening in the light (Korte et al., 2023); *ACYL-COA OXIDASE* with specificity for medium chain fatty acids (AT1G06290).<br>*CHOLINE SYNTHASE* (AT4G09760; Allakhverdiev et al., 2008); *SABATH METHYLTRANSFERASE* (AT5G38780), catalyzes the methylation of diverse plant metabolites, including phytohormones (Wang et al. 2024); *UDP-glycosyltransferase* superfamily protein genes (AT3G21790, AT3G46680) that catalyze glycosylation modifications that are important for secondary metabolite production and (a)biotic stress resistance (Chen et al. 2024); *ALDO-KETO REDUCTASE FAMILY 4 MEMBER C8* (AT2G37760) stress-responsive *NADPH-DEPENDENT ALDO-KETO REDUCTASE* that recognizes aliphatic and aromatic aldehydes and steroids; *TERPENOID SYNTHASE 12* (AT4G13280), catalyzes the wound-induced conversion of farnesyl diphosphate to (Z)-gamma-bisabolene, E-nerolidol and alpha-bisabolol; *DELTA1-PYRROLINE-5-CARBOXYLATE SYNTHASE* (AT2G39800), catalyzes the rate- limiting enzyme in proline biosynthesis; Trimeric *LpxA-like ACYLTRANSFERASE* enzyme (AT4G18300) acylates oxygen- and nitrogen-containing substrates to produce ester and amide functional groups in secondary metabolites (Fu et al. 2008).	
**Category 6: Ribosomal, chloroplast- and mitochondria-associated proteins**	
CLP PROTEASE regulatory subunit CLPX (AT5G53350; Llamas et al. 2017); *FBN11* (AT5G53450), lipid-binding plastid lipid-associated proteins required for plant growth, plastid stability and stress responses (Sun et al. 2022).	*COLD– REGULATED 414 THYLAKOID MEMBRANE 1* **(**AT1G29395; Tian et al. 2013); chloroplast localized and ATP-dependent *LON DOMAIN-CONTAINING PROTEIN 1* (AT1G19740; Shin et al. 2020); *PHOTOSYSTEM II MANGANESE-STABILIZING* protein family gene (*PsbO*) (AT4G37230).
**Category 7: Growth, development and phytohormone-related events**	
NHL6 (*NDR1/HIN1-like 6*) (AT1G65690; Amato et al. 2025); HVA22 (AT5G62490; Zhang et al. 2023); *COPPER AMINE OXIDASE1* (AT1G62810; Wimalasekera et al. 2011); ABA-IMPORTING TRANSPORTER 1, AIT1 (AT1G69850; Kanno et al. 2012); *DWARF 14* (AT3G03990; Chi et al. 2021; Kapoor et al. 2024); *CDL1* (AT5G02800; Rehman et al. 2022); two *HSD1* proteins genes (At5g50600 and At5g50700; Li et al., 2007); *CYP94B3* (AT3G48520; Li et al. 2022); *ENHANCED GRAVITROPISM 2* (AT2G45700; Kirschner et al. 2021); *BRISTLED 1* (AT5G65090) controls root hair morphogenesis and tip growth; *ROOT MERISTEM GROWTH FACTOR* (AT3G02240; Singh et al. 2024); *ILR1* (AT3G02875; Wu et al.	*SAP30 FUNCTION-RELATED 2* (AT1G19330); *SOUL HEME-BINDING* family protein gene (AT1G78460; Goodfellow et al. 2021); DP-E2F-like 1 (AT3G48160) *INFLORESCENCE MERISTEM RECEPTOR-LIKE KINASE 2* (AT3G51740) leucine-repeat receptor kinase expressed in inflorescence meristems; *ETTIN PROTEIN* (AT2G33860) homologous to DNA binding proteins that recognize auxin response elements to regulate flowering-related processes (Sessions et al., 1997); *SAUR-like AUXIN-RESPONSIVE PROTEIN* family (AT4G13790); *RAVEN* (AT2G02070) regulatory protein that controls root tissue patterning that together with *BLUEJAY*, *JACKDAW*, *SCARECROW* and *SHORT- ROOT TILLER ANGLE*

2019); *GRETCHEN HAGEN 3.15* (AT5G13370; Luo et al. 2023); *ARABIDOPSIS P-GLYCOPROTEIN 4* (AT2G47000) an auxin efflux transmembrane transporter; *TOM1-LIKE 5* (AT5G63640; Roach et al. 2021); *DORMANCY/AUXIN ASSOCIATED* family protein gene (AT1G56220). *SECRET AGENT* (AT3G04240) a O-GlcNAc transferase that participates in gibberellin signal transduction (Thornton et al. 1999); *GASA11* (AT2G18420); *RESPONSE REGULATOR 2*, ARR2 (AT4G16110); *MONOXYGENASE1* (AT4G15760); *ARGOS-LIKE* (AT2G44080; Kuluev et al. 2019); *ETHYLENE INSENSITIVE 1*, (AT1G66340; Poór et al. 2022); ETHYLENE-INSENSITIVE3, EIN3 (AT3G20770); *ETHYLENE-RESPONSIVE NUCLEAR PROTEIN*-like protein gene (AT5G44350); *PHYTOCHROME RED/FAR-RED LIGHT PHOTORECEPTOR* (AT4G16250; Janda et al. 2021); *FAR-RED IMPAIRED RESPONSE1- RELATED SEQUENCE 3* (AT2G27110) that functions in plant growth and development (Ma and Li 2018); *CPL2* (AT5G01270; Covington et al. 2008; Grinevich et al. 2019); *NIGHT LIGHT-INDUCIBLE AND CLOCK-REGULATED GENE 4* (AT5G06980; Kidokoro et al. 2021); *CALCIUM-BINDING EF HAND* family protein gene (AT5G28830); *AtCP1* (AT5G49480; Tuteja and Mahajan, 2007); *Raf-like KINASE* (AT3G06620) that controls vapor pressure difference-mediated stomatal closure (Hsu et al. 2021); the expression of two *NDR1/HIN1-like* genes (AtNHL1 and AtNHL8; Shahbaz et al. 2023); *SABATH METHYLTRANSFERASE* (AT5G38780), catalyzes the methylation of diverse plant metabolites, including phytohormones (Wang et al. 2024); *HOMOLOG OF HUMAN KPNB1* (AT5G53480), an importin-β protein negatively regulated by ABA during drought stress *via* ubiquitination (Oh et al., 2020); *ACYL-COA OXIDASE 1* (AT4G16760) and *ACYL-COA OXIDASE 5* (AT2G35690); *PEROXIN11* (AT3G61070; Orth et al. 2007). *CASEIN KINASE I-LIKE 4* (AT4G28860; Li et al. 2018).	*CONTROL 1* (AT2G46640) influences axillary branch growth angle; *IQ67 DOMAIN protein 21* (AT3G49260) controls adequate indentation formation in pavement cell morphogenesis; *JULGI1* (AT3G15680; Nam et al. 2022); SHOOT GRAVITROPISM-like protein(AT3G48550) that regulates several biochemical processes associated with the plant's response to gravity (Cho et al. 2024); *PHYTOCHROME KINASE SUBSTRATE1* (AT3G44610; Boccalandro et al. 2008); *APYRASE/ GDA1/CD39* nucleoside phosphatase family protein (AT1G14250), proposed to regulate extracellular ATP levels used as a potential signaling signature (Chiu et al. 2015); *MEMBRANE-ASSOCIATED KINASE REGULATOR 3* (AT2G37380; Grison et al. 2019; Novikova et al 2022); *CONCANAVALIN A-LIKE LECTIN* protein kinase family protein gene (AT2G43700); *PAK-box/P21-Rho-BINDING* family protein (AT1G61795), a MAP kinase signaling pathway component; phospholipase *pPLAIIIa* (AT2G39220; Jang et al. 2020); WALL-ASSOCIATED KINASE 2 (AT1G21270; Anderson et al., 2001) *LEUCINE-RICH REPEAT EXTENSIN 5* (AT4G18670; Herger et al. 2019); *ROTUNDIFOLIA like 2* (AT2G29125) and *ROTUNDIFOLIA like 16* (AT3G25717; Valdivia et al. 2012); *BES1/BZR1* homolog 1 (AT3G50750; Kono and Yin 2020); *BRASSINOSTEROID INSENSITIVE 1* (AT4G39400; Jiang et al. 2013); *BAK1-INTERACTING RECEPTOR-LIKE KINASE 3* (AT1G27190); *TCP DOMAIN PROTEIN 10* (AT2G31070; Li 2015); *GA-STIMULATED ARABIDOPSIS 6* (AT1G74670; Qu et al., 2016); *ASPARTIC PROTEASE IN GUARD CELL 1* (AT3G18490; Yao et al. 2012); *SORTING NEXIN 2A* (AT5G58440; Liang 2022).
**Category 8: High MW complex formation**	
ANKYRIN-REPEAT family protein (AT4G10720; Paeng et al. 2020; Kane et al., 2021); *ALBA1* (AT1G76010; Tong et al. 2022); *ACTIN DEPOLYMERIZING FACTOR 5* (AT2G16700) involved in actin cytoskeleton remodeling; *MYOSIN HEAVY CHAIN-LIKE PROTEIN* (AT5G53310); *ALPHA-TUBULIN* (AT1G64740); *ACTIN DEPOLYMERIZING FACTOR 5* (AT2G16700); *MECHANOSENSITIVE CHANNEL OF SMALL CONDUCTANCE-LIKE 4* (AT1G53470; Böddeker et al. 2023), PHOX4 (AT4G32070; Mishra et al., 2018); FORMIN-LIKE PROTEIN (AT3G13370) and *FORMIN HOMOLOGUE 7* (AT1G59910) cytoskeletal-interacting proteins that stimulate actin nucleation and bundling and stabilize microtubules (Chang et al. 2022; Shevchenko and Krutovsky 2022); *ALPHA- TUBULIN* (AT1G64740); *ARMADILLO REPEAT PROTEIN* (AT4G36030); KASH protein *SINE2* (AT3G03970) involved in microtubule reorganization during ABA- induced stomatal closure (Hsiao and Huang 2023); *WD40 repeat-like protein* genes (AT1G15750, AT2G45540 and AT1G36070; Ke et al. 2023).	*KINESIN-12B* (AT3G23670) motor protein that can move actively along microtubules. They are regulators and organizers of the cytoskeleton (Nebenführ and Dixit 2018; Hsiao and Huang 2023; Réthoré et al 2024); CORTICAL *MICROTUBULE DISORDERING 3* (AT4G13370); myosin-M heavy protein (AT2G37960; Sparkes, 2011).

Allakhverdiev, S.I., Kreslavski, V.D., Klimov, V.V., Los, D.A., Carpentier, R., and Mohanty, P. (2008). Heat stress: an overview of molecular responses in photosynthesis. Photosynth. Res. 98: 541-550. doi: 10.1007/s11120-008-9331-0.

Amato, V., Mahalath, S., Zhang, L., Rushton, P. J., & Shen, Q. J. (2025). Structure and functions of NDR1/HIN1-Like (NHL) proteins in plant development and response to environmental stresses. Plant Cell Environ. 48: 5897-5908. https://doi.org/10.1111/pce.15569

Anderson, C.M., Wagner, T.A., Perret, M., He, Z.H., He, D., and Kohorn, B. D. (2001). WAKs: cell wall-associated kinases linking the cytoplasm to the extracellular matrix. Plant Mol. Biol. 47: 197-206. doi: 10.1023/A:1010691701578

Andrási, N., Rigó, G., Zsigmond, L., Pérez-Salamó, I., Papdi, C., Klement, E., Pettkó-Szandtner, A., Baba, A.I., Ayaydin, F., Dasari, R., et al. (2019). The mitogen-activated protein kinase 4- phosphorylated heat shock

factor A4A regulates responses to combined salt and heat stresses. J. Exp. Bot. 70: 4903-4918. doi: 10.1093/jxb/erz217.
Arae, T., Morita, K., Imahori, R., Suzuki, Y., Yasuda, S., Sato, T., Yamaguchi, J., and Chiba, Y. (2019). Identification of Arabidopsis CCR4-NOT complexes with Pumilio RNA-binding proteins, APUM5 and APUM2. Plant Cell Physiol. 60: 2015-2025. doi: 10.1093/pcp/pcz089.
Balfagón D, Gómez-Cadenas A, Rambla JL, Granell A, de Ollas C, Bassham DC, Mittler R, and Zandalinas, S.I. (2022). γ-Aminobutyric acid plays a key role in plant acclimation to a combination of high light and heat stress. Plant Physiol. 188: 2026-2038. doi: 10.1093/plphys/kiac010.
Bechtaoui, N., Rabiu, M.K., Raklami, A., Oufdou, K., Hafidi, M., and Jemo, M. (2021). Phosphate-dependent regulation of growth and stresses management in plants. Front. Plant Sci. 12: 679916. doi: 10.3389/fpls.2021.679916
Boccalandro, H.E., De Simone, S.N., Bergmann-Honsberger, A., Schepens, I., Fankhauser, C., and Casal, J.J. (2008). PHYTOCHROME KINASE SUBSTRATE1 regulates root phototropism and gravitropism. Plant Physiol. 146 :108-115. doi: 10.1104/pp.107.106468.
Böddeker, T.J., Rusch, A., Leeners, K., Murrell, M.P., Dufresne, E.R. (2023). Actin and microtubules position stress granules. PRX Life 1: 023010. doi: https://doi.org/10.1103/PRXLife.1.023010
Bordoloi, K., Dihingia, P., Krishnatreya, D., and Agarwala, N. (2021). Genome-wide identification, characterization and expression analysis of the expansin gene family under drought stress in tea (Camellia sinensis L.). Plant Sci. Today 8: 32-44. DOI: https://doi.org/10.14719/pst.2021.8.1.923.
Burroughs, A.M., and Aravind, L. (2014). A highly conserved family of domains related to the DNA-glycosylase fold helps predict multiple novel pathways for RNA modifications. RNA Biol. 11: 360-372. doi: 10.4161/rna.28302.
Calderón, A., Sevilla, F., and Jiménez, A. (2018). Redox protein thioredoxins: Function under salinity, drought and extreme temperature conditions. In: Gupta, D., Palma, J., Corpas, F. (eds) Antioxidants and Antioxidant Enzymes in Higher Plants. Springer, Cham. https://doi.org/10.1007/978-3-319-75088-0_7.
Cavalier, D.M., Lerouxel, O., Neumetzler, L., Yamauchi, K., Reinecke, A., Freshour, G., Zabotina, O.A., Hahn, M.G., Burgert, I., Pauly, M., et al. (2008) Disrupting two Arabidopsis thaliana xylosyltransferase genes results in plants deficient in xyloglucan, a major primary cell wall component. Plant Cell 20: 1519-1537. doi: 10.1105/tpc.108.059873.
Cecchini, N.M., Torres, J.R., López, I.L., Cobo, S., Nota, F., and Alvarez, M.E. (2022). Alternative splicing of an exitron determines the subnuclear localization of the Arabidopsis DNA glycosylase MBD4L under heat stress. Plant J. 110: 377-388. doi: 10.1111/tpj.15675.
Chang, S., Huang, G., Wang, D., Zhu, W., Shi, J., Yang, L., Liang, W., Xie, Q., and Zhang, D. (2022) Rice SIAH E3 ligases interact with RMD Formin and affect plant morphology. Rice (N Y) 15: 6. doi: 10.1186/s12284-022-00554-8.
Chen, L.Q., Chhajed, S., Zhang, T., Collins, J.M., Pang, Q., Song, W., He, Y., Chen, S. (2021). Protein complex formation in methionine chain-elongation and leucine biosynthesis. Sci Rep. 11: 3524. doi: 10.1038/s41598-021-82790-4.
Chen JG, Wang S. (2017). Involvement of PACLOBUTRAZOL RESISTANCE6/KIDARI, an atypical bHLH transcription factor, in auxin responses in Arabidopsis. Front Plant Sci. 8: 1813. https://doi.org/10.3389/fpls.2017.01813
Chen, M., Xu, J., Devis, D., Shi, J., Ren, K., Searle, I., and Zhang, D. (2016). Origin and functional prediction of pollen allergens in plants. Plant Physiol. 172: 341-357. doi: 10.1104/pp.16.00625. Chen, B., Wang, X., Yu, H., Dong, N., Li, J., Chang, X., Wang, J., Jiang, C., Liu, J., Chi, X. et al. (2024). Genome-wide analysis of UDP-glycosyltransferases family and identification of UGT genes involved in drought stress of Platycodon grandiflorus. Front. Plant Sci. 15: 1363251. doi: 10.3389/fpls.2024.1363251.
Chi, C., Xu, X., Wang, M., Zhang, H., Fang, P., Zhou, J., Xia, X., Shi, K., Zhou, Y., Yu, J. (2021). Strigolactones positively regulate abscisic acid-dependent heat and cold tolerance in tomato. Hortic Res. 8: 237. doi: 10.1038/s41438-021.
Chirinos-Arias, M.C., and Spampinato, C.P. (2021). Role of the mismatch repair protein MSH7 in Arabidopsis adaptation to acute salt stress. Plant Physiol. Biochem. 169: 280-290. doi: 10.1016/j.plaphy.2021.11.029.
Cho Y, Kim Y, Lee H, Kim S, Kang J, Kadam US, Ju Park S, Sik Chung W, and Chan Hong, J. (2024). Cellular and physiological functions of SGR family in gravitropic response in higher plants. J. Adv. Res. 1: S2090-1232(24)00039-0. doi: 10.1016/j.jare.2024.01.026.
Chiu, T.Y., Lao, J., Manalansan, B., Loqué, D., Roux, S.J., and Heazlewood, J.L. (2015). Biochemical characterization of Arabidopsis APYRASE family reveals their roles in regulating endomembrane NDP/NMP homoeostasis. Biochem. J. 472: 43-54. doi: 10.1042/BJ20150235.
Coculo, D., and Lionetti, V. (2022). The plant invertase/pectin methylesterase inhibitor superfamily. Front. Plant Sci. 13: 863892. doi: 10.3389/fpls.2022.863892.
Colombatti, F., Mencia, R., Garcia, L., Mansilla, N., Alemano, S., Andrade, A.M., Gonzalez, D.H., and Welchen, E. (2019). The mitochondrial oxidation resistance protein AtOXR2 increases plant biomass and tolerance to oxidative stress. J. Exp. Bot. 70: 3177-3195. doi: 10.1093/jxb/erz147.
Covington, M.F., Maloof, J.N., Straume, M., Kay, S,A., and Harmer, S.L. (2008). Global transcriptome analysis reveals circadian regulation of key pathways in plant growth and development. Genome Biol. 9: R130. doi: 10.1186/gb-2008-9-8-r130.
Daněk, M., Valentová, O., Martinec, J. (2016). Flotillins, Erlins, and HIRs: from animal base camp to plant new horizons. Crit. Rev. Plant Sci. 35: 191-214. https://doi.org/10.1080/07352689.2016.1249690.
Dean, P.J., Siwiec, T., Waterworth, W.M., Schlögelhofer, P., Armstrong, S.J., and West, C.E. (2009). A novel ATM-dependent X-ray-inducible gene is essential for both plant meiosis and gametogenesis. Plant J. 58: 791-802. doi: 10.1111/j.1365-313X.2009.03814.x
de Francisco Amorim, M., Willing, E.M., Szabo, E.X., Francisco-Mangilet, A.G., Droste-Borel, I., Maček, B., Schneeberger, K., and Laubinger, S. (2018). The U1 snRNP subunit LUC7 modulates plant development and stress responses via regulation of alternative splicing. Plant Cell 30: 2838-2854. doi: 10.1105/tpc.18.00244.
Denzler, A. (2022). Initiating polar growth in plant cells-Functions of RopGEFs during root hair development in Arabidopsis thaliana (Doctoral dissertation, Ruperto Carola University Heidelberg, Germany). doi: 10.11588/heidok.00030005.
Ding, Y., Ndamukong, I., Zhao, Y., Xia, Y., Riethoven, J.J., Jones, D.R., Divecha, N., and Avramova, Z. (2012). Divergent functions of the myotubularin (MTM) homologs AtMTM1 and AtMTM2 in Arabidopsis thaliana: evolution of the plant MTM family. Plant J. 70: 866-878. doi: 10.1111/j.1365-313X.2012.04936.x.
Diyang, Q., Rui, H., Ji, L., Ying, L., Jierong, D., Kuaifei, X., Xuhua, Z., Zhongming, F., and Mingyong, Z. (2023). Peptide transporter OsNPF8. 1 contributes to sustainable growth under salt and drought stresses, and grain yield under nitrogen deficiency in rice. Rice Sci. 30: 113-126. https://doi.org/10.1016/j.rsci.2023.01.004.
Ezquer, I., Salameh, I., Colombo, L., and Kalaitzis, P. (2020). Plant cell walls tackling climate change: Biotechnological strategies to improve crop adaptations and photosynthesis in response to global warming. Plants 9: 212. doi:10.3390/plants9020212.
Figueroa, C., Taylor, J., and Vojtek, A.B. (2001). Prenylated Rab acceptor protein is a receptor for prenylated small GTPases. J, Biol. Chem. 276: 28219-28225. doi: 10.1074/jbc.M101763200. Fortunato, S., Lasorella, C., Dipierro, N., Vita, F., and de Pinto, M.C. (2023). Redox signaling in plant heat stress response. Antioxidants (Basel) 12: 605. doi: 10.3390/antiox12030605.
Fu, X., Yu, L.J., Mao-Teng, L., Wei, L., Wu, C., and Yun-Feng, M. (2008). Evolution of structure in gamma-class carbonic anhydrase and structurally related proteins. Mol. Phylogenet. Evol. 47: 211-220. doi: 10.1016/j.ympev.2008.01.005.

Gallego-Giraldo, L., Liu, C., Pose-Albacete, S., Pattathil, S., Peralta, A.G., Young, J., Westpheling, J., Hahn, M.G., Rao, X., Knox, J.P., et al., (2020). ARABIDOPSIS DEHISCENCE ZONE POLYGALACTURONASE 1 (ADPG1) releases latent defense signals in stems with reduced lignin content. Proc. Natl. Acad. Sci. USA 117: 3281-3290. doi: 10.1073/pnas.1914422117.

Gelová, Z., Gallei, M., Pernisová, M., Brunoud, G., Zhang, X., Glanc, M., Li, L., Michalko, J., Pavlovičová, Z., Verstraeten, I., et al. (2021). Developmental roles of auxin binding protein 1 in *Arabidopsis thaliana*. Plant Sci. 303: 110750. doi: 10.1016/j.plantsci.2020.110750.

Grinevich, D.O., Desai, J.S., Stroup, K.P., Duan, J., Slabaugh, E., and Doherty, C.J. (2019). Novel transcriptional responses to heat revealed by turning up the heat at night. Plant Mol. Biol. 101: 1-19. doi: 10.1007/s11103-019-00873-3.

Grison, M.S., Kirk, P., Brault, M.L., Wu, X.N., Schulze, W.X., Benitez-Alfonso, Y., Immel, F., and Bayer, E.M. (2019). Plasma membrane-associated receptor-like kinases relocalize to plasmodesmata in response to osmotic stress. Plant Physiol. 181: 142-160. doi: 10.1104/pp.19.00473

Goodfellow, B.J., Freire, F., Carvalho, A.L., Aveiro, S.S., Charbonnier, P., Moulis, J.M., Delgado, L., Ferreira, G.C., Rodrigues, J.E., Poussin-Courmontagne, P.P., et al. (2021). The SOUL family of heme-binding proteins: Structure and function 15 years later. Coor. Chem. Rev. 448: 214189, https://doi.org/10.1016/j.ccr.2021.214189.

Haider, S., Iqbal, J., Shaukat, M., Naseer, S., and Mahmood, T. (2021). The epigenetic chromatin-based regulation of somatic heat stress memory in plants. Plant Gene 27: 100318. https://doi.org/10.1016/j.plgene.2021.100318.

Han, G., Qiao, Z., Li, Y., Yang, Z., Wang, C., Zhang, Y., Liu, L., and Wang, B. (2022). RING zinc finger proteins in plant abiotic stress tolerance. Front. Plant Sci. 13: 877011. doi: 10.3389/fpls.2022.877011. He, J., Zhao, H., Cheng, Z., Ke, Y., Liu, J., and Ma, H. (2019). Evolution analysis of the Fasciclin-like arabinogalactan proteins in plants shows variable Fasciclin-AGP domain constitutions. Int. J. Mol. Sci. 20: 1945. doi: 10.3390/ijms20081945.

Henriet, C., Balliau, T., Aimé, D., Le Signor, C., Kreplak, J., Zivy, M., Gallardo, K., and Vernoud, V. (2021). Proteomics of developing pea seeds reveals a complex antioxidant network underlying the response to sulfur deficiency and water stress. J. Exp. Bot. 72: 2611-2626. doi:10.1093/jxb/eraa571.

Herger, A., Dünser, K., Kleine-Vehn, J., and Ringli, C. (2019). Leucine-rich repeat extensin proteins and their role in cell wall sensing. Curr. Biol. 29: R851-R858. doi: 10.1016/j.cub.2019.07.039. Hirakawa, T., Tanno, S., and Ohara, K. (2023). N-acetylglutamic acid alleviates oxidative stress based on histone acetylation in plants. Front. Plant Sci. 14: 1165646. doi: 10.3389/fpls.2023.1165646. Hsiao AS, and Huang, J.Y. (2023). Microtubule regulation in plants: From morphological development to stress adaptation. Biomolecules 13: 627. doi: 10.3390/biom13040627.

Hsu, P.K., Takahashi, Y., Merilo, E., Costa, A., Zhang, L., Kernig, K., Lee, K.H., and Schroeder, J.I. (2021). Raf-like kinases and receptor-like (pseudo)kinase GHR1 are required for stomatal vapor pressure difference response. Proc. Natl. Acad. Sci. USA 118: e2107280118. doi: 10.1073/pnas.2107280118. PMID: 34799443.

Hu, Y., Liu, Y., Lu, L., Tao, J. J., Cheng, T., Jin, M., Wang, Z.Y., Wei, J.J., Jiang, Z.H, Sun, W.C, Liu, C.L, Gao, F., Zhang, Y., Li, W., Bi, Y.D., Lai, Y.C, Zhou, B., Yu, D.Y., Yin, C.C., Wei, W., Zhang, W.K, Chen, S.Y., Zhang, J.S. (2023). Global analysis of seed transcriptomes reveals a novel PLATZ regulator for seed size and weight control in soybean. New Phytol. 240: 2436-2454. https://doi.org/10.1111/nph.19316

Islam, M.N., Rabby, M.G., Hossen, M.M., Bonny, M., and Hasan, M.M. (2024). Genome-wide identification following functional analysis of amino acid permease and cationic amino acid transporter gene families in maize and their role in drought stress. S. Afr. J. Bot. 168: 360-371.https://doi.org/10.1016/j.sajb.2024.03.029.

Isono, K., Tsukimoto, R., Iuchi, S., Shinozawa, A., Yotsui, I., Sakata, Y., and Taji, T. (2021). An ER-Golgi tethering factor SLOH4/MIP3 is involved in long-term heat tolerance of Arabidopsis. Plant Cell Physiol. 62: 272-279. doi: 10.1093/pcp/pcaa157.

Jiang, J., Zhang, C., and Wang, X. (2013). Ligand perception, activation, and early signaling of plant steroid receptor brassinosteroid insensitive 1. J. Integr. Plant Biol. 55: 1198-211. doi: 10.1111/jipb.12081. Janda, T., Prerostová, S., Vanková, R., and Darkó, É. (2021). Crosstalk between light- and temperature-mediated processes under cold and heat stress conditions in plants. Int. J, Mol. Sci. 22: 8602. doi: 10.3390/ijms22168602.

Jang, J.H., Nguyen, N.Q., Légeret, B., Beisson, F., Kim, Y.J., Sim, H.J., and Lee, O.R. (2020). Phospholipase pPLAIIIα increases germination rate and resistance to Turnip Crinkle Virus when overexpressed. Plant Physiol. 184: 1482-1498. doi: 10.1104/pp.20.00630.

Jin, R., Yang, H., Muhammad, T., Li, X., Tuerdiyusufu, D., Wang, B., and Wang, J. (2024). Involvement of Alfin-like transcription factors in plant development and stress response. Genes (Basel) 15: 184. doi: 10.3390/genes15020184.

Jung, J. H., Lee, S., Yun, J., Lee, M., Park, C. M. (2014). The miR172 target TOE3 represses *AGAMOUS* expression during *Arabidopsis* floral patterning. Plant Sci. 215: 29-38. https://doi.org/10.1016/j.indcrop.2021.113853 https://doi.org/10.1016/j.plantsci.2013.10.010

Kan, Y., Mu, X.R., Zhang, H., Gao, J., Shan, J.X., Ye, W.W., and Lin, H.X. (2022). TT2 controls rice thermotolerance through SCT1-dependent alteration of wax biosynthesis. Nat. Plants 8: 53-67. https://doi.org/10.1038/s41477-021-01039-0.

Kane, E.I., and Spratt, D.E. (2021). Structural insights into ankyrin repeat-containing proteins and their influence in ubiquitylation. Int. J. Mol. Sci. 22: 609. https://doi.org/10.3390/ijms22020609. Kanno, Y., Hanada, A., Chiba, Y., Ichikawa, T., Nakazawa, M., Matsui, M., Koshiba, T., Kamiya, Y., and Seo, M. (2012). Identification of an abscisic acid transporter by functional screening using the receptor complex as a sensor. Proc. Natl. Acad. Sci. USA 109: 9653-9658. doi: 10.1073/pnas.1203567109.

Kanno, Y., Oikawa, T., Chiba, Y., Ishimaru, Y., Shimizu, T., Sano, N., Koshiba, T., Kamiya, Y., Ueda, M., and Seo, M. (2016). AtSWEET13 and AtSWEET14 regulate gibberellin-mediated physiological processes. Nat. Commun. 7: 13245. doi: 10.1038/ncomms13245.

Kapoor, R.T., Alam, P., Chen, Y., and Ahmad, P. (2024). Strigolactones in plants: from development to abiotic stress management. J. Plant Growth Regul. 43: 903-919. https://doi.org/10.1007/s00344-023-11148-z.

Ke, S., Jiang, Y., Zhou, M., and Li, Y. (2023). Genome-wide identification, evolution, and expression analysis of the WD40 subfamily in Oryza Genus. Int. J. Mol. Sci. 24: 15776. https://doi.org/10.3390/ ijms242115776.

Khan, M.N., Ahmed, I., Ud Din, I., Noureldeen, A., Darwish, H., and Khan, M. (2022). Proteomic insight into soybean response to flooding stress reveals changes in energy metabolism and cell wall modifications. PLoS ONE 17: e0264453. doi: 10.1371/journal.pone.0264453.

Kidokoro, S., Hayashi, K., Haraguchi, H., Ishikawa, T., Soma, F., Konoura, I., Toda, S., Mizoi, J., Suzuki, T., Shinozaki, K., et al. (2021). Posttranslational regulation of multiple clock-related transcription factors triggers cold-inducible gene expression in Arabidopsis. Proc. Natl. Acad. Sci. USA 118: e2021048118. doi: 10.1073/pnas.2021048118.

Kirschner, G.K., Rosignoli, S., Guo, L., Vardanega, I., Imani, J., Altmüller, J., Milner, S.G., Balzano, R., Nagel, K.A., Pflugfelder, D., et al. (2021). ENHANCED GRAVITROPISM 2 encodes a STERILE ALPHA MOTIF-containing protein that controls root growth angle in barley and wheat. Proc. Natl. Acad. Sci. USA 118: e2101526118. doi: 10.1073/pnas.2101526118. Kishorekumar, R., Bulle, M., Wany, A., and Gupta., K.J. (2020) An overview of important enzymes involved in nitrogen assimilation of Plants. Methods Mol. Biol. 2057: 1-13. doi: 10.1007/978-1-4939-9790-9_1.

Kok, Z., Kuo, Y.W., Soh, Z.T., Huang, H.C., Tseng, B.S., Hsieh, H.C., Tsai, W.A., Jeng, S.T., Chen, S.P., and Lin, J.S. (2023). Regulatory roles of microRNA163 in responses to stresses in Arabidopsis. Physiol. Plant. 175: e14053. https://doi.org/10.1111/ppl.14053.

Kono, A., and Yin, Y. (2020). Updates on BES1/BZR1 regulatory networks coordinating plant growth and stress responses. Front. Plant Sci. 11: 617162. doi: 10.3389/fpls.2020.617162

Korte, P., Unzner, A., Damm, T., Berger, S., Krischke, M., and Mueller, M.J. (2023). High triacylglycerol turnover is required for efficient opening of stomata during heat stress in *Arabidopsis*. Plant

J. 115: 81-96. doi: 10.1111/tpj.16210.

Kuluev, B., Mikhaylova, E., Ermoshin, A., Veselova, S., Tugbaeva, A., Gumerova, G., Gainullina, K., and Zaikina, E. (2019). The ARGOS-LIKE genes of Arabidopsis and tobacco as targets for improving plant productivity and stress tolerance. J. Plant Physiol. 242: 153033. doi: 10.1016/j.jplph.2019.153033.

Lambour, B., Glenz, R., Forner, C., Krischke, M., Mueller, M.J., Fekete, A., and Waller, F. (2022). Sphingolipid long-chain base phosphate degradation can be a rate-limiting step in long-chain base homeostasis. Front. Plant Sci. 13: 911073. doi: 10.3389/fpls.2022.911073.

Lei, S., Rossi, S., and Huang, B. (2022). Metabolic and physiological regulation of aspartic acid-mediated enhancement of heat stress tolerance in perennial ryegrass. Plants (Basel) 11: 199. doi: 10.3390/plants11020199.

Li, F., Asami, T., Wu, X., Tsang, E.W., and Cutler, A.J. (2007). A putative hydroxysteroid dehydrogenase involved in regulating plant growth and development. Plant Physiol. 145: 87-97. doi: 10.1104/pp.107.100560.

Li, S. (2015) The *Arabidopsis thaliana* TCP transcription factors: A broadening horizon beyond development. Plant Signal. Behav. 10: e1044192. doi: 10.1080/15592324.2015.1044192.

Li, L., Chen, G., Yuan, M., Guo, S., Wang, Y., and Sun, J. (2022). CsbZIP2-miR9748-CsNPF4.4 module mediates high temperature tolerance of cucumber through jasmonic acid pathway. Front. Plant Sci. 13: 883876. doi: 10.3389/fpls.2022.883876.

Li, Z., Deng, S., Zhu, D., Wu, J., Zhou, J., Shi, Fayyaz, P., Luo, Z., and Luo, J. (2023). Proteomic reconfigurations underlying physiological alterations in poplar roots in acclimation to changing nitrogen availability. Environ. Exp. Bot. 211: 105367. https://doi.org/10.1016/j.envexpbot.2023.105367.

Li, M., Hao, P., Zhang, J., Yang, X., Wu, A., Zhang, M., Wei, H., Fu, X., Wang, H., Yu, S. (2021). A comprehensive identification and function analysis of the ATBS1 Interacting Factors (AIFs) gene family of *Gossypium* species in fiber development and under multiple stresses. Industrial Crops and Products, 171, 113853. doi: 10.3389/fpls.2017.01813.

Li, Y., Min, L., Zhang, L., Hu, Q., Wu, Y., Li, J., Xie, S., Ma, Y., Zhang, X., and Zhu, L. (2018). Promoters of Arabidopsis Casein kinase I-like 2 and 7 confer specific high-temperature response in anther. Plant Mol. Biol. 98: 33-49. doi: 10.1007/s11103-018-0760-7.

Li Y, Xu J, Xu M, Yang Y, Cheng Y, Shang Z, Kang E. (2025). ICE1 (Inducer of CBF Expression 1) is essential for the jasmonate-regulated development of stamen in *Arabidopsis thaliana*. Plant Cell Environ. 48: 3810-3826. doi: 10.1111/pce.15389.

Liang, C., Li, C., Wu, J., Zhao, M., Chen, D., Liu, C., Chu, J., Zhang, W., Hwang, I., and Wang, M. (2022). SORTING NEXIN2 proteins mediate stomatal movement and the response to drought stress by modulating trafficking and protein levels of the ABA exporter ABCG25. Plant J. 110: 1603-1618. doi: 10.1111/tpj.15758.

Llamas, E., Pulido, P., and Rodriguez-Concepcion, M. (2017). Interference with plastome gene expression and Clp protease activity in Arabidopsis triggers a chloroplast unfolded protein response to restore protein homeostasis. PLoS Genet. 13: e1007022. doi: 10.1371/journal.pgen.1007022.

López-Marqués, R.L., Davis, J.A., Harper, J.F., and Palmgren, M. (2021) Dynamic membranes: the multiple roles of P4 and P5 ATPases. Plant Physiol. 185: 619-631. doi: 10.1093/plphys/kiaa065. PMID: 33822217.

Luo, Y., Xie, Y., Li, W., Wei, M., Dai, T., Li, Z., and Wang, B. (2021). Physiological and transcriptomic analyses reveal exogenous trehalose is involved in the responses of wheat roots to high temperature stress. Plants (Basel) 10: 2644. doi: 10.3390/plants10122644.

Luo, P., Li, T.T., Shi, W.M., Ma, Q., and Di, D.W. (2023). The roles of GRETCHEN HAGEN3 (GH3)-dependent auxin conjugation in the regulation of plant development and stress adaptation. Plants (Basel) 12: 4111. doi: 10.3390/plants12244111.

Ma, S., Ding, Z., and Li, P. (2017). Maize network analysis revealed gene modules involved in development, nutrients utilization, metabolism, and stress response. BMC Plant Biol., 17: 1-17. https://doi.org/10.1186/s12870-017-1077-4.

Ma L and Li, G. (2018). FAR1-RELATED SEQUENCE (FRS) and FRS-RELATED FACTOR (FRF) Family Proteins in Arabidopsis Growth and Development. Front Plant Sci. 2018 Jun 7;9:692. doi: 10.3389/fpls.2018.00692.

Madhu, Sharma, A., Kaur, A., Tyagi, S., and Upadhyay, S.K. (2023). Glutathione peroxidases in plants: innumerable role in abiotic stress tolerance and plant development. J. Plant Growth Regul. 42: 598-613. https://doi.org/10.1007/s00344-022-10601-9.

Mishra, D., Shekhar, S., Chakraborty, S., and Chakraborty, N. (2018). Carboxylate clamp tetratricopeptide repeat (TPR) domain containing Hsp90 cochaperones in Triticeace: An insight into structural and functional diversification. Environ. Exp. Bot. 155: 31-44. https://doi.org/10.1016/j.envexpbot.2018.06.020.

Modliszewski, J.L., Wang, H., Albright, A.R., Lewis, S.M., Bennett, A.R., Huang, J., Ma, H., Wang, Y., and Copenhaver, G.P. (2018). Elevated temperature increases meiotic crossover frequency via the interfering (Type I) pathway in Arabidopsis thaliana. PLoS Genet. 14: e1007384. doi: 10.1371/journal.pgen.1007384.

Murray, A.W. (2004). Recycling the cell cycle: cyclins revisited. Cell 116: 221-234. doi: 10.1016/s0092-8674(03)01080-8.

Nam, H., Gupta, A., Nam, H., Lee, S., Cho, H.S., Park, C., Park, S., Park, S.J., and Hwang, I. (2022). JULGI-mediated increment in phloem transport capacity relates to fruit yield in tomato. Plant Biotechnol. J. 20: 1533-1545. doi: 10.1111/pbi.13831.

Naughton, F.B., Kalli, A.C., and Sansom, M.S.P. (2018). Modes of interaction of Pleckstrin homology domains with membranes: Toward a computational biochemistry of membrane recognition. J. Mol. Biol. 430: 372-388. doi: 10.1016/j.jmb.2017.12.011.

Nebenführ, A., and Dixit, R. (2018). Kinesins and myosins: Molecular motors that coordinate cellular functions in plants. Annu. Rev. Plant Biol. 69: 329-361. doi: 10.1146/annurev-arplant-042817- 040024.

Niu, W.T., Han, X.W., Wei, S.S., Shang, Z.L., Wang, J., Yang, D.W., Fan, X., Gao, F., Zheng, S.Z., Bai, J.T., et al. (2020). Arabidopsis cyclic nucleotide-gated channel 6 is negatively modulated by multiple calmodulin isoforms during heat shock. J Exp. Bot. 71: 90-104. doi: 10.1093/jxb/erz445.

Ngoc, L.N.T., Park, S.J., Cai, J., Huong, T.T., Lee, K., and Kang, H. (2021). RsmD, a chloroplast rRNA m2G methyltransferase, plays a role in cold stress tolerance by possibly affecting chloroplast translation in Arabidopsis. Plant Cell Physiol. 62: 948-958. doi: 10.1093/pcp/pcab060.

Novikova, D.D., Korosteleva, A.L., Mironova, V., and Jaillais, Y. (2022). Meet your MAKR: the membrane-associated kinase regulator protein family in the regulation of plant development. FEBS J. 289: 6172-6186. doi: 10.1111/febs.16132.

Oh, T.R., Yu, S.G., Yang, H.W., Kim, J.H., and Kim, W.T. (2020). AtKPNB1, an Arabidopsis importin-β protein, is downstream of the RING E3 ubiquitin ligase AtAIRP1 in the ABA-mediated drought stress response. Planta 252: 93. doi: 10.1007/s00425-020-03500-4.

Orth, T., Reumann, S., Zhang, X., Fan, J., Wenzel, D., Quan, S., and Hu, J. (2007). The PEROXIN11 protein family controls peroxisome proliferation in Arabidopsis. Plant Cell 19: 333-350. doi: 10.1105/tpc.106.045831.

Paeng, S.K., Kang, C.H., Chi, Y.H., Chae, H.B., Lee, E.S., Park, J.H., Wi, S.D., Bae, S.B., Phan, K.A.T., and Lee, S.Y. (2020). AtTPR10 containing multiple ANK and TPR domains exhibits chaperone activity and heat-shock dependent structural switching. Appl. Sci. 10: 1265. https://doi.org/10.3390/app10041265.

Palusa, S.G., Golovkin, M., Shin, S.B., Richardson, D.N., and Reddy, A.S.N. (2007). Organ-specific, developmental, hormonal and stress regulation of expression of putative pectate lyase genes in Arabidopsis. New Phytol. 174: 537-550. doi: 10.1111/j.1469-8137.2007.02033.x.

Pandey, S., Prasad, A., Sharma, N., and Prasad, M. (2020). Linking the plant stress responses with RNA helicases. Plant Sci. 299: 110607. doi: 10.1016/j.plantsci.2020.110607.

Paradiso, A., Domingo, G., Blanco, E., Buscaglia, A., Fortunato, S., Marsoni, M., Scarcia, P., Caretto, S., Vannini, C., and de Pinto, M.C. (2020). Cyclic AMP mediates heat stress response by the control of redox homeostasis

and ubiquitin-proteasome system. Plant Cell Environ. 43: 2727-2742. doi: 10.1111/pce.13878.
Park, Y.J., Kim, J.Y., and Park, C.M. (2022). SMAX1 potentiates phytochrome B-mediated hypocotyl thermomorphogenesis. Plant Cell 34: 2671-2687. doi: 10.1093/plcell/koac124.
Patnaik, A., Behera, A., Kumar, A., Dalai, A., Mukundan, S., Priyadarshini, N., Panigrahy, M., and Panigrahi, K.C. (2024). Light and temperature-dependent developmental role of Auxin Binding Protein 1 (ABP1) in Arabidopsis thaliana. bioRxiv: 2024-01. https://doi.org/10.1101/2024.01.03.574050.
Pfalz, M., Mikkelsen, M.D., Bednarek, P., Olsen, C.E., Halkier, B.A., and Kroymann, J. (2011). Metabolic engineering in Nicotiana benthamiana reveals key enzyme functions in Arabidopsis indole glucosinolate modification. Plant Cell 23: 716-29. doi: 10.1105/tpc.110.081711.
Poór, P., Nawaz, K., Gupta, R., Ashfaque, F., and Khan, M.I.R. (2022). Ethylene involvement in the regulation of heat stress tolerance in plants. Plant Cell Rep. 41: 675-698. doi: 10.1007/s00299- 021-02675-8.
Potuschak, T., Stary, S., Schlögelhofer, P., Becker, F., Nejinskaia, V., and Bachmair, A. (1998). PRT1 of Arabidopsis thaliana encodes a component of the plant N-end rule pathway. Proc. Natl. Acad. Sci. USA 95: 7904-7908. doi: 10.1073/pnas.95.14.7904.
Qi, H., Xia, F.N., Xie, L.J., Yu, L.J., Chen, Q.F., Zhuang, X.H., Wang, Q., Li, F., Jiang, L., Xie, Q., et al. (2017). TRAF family proteins regulate autophagy dynamics by modulating AUTOPHAGY PROTEIN6 stability in Arabidopsis. Plant Cell 29: 890-911. doi: 10.1105/tpc.17.00056.
Qi, H., Kang, D., Zeng, W., Jawad Hassan, M., Peng, Y., Zhang, X., Zhang, Y., Feng, G., and Li, Z. (2021). Alterations of endogenous hormones, antioxidant metabolism, and aquaporin gene expression in relation to γ-aminobutyric acid-regulated thermotolerance in white clover. Antioxidants (Basel) 10: 1099. doi: 10.3390/antiox10071099.
Qin, Y., Ye, H., Tang, N., and Xiong, L. (2009). Systematic identification of X1-homologous genes reveals a family involved in stress responses in rice. Plant Mol. Biol. 71: 483-96. doi: 10.1007/s11103- 009-9535-5.
Rehman, A., Shahzad, B., Haider, F.U., Ullah, A., and Khan, I. (2022). Brassinosteroids in plant response to high temperature stress. In Brassinosteroids in Plant Developmental Biology and Stress Tolerance (pp. 173-187). Academic Press. https://doi.org/10.1016/B978-0-12-813227-2.00014-X.
Qu, J., Kang, S.G., Hah, C., and Jang J.C. (2016). Molecular and cellular characterization of GA-Stimulated Transcripts GASA4 and GASA6 in Arabidopsis thaliana. Plant Sci. 246: 1-10. doi: 10.1016/j.plantsci.2016.01.009.
Reichelt, N., Korte, A., Krischke, M., Mueller, M.J., and Maag, D. (2023). Natural variation of warm temperature-induced raffinose accumulation identifies TREHALOSE-6-PHOSPHATE SYNTHASE 1 as a modulator of thermotolerance. Plant Cell Environ. 46: 3392-3404. doi: 10.1111/pce.14664.
Réthoré, E., Pelletier, S., Balliau, T., Zivy, M., Avelange-Macherel, M.H., Macherel, D. (2024). Multi-scale analysis of heat stress acclimation in Arabidopsis seedlings highlights the primordial contribution of energy-transducing organelles. Plant J. 119: 300-331. https://doi.org/10.1111/tpj.16763
Reyes, F., Marchant, L., Norambuena, L., Nilo, R., Silva, H., and Orellana, A. (2006). AtUTr1, a UDP-glucose/UDP-galactose transporter from Arabidopsis thaliana, is located in the endoplasmic reticulum and up-regulated by the unfolded protein response. J. Biol. Chem. 281: 9145-9151. doi: 10.1074/jbc.M512210200.
Riechmann, J.L. (2023). A new negative link in flower development: Repression of *ABC* genes by Z factors-ZP1/ZFP8. Proc. Natl. Acad. Sci. U.S.A. 120: e2307429120. doi: 10.1073/pnas.2307429120. Roach, T.G., Lång, H.K.M., Xiong, W., Ryhänen, S.J., and Capelluto, D.G.S. (2021). Protein trafficking or cell signaling: A dilemma for the adaptor protein TOM1. Front. Cell Dev. Biol. 9: 643769. doi: 10.3389/fcell.2021.643769.
Rodríguez-Saavedra, C., Morgado-Martínez, L.E., Burgos-Palacios, A., King-Díaz, B., López-Coria, M., and Sánchez-Nieto, S. (2021). Moonlighting proteins: The case of the hexokinases. Front. Mol. Biosci. 8: 701975. doi: 10.3389/fmolb.2021.701975.
Sámano, M.L., Nanjareddy, K., Arthikala, M.K. (2024) NIN-like proteins (NLPs) as crucial nitrate sensors: an overview of their roles in nitrogen signaling, symbiosis, abiotic stress, and beyond. Physiol. Mol. Biol. Plants 30: 1209-1223. https://doi.org/10.1007/s12298-024-01485-y
Sehar, Z., Gautam, H., Iqbal, N., Alvi, A.F., Jahan, B., Fatma, M., Albaqami, M., and Khan, N.A. (2022). The functional interplay between ethylene, hydrogen sulfide, and sulfur in plant heat stress tolerance. Biomolecules 12: 678. doi: 10.3390/biom12050678
Segev, N. (2001). Ypt/rab gtpases: regulators of protein trafficking. Sci STKE. 2001: re11. doi: 10.1126/stke.2001.100.re11.
Sessions, A., Nemhauser, J.L., McColl, A., Roe, J.L., Feldmann, K.A., and Zambryski, P.C. (1997). ETTIN patterns the Arabidopsis floral meristem and reproductive organs. Development 124: 4481- 4491. doi: 10.1242/dev.124.22.4481.
Shahbaz, M., Azeem, F., Rafique, M.U., Siraj, H.M.S. and Rizwan, M. (2023). Heat-induced transcriptome and genome-wide analysis of NHL genes in maize (Zea mays L.) suggest a role of ZmNHLs under heat stress. J. Plant Growth Regul. 42: 6891-6902. https://doi.org/10.1007/s00344-023-10982-5.
Shang, J.Y, Lu, Y.J., Cai, X.W., Su, Y.N., Feng, C., Li, L., Chen, S., and He, X.J. (2021). COMPASS functions as a module of the INO80 chromatin remodeling complex to mediate histone H3K4 methylation in Arabidopsis. Plant Cell 33: 3250-3271, https://doi.org/10.1093/plcell/koab187.
Shevchenko, G.V., and Krutovsky, K.V. (2022). Mechanical stress effects on transcriptional regulation of genes encoding microtubule-and actin-associated proteins. Physiol. Mol. Biol. Plants 28: 17-30. https://doi.org/10.1007/s12298-021-01123-x.
Shin, J.S., Kim, S.Y., So, W.M., Noh, M., Yoo, K.S., and Shin, J.S. (2020). LON domain-containing protein 1 represses thioredoxin y2 and regulates ROS levels in Arabidopsis chloroplasts. FEBS Lett. 594: 986-994. doi: 10.1002/1873-3468.13664.
Singh, V.P., Jaiswal, S., Wang, Y., Feng, S., Tripathi, D.K., Singh, S., Gupta, R., Xue, D., Xu, S., and Chen, Z.H. (2024). Evolution of reactive oxygen species cellular targets for plant development. Trends Plant Sci. 21: S1360-1385. doi: 10.1016/j.tplants.2024.03.005.
Šola, K., Dean, G.H., Li, Y., Lohmann, J., Movahedan, M., Gilchrist, E.J., Adams, K.L., and Haughn, G.W. (2021). Expression patterns and functional characterization of arabidopsis GALACTOSE OXIDASE-LIKE genes suggest specialized roles for galactose oxidases in plants. Plant Cell Physiol. 62: 1927-1943. doi: 10.1093/pcp/pcab073. PMID: 34042158.
Soto, M.J., Urbanowicz, B.R., and Hahn, M.G. (2019). Plant fucosyltransferases and the emerging biological importance of fucosylated plant structures. Crit. Rev. Plant Sci. 38: 327-338. https://doi.org/10.1080/07352689.2019.1673968.
Sun, H., Ren, M., and Zhang, J. (2022). Genome-wide identification and expression analysis of fibrillin (FBN) gene family in tomato (Solanum lycopersicum L.). PeerJ, 10: e13414. doi 10.7717/peerj.13414. Sparkes, I. (2011). Recent advances in understanding plant myosin function: life in the fast lane. Mol. Plant. 4: 805-812. doi: 10.1093/mp/ssr063.
Thirumalaikumar, V.P., Gorka, M., Schulz, K., Masclaux-Daubresse, C., Sampathkumar, A., Skirycz, A., Vierstra, R.D., and Balazadeh, S. (2021). Selective autophagy regulates heat stress memory in Arabidopsis by NBR1-mediated targeting of HSP90.1 and ROF1. Autophagy 17: 2184-2199. doi: 10.1080/15548627.2020.1820778.
Thornton T.M., Swain, S.M., and Olszewski, N.E. (1999). Gibberellin signal transduction presents ellipsisthe SPY who O-GlcNAc'd me. Trends Plant Sci. 4: 424-428. doi: 10.1016/s1360- 1385(99)01485-5.
Tian, F., Gong, J., Zhang, J., Zhang, M., Wang, G., Li, A., and Wang, W. (2013). Enhanced stability of thylakoid membrane proteins and antioxidant competence contribute to drought stress resistance in the tasg1 wheat stay-green mutant. J. Exp. Bot. 64: 1509-20. doi: 10.1093/jxb/ert004.
Tong, J., Ren, Z., Sun, L., Zhou, S., Yuan, W., Hui, Y., Ci, D., Wang, W., Fan, L.M., Wu, Z., et al. (2022) ALBA proteins confer thermotolerance through stabilizing HSF messenger RNAs in cytoplasmic granules. Nat. Plants. 8: 778-791. doi: 10.1038/s41477-022-01175-1.
Tuteja, N., and Mahajan, S. (2007). Calcium signaling network in plants: an overview. Plant Signal. Behav. 2: 79-85. doi: 10.4161/psb.2.2.4176.

Uchida, S., Sohara, E., Rai, T., and Sasaki, S. (2014). Regulation of with-no-lysine kinase signaling by Kelch-like proteins. Biol. Cell 106: 45-56. doi: 10.1111/boc.201300069.

Valdivia, E.R., Chevalier, D., Sampedro, J., Taylor, I., Niederhuth, C.E., and Walker, J.C. (2012). DVL genes play a role in the coordination of socket cell recruitment and differentiation. J. Exp. Bot. 63: 1405-1412. doi: 10.1093/jxb/err378.

Viana, A.J.C., Matiolli, C.C., Newman, D.W., Vieira, J.G.P., Duarte, G.T., Martins, M.C.M., Gilbault, E., Hotta, C.T., Caldana, C., and Vincentz, M. (2021). The sugar-responsive circadian clock regulator bZIP63 modulates plant growth. New Phytol. 231: 1875-1889. doi: 10.1111/nph.17518.

Vitale, A., and Galili G. (2001). The endomembrane system and the problem of protein sorting. Plant Physiol. 125: 115-128. doi: 10.1104/pp.125.1.115.

Wang, M., Li, X., Luo, S., Fan, B., Zhu, C., and Chen, Z. (2020a) Coordination and crosstalk between autophagosome and multivesicular body pathways in plant stress responses. Cells 9: 119. doi: 10.3390/cells9010119.

Wang, X., Xu, M., Gao, C., Zeng, Y., Cui, Y., Shen, W., & Jiang, L. (2020b). The roles of endomembrane trafficking in plant abiotic stress responses. J. Integr. Plant Biol. 62: 55-69. doi: 10.1111/jipb.12895.

Wang, T.J., Wang, X.H., and Yang, Q.H. (2020c). Comparative analysis of drought-responsive transcriptome in different genotype Saccharum spontaneum L. Sugar Tech. 22: 411-427. https://doi.org/10.1007/s12355-019-00774-1.

Wang, Y., Li, Y., Zhou, F., Zhang, L., Gong, J., Cheng, C., Chen, J., and Lou, Q. (2023). Genome-wide characterization, phylogenetic and expression analysis of Histone gene family in cucumber (Cucumis sativus L.). Int. J. Biol. Macromol. 230: 123401. doi: 10.1016/j.ijbiomac.2023.123401.

Wang, W., Guo, H., Bowman, J.L., Chen, F. (2024). Plant SABATH methyltransferases: Diverse functions, unusual reaction mechanisms and complex evolution. Crit. Rev. Plant Sci. doi: 10.1080/07352689.2024.2335016.

Waseem, M., Nkurikiyimfura, O., Niyitanga, S., Jakada, B.H., Shaheen, I., and Aslam, M.M. (2022) GRAS transcription factors emerging regulator in plants growth, development, and multiple stresses. Mol, Biol. Rep. 49: 9673-9685. doi: 10.1007/s11033-022-07425-x. Epub 2022 Jun 17.

Wei, X.R., Ling, W., Ma, Y.W., Du, J.L., Cao, F.X., Chen, H.X., Chen, J.R., Li, Y.F. (2022). Genome-wide analysis of the Trehalose-6-Phosphate Synthase gene family in rose (Rosa chinensis) and differential expression under heat stress. Horticulturae 8: 429. https://doi.org/10.3390/horticulturae8050429.

Wimalasekera, R., Villar, C., Begum, T., Scherer, G.F. (2011). COPPER AMINE OXIDASE1 (CuAO1) of *Arabidopsis thaliana* contributes to abscisic acid-and polyamine-induced nitric oxide biosynthesis and abscisic acid signal transduction. Mol. Plant 4: 663-678. doi: 10.1093/mp/ssr02

Wu, C., Tang, S., Li, G., Wang, S., Fahad, S., and Ding, Y. (2019) Roles of phytohormone changes in the grain yield of rice plants exposed to heat: a review. PeerJ 7: e7792. doi: 10.7717/peerj.7792. Xiong, H., He, H., Chang, Y., Miao, B., Liu, Z., Wang, Q., Dong, F., Xiong, L. (2025). Multiple roles of NAC transcription factors in plant development and stress responses. J. Integr. Plant Biol. 67: 510-538. https://doi.org/10.1111/jipb.13854

Xu, P., Chen, H., and Cai, W. (2020). Transcription factor CDF4 promotes leaf senescence and floral organ abscission by regulating abscisic acid and reactive oxygen species pathways in Arabidopsis. EMBO Rep. 21: e48967. doi: 10.15252/embr.201948967.

Yan, S., Liu, Q., Li, W., Yan, J., and Fernie, A.R. (2022). Raffinose family oligosaccharides: crucial regulators of plant development and stress responses. Crit. Rev. Plant Sci. 41: 286-303. https://doi.org/10.1080/07352689.2022.2111756.

Yang, Y., Pian, Y., Li, J., Xu, L., Lu, Z., Dai, Y., and Li, Q. (2023). Integrative analysis of genome and transcriptome reveal the genetic basis of high temperature tolerance in *Pleurotus giganteus* (Berk. Karun & Hyde). BMC Genomics 24: 552. doi: 10.1186/s12864-023-09669-8.

Yao, X., Xiong, W., Ye, T., and Wu, Y. (2012). Overexpression of the aspartic protease ASPG1 gene confers drought avoidance in Arabidopsis. J. Exp. Bot. 63: 2579-2593. doi: 10.1093/jxb/err433. Yu, C., Yan, M., Dong, H., Luo, J., Ke, Y., Guo, A., Chen, Y., Zhang, J., and Huang, X. (2021). Maize bHLH55 functions positively in salt tolerance through modulation of AsA biosynthesis by directly regulating GDP-mannose pathway genes. Plant Sci. 302: 110676. https://doi.org/10.1016/j.plantsci.2020.110676.

Yuan, G., Qian, Y., Ren, Y., Guan, Y., Wu, X., Ge, C., and Ding, H. (2021a). The role of plant-specific VQ motif-containing proteins: an ever-thickening plot. Plant Physiol. Biochem. 159: 12-16. https://doi.org/10.1016/j.plaphy.2020.12.005.

Yuan, L., Hu, Y., Li, S., Xie, Q., and Xu, X. (2021b). PRR9 and PRR7 negatively regulate the expression of EC components under warm temperature in roots. Plant Signal Behav. 16: 1855384. doi: 10.1080/15592324.2020.1855384.

Zarreen, F., Karim, M.J., and Chakraborty, S. (2022). The diverse roles of histone 2B monoubiquitination in the life of plants. J. Exp. Bot. 73: 3854-3865. doi: 10.1093/jxb/erac120.

Zeiner, A., Francisco, A., Colina, J., Citterico, M., Wrzaczek, M. (2023) CYSTEINE-RICH RECEPTOR-LIKE PROTEIN KINASES: their evolution, structure, and roles in stress response and development. J. Exp. Bot. Volume 74: 4910-4927. https://doi.org/10.1093/jxb/erad236

Zhang, X., Ding, X., Marshall, R.S., Paez-Valencia, J., Lacey, P., Vierstra, R.D., and Otegui, M.S. (2020). Reticulon proteins modulate autophagy of the endoplasmic reticulum in maize endosperm. Elife 9: e51918. doi: 10.7554/eLife.51918.

Zhang, H., Liu, L., Li, Z., Wang, S., Huang, L., Lin, S. (2025). PLATZ transcription factors and their emerging roles in plant responses to environmental stresses. Plant Sci. 352: 112400. https://doi.org/10.1016/j.plantsci.2025.112400

Zhang, Y., Min, H., Shi, C., Xia, G., and Lai, Z. (2021). Transcriptome analysis of the role of autophagy in plant response to heat stress. PLoS ONE 16: e0247783. doi: 10.1371/journal.pone.0247783. Zhang, X., Xu, Y., and Huang, B. (2019) Lipidomic reprogramming associated with drought stress priming-enhanced heat tolerance in tall fescue (Festuca arundinacea). Plant Cell Environ. 42: 947- 958. doi: 10.1111/pce.13405.

Zhang, H., Yuan, Y., Xing, H., Xin, M., Saeed, M., Wu, Q., Wu, J., Zhuang, T., Zhang, X., Mao, L., et al. (2023). Genome-wide identification and expression analysis of the HVA22 gene family in cotton and functional analysis of GhHVA22E1D in drought and salt tolerance. Front. Plant Sci. 14: 1139526. doi: 10.3389/fpls.2023.1139526.

Zhao, J. (2011). The mechanism of the silencing of a transgene, NCED3-LUC, in Arabidopsis thaliana. KAUST Research Repository. https://doi.org/10.25781/KAUST-0J766

Zheng, X., Chen, S., Gao, C., and Zhou, J. (2024). An emerging role of non-canonical conjugation of ATG8 proteins in plant response to heat stress. Autophagy 20: 946-948. doi: 10.1080/15548627.2023.2219161

Zheng, L., Wu, H., Wang, A., Zhang, Y., Liu, Z., Ling, H.Q., Song, X.J., and Li, Y. (2023). The SOD7/DPA4-GIF1 module coordinates organ growth and iron uptake in Arabidopsis. Nat. Plants 9: 1318-1332. doi: 10.1038/s41477-023-01475-0.

Zheng, X., Zuo, Z., Yao, P., Li, X., Zhang, Q., Chen, X. (2025) Bromodomain-containing proteins interact with a non-canonical RNA polymerase II kinase to maintain gene expression upon heat stress. Nat. Plants 11: 1416–1428 (2025). https://doi.org/10.1038/s41477-025-02044-3

Zhou, H., Liu, Y., Liang, Y., Zhou, D., Li, S., Lin, S., Dong, H., and Huang, L. (2020). The function of histone lysine methylation related SET domain group proteins in plants. Protein Sci. 29: 1120- 1137. doi: 10.1002/pro.3849.

Zhou, J., and Li, Q.Q. (2023). Stress responses of plants through transcriptome plasticity by mRNA alternative polyadenylation. Mol. Hortic. 3: 19. doi: 10.1186/s43897-023-00

Table S6 Genes selected for the heat-map analysis. The heat-map analysis shown in Fig. 7 was performed using transcripts that were selected for their strong induction or repression, based on log fold-change values (LFC), in each of the three heat-shock stages, i.e., HS1 to HS11, and during the two recovery phases, i.e., R1 and R3. Another criterium for selection was their known or proposed participation in heat stress responses in plants. The table includes 30 up-regulated (in [A] ***AhHAB4-PAI-1 and*** **[C]** ***Ah2880*** OE plants), and 30 30 down-regulated-regulated (in [B] ***AhHAB4-PAI-1 and*** **[D]** ***Ah2880*** OE plants) genes, respectively

**[A] Up-regulated: *AhHAB4-PAI-1* OE**				
**HS1**	**HS5**	**HS11**	**R1**	**R3**
**AT4G32810**: Encodes a protein with similarity to carotenoid cleavage deoxygenase. LFC: + 6.47	**AT2G38390:** Peroxidase superfamily protein. LFC: + 6.60	**AT3G63350:** member of Heat Stress Transcription Factor (Hsf); LFC: + 5.28	**AT5G57520:** Encodes a zinc finger protein containing only a single finger. LFC: + 3.64	**AT3G05950:** Germin-like protein subfamily 1 member 7. LFC: + 11.58
**AT3G48510.** AtIII18x5-like protein. Feedback regulator in ABA signalling. LFC: + 6.24	**AT5G56160:** Sec14p-like phosphatidylinositol transfer family protein. LFC: + 6.17	**AT5G53680:** RNA-binding (RRM/ RBD/ RNP motifs) family protein. LFC: + 4.16	**AT1G33790:** jacalin lectin family protein. LFC: + 2.20	**AT4G32950:** Protein phosphatase 2C family protein. LFC: + 9.36
**AT3G25290:** Auxin-responsive family protein 1 LFC: + 1.99	**AT1G77200:** encodes a member of the DREB subfamily A-4 of ERF TFs. LFC: + 3.09	**AT2G01008**: maternal effect embryo arrest protein. LFC: + 3.60	**AT2G41550:** Rho termination factor. LFC: + 1.83	**AT3G04320:** Kunitz family trypsin and protease inhibitor protein. LFC: + 7.71
**[B] Down-regulated: *AhHAB4-PAI-1* OE**				
**HS1**	**HS5**	**HS11**	**R1**	**R3**
**AT4G18450:** encodes a member of the ERF (ethylene response factor. LFC: -3.47	**AT2G41415:** Encodes a Maternally expressed gene (MEG) family protein. LFC: - 6.37	**AT5G57340:** Ras guanine nucleotide exchange factor Q-like prot: LFC: -9.56	**AT4G11911:** STAY-GREEN-like protein. LFC: -6.73	**AT5G21100:** Plant L-ascorbate oxidase. LFC: - 10.85
**AT4G32208:** heat shock protein 70 (Hsp 70) family protein. LFC: -2.51	**AT1G66850:** Bifunctional inhibitor/lipid-transfer protein/seed prot. LFC: - 4.18	**AT1G79890:** RAD3-like DNA-binding helicase protein. LFC: -7.60	**AT5G22430:** Pollen Ole e 1 allergen and extensin family protein. LFC: - 1.78	**AT3G56290:** Potassium transporter. LFC: -10.24
**AT1G72416:** Chaperone DnaJ-domain superfamily protein. LFC: -1.62	**AT4G15430:** ERD (early-responsive to dehydration stress) family. LFC: -3.70	**AT5G37300:** Encodes a bifunctional enzyme, wax ester synthase. LFC: -7.40	**AT3G47480:** Calcium-binding EF-hand family protein. LFC: -1.76	**AT1G69730:** Wall-associated kinase family protein. LFC: -9.56
**[C] Up-regulated: *Ah2880* OE**				
**HS1**	**HS5**	**HS11**	**R1**	**R3**
**AT1G01020:** ARV1 family protein. LFC: 1.02	**AT1G16120:** Encodes a WAK-like receptor-like kinase: LFC: 5.73	**AT4G21870:** HSP20-like chaperone. LFC: 3.37	**AT5G44680:** DNA glycosylase superfamily protein LFC: 8.80	**AT4G05200:** Encodes a cysteine-rich receptor-like protein kinase. LFC: 4.93
**AT1G02460:** Pectin lyase-like superfamily protein. LFC: 1.35	**AT4G11660:** member of Heat Stress Transcription Factor (Hsf). LFC: 2.30	**AT4G12735:** Encodes a peroxisomal protein. LFC: 2.26	**AT4G21020:** Late embryogenesis abundant protein (LEA) family protein LFC: 7.12	**AT1G16150:** Encodes a WAK-like receptor-like kinase LFC: 4.1
**AT5G66550**: Maf-like protein. LFC: 1.43	**AT2G34450:** HMG-box (high mobility group) DNA-binding family protein. LFC: 1.97	**AT1G31240:** Bromodomain transcription factor. LFC: 1.49	**AT2G24440:** selenium binding protein. LFC: 6.79	**AT5G62350:** Plant invertase/ pectin methylesterase inhibitor. LFC: 2.51

[D] Down-regulated: *Ah2880* OE				
**HS1**	**HS5**	**HS11**	**R1**	**R3**
**AT1G66960:** Terpenoid cyclase family protein: LFC: -9.27	**AT3G59845:** Zinc-binding dehydrogenase family protein. LFC: -6.83	**AT1G27710:** Glycine-rich protein family. LFC: -7.30	**AT5G48430:** Eukaryotic aspartyl protease family protein. LFC: - 8.60	**AT1G16720:** Encodes HCF173 protein. LFC: -9.82
**AT1G66850:** Bifunctional inhibitor/lipid-transfer protein/seed. LFC: -6.12	**AT1G55450:** S-adenosyl-L-methionine-dependent methyltransferase. LFC: -2.18	**AT1G11670:** MATE efflux family protein. LFC: -5.67	**AT4G28420:** Tyrosine transaminase family protein . LFC: - 6.68	**AT2G36630:** Sulfite exporter TauE/SafE family protein. LFC: -9.71
**AT5G22430:** Pollen Ole e 1 allergen and extensin family protein. LFC: -2.19	**AT2G30010:** Encodes a member of the TBL (TRICHOME BIREFRINGENCE. LFC: -2.05	**AT5G28320:** embryo defective protein. LFC: -2.45	**AT1G30760:** Encodes a BBE-like enzyme that acts in monolignol BS. LFC: - 5.05	**AT1G07700:** Thioredoxin superfamily protein. LFC: -8.90

Supplemental experimental procedures (SEP)

Sequencing and mapping to reference genome

The transcriptomic (RNA-Seq) experiment consisted in extracting total RNA from leaves of *A. thaliana* of the two OE plant lines or WT subjected to the different HS or HS + R treatments, as detailed in the main text. A total of 30 samples were shipped to Novogene (https://en.novogene.com/; accessed on: 26 July 2024) for paired-end sequencing with individual reads of 150 bp, giving a maximum transcriptome coverage of 300 bp per paired read. The full report from Novogene, containing details for each one of the 30 libraries is available upon request.

Sample	Raw reads	Raw data	Effective(%)	Error(%)	Q20(%)	Q30(%)	GC(%)
L1201D2	50333814	7.6	98.68	0.03	97.46	92.92	46.70
WT03D1	44977656	6.7	98.58	0.03	97.65	93.44	50.33
WT03D2	48841868	7.3	98.67	0.03	97.66	93.51	50.22
L1203D1	65428582	9.8	98.83	0.03	97.59	93.29	47.08
L1203D2	62004640	9.3	98.80	0.03	97.57	93.25	47.02
L1301H1	47173204	7.1	98.06	0.03	97.87	93.83	46.14
L1301H2	44326632	6.6	97.76	0.03	97.79	93.64	46.30
L1305H1	43723916	6.6	97.60	0.03	97.67	93.37	45.88
L1305H2	48204882	7.2	98.15	0.03	97.75	93.58	46.37
L1311H1	50461720	7.6	98.24	0.03	97.82	93.71	45.74
L1311H2	62755036	9.4	98.10	0.03	97.72	93.46	45.54
L1301D1	47443980	7.1	98.68	0.03	97.73	93.47	45.69
L1301D2	47502994	7.1	98.78	0.03	97.69	93.40	45.71
L1303D1	48516542	7.3	99.06	0.03	97.60	93.18	46.33
L1201H1	43616212	6.5	98.75	0.03	97.61	93.06	46.98
L1201H2	40607604	6.1	98.10	0.03	97.64	93.29	46.90
WT05H1	43942852	6.6	98.65	0.03	97.78	93.60	46.26
WT05H2	51653024	7.7	98.70	0.03	97.66	93.33	46.26
L1205H1	42350988	6.4	98.99	0.03	97.65	93.34	47.10
L1205H2	48258470	7.2	98.81	0.03	97.42	92.78	46.91
WT11H1	45724076	6.9	98.27	0.03	97.63	93.24	46.43
L1211H2	48305030	7.2	98.22	0.03	97.71	93.50	46.02
WT01D1	44390948	6.7	99.20	0.03	97.63	93.26	45.38
WT01D2	50919410	7.6	98.91	0.03	97.21	92.26	45.37
L1201D1	51970172	7.8	99.04	0.03	97.61	93.26	46.52
WT01H1	45138530	6.8	98.57	0.03	97.72	93.49	46.97
WT01H2	45879144	6.9	98.96	0.03	97.65	93.23	46.95
L1303D2	40347246	6.1	98.72	0.03	97.69	93.39	46.59
WT11H2	48617582	7.3	98.47	0.03	97.13	92.17	47.30
L1211H1	44806252	6.7	98.80	0.03	96.83	91.53	46.32

Figure SEP1 Data Quality Summary. Sample: sample name; Raw reads: total amount of paired reads of raw data, each four lines taken as one unit (it equals the amount of read1 and read2 from each pair); Raw data: (Raw reads) × (sequence length). PE150, sequencing length equals 150; Effective: (Clean reads/Raw reads)*100%; Error: base error rate; Q20, Q30: (Base count of Phred value > 20 or 30) / (Total base count); GC: (G & C base count) / (Total base count)

Table SEP1 presents the description of the distinct RNA-Seq replicated libraries employed in the experiment (column "Description"), as well as the total number of reads for both replicates of the libraries that were uniquely mapped to the *A. thaliana* reference genome (Total number of Mapped Reads; TMR). In Table SEP1 rows 1 to 6 include libraries constructed from plants which received the Heat Shock –always for 22 hours, plus Recovery of 1 or 3 days, say "HS + R", while rows 7 to 15 present libraries constructed from plants which received "HS" by 1, 5.5 or 11 hours (time shown between parenthesis in all cases; see main text for details). Table SEP1 shows that we have an RNA-Seq experiment which includes two factors, say, the overexpressing gene (column "OE") at levels "Ah2880 ", "AhHAB4-PAI-1 " or "None" for lines "L12", "L13" and "WT" respectively, and the factor in column "Description", which include Heat Shock ("HS") only or "HS" plus Recovery, "HS + R". Within "HS + R" (rows 1 to 6) we have a complete factorial with 3 × 2 = 6 combinations of each "OE" varying time of recovery at levels 1 or 3 days. Likewise, in rows 7 to 15 we have a complete factorial, with the three levels of column "OE" combined with 3 different times of Recovery, "R", which are 1, 5.5 and 11 hours, to give a total of 3 × 3 = 9 libraries. This complex experimental structure allowed to obtain relevant results via Differential Gene Expression (DGE) coupled with Gene Ontology (GO) enrichment analyses.

Table SEP1. RNA-Seq replicated libraries and Total number of Mapped Reads (TMR).

Row	Library	Line	OE	Description	TMR
1	L1201D	L12	*Ah2880*	HS (22 h) + R 1 day.	1,731,380
2	L1203D	L12	*Ah2880*	HS (22 h) + R 3 days.	2,801,740
3	L1301D	L13	*AhHAB4-PAI-1*	HS (22 h) + R 1 day.	5,200,842
4	L1303D	L13	*AhHAB4-PAI-1*	HS (22 h) + R 3 days.	8,615,792
5	WT01D	WT	None	HS (22 h) + R 1 day.	6,238,170
6	WT03D	WT	None	HS (22 h) + R 3 days.	* 97,502
7	L1201H	L12	*Ah2880*	HS (1 h).	6,632,848
8	L1205H	L12	*Ah2880*	HS (5.5 h).	6,659,708
9	L1211H	L12	*Ah2880*	HS (11 h).	3,593,116
10	L1301H	L13	*AhHAB4-PAI-1*	HS (1 h).	3,579,162
11	L1305H	L13	*AhHAB4-PAI-1*	HS (5.5 h).	3,360,548
12	L1311H	L13	*AhHAB4-PAI-1*	HS (11 h).	9,211,332
13	WT01H	WT	None	HS (1 h).	9,589,024
14	WT05H	WT	None	HS (5.5 h).	9,104,808
15	WT11H	WT	None	HS (11 h).	3,295,544

Notes:
Column “Library” gives the main part of the library name. Each one of those 15 libraries has a biological replicate (1 and 2 at the end of the library name in column “Sample” of Figure 2) for a total of 30 libraries. Column “OE” gives the overexpressing gene: *Ah2880* in L12,
AhHAB4-PAI-1 in L13 or None in WT.
Column “Description” includes the time of Heat Shock (“HS”) between parenthesis in hours plus the time of recovery, “R”, if any.

The total number of mapped reads –column “TMR” in Table SEP1, varied between a minimum of only 97,502 for library “WT03D” up to a maximum of 9,589,024 for library “WT01H”, having a median of 5,200,842 and a mean of approximately 5,314,101 clean reads mapped to the reference genome in the set of 15 libraries. It is worth noticing the very large ratio between the maximum and minimum of the TMR values, which is 9,589,024 / 97,502 ≈ 98. That very large difference in the values of TMR was due to the fact that wild type plants used to construct the “WT03D” library were almost dead or dying after three days of receiving the HS for 22 h, and thus the integrity of the RNA from those plants was poor, explaining the low number (97,502) of TMR. This suggestion is also backed up by the fact that the two replicates of this library, say “WT03D1” and “WT03D2”, gave the largest values of GC (%), 50.33 and 50.22, within the 30 libraries sequenced (see Fig. SEP1), meaning that libraries “WT03D” were biased in favor of transcripts with high GC content.
To evaluate the consistency achieved in measuring gene expression in our experiment, we evaluated the correlation between pairs of libraries with different treatments and within the two replicates of the same library (DeLuca et al. 2012). Given that we have 30 samples (Fig. SEP 1), representing 15 different libraries (Table SEP1), we estimated the (30 × (30 − 1))/2 = 435 Pearson’s correlation coefficients between pairs of samples containing the relative frequencies of expression for all genes. Of these 435 pairs of correlations, 15 are correlations within replicates, while the remaining 420 are correlations between treatments with different combinations of factors. Table SEP2 presents the results of these correlation analyses.

Table SEP2. Correlation analyses of read counts in libraries between and within the same combinations of factors.

				95% C $\hat{r}$:	
Source	*n* pairs	$\hat{r}$	$\hat{r}^2$	*LL*	*UL*
Between treatments	420	0.69841	0.51952	0.68130	0.71552
Within replicates	15	0.98916	0.97853	0.98387	0.99446

95% CI $\hat{r}$: 95% Confidence Interval for mean $\hat{r}$ (Lower and Upper limits).

In Table SEP2 we can see the large and significant (*p*-value $4.6 \times 10^{-114} \approx 0$ in the t-test) difference in the correlations between samples subjected to the same combinations of factors (row “Within replicates”), which has a mean $\hat{r} = 0.98916$, and libraries subjected to the different combinations of factors (row “Between treatment”), which has a mean $\hat{r} = 0.69841$ In summary, from Table SEP2 we conclude that the statistical noise (unexplained variation within replicates) is much smaller that the differences in expression among the treatments and thus Differential Gene Expression Analyses (DEA) are likely to give robust estimates.

1. Differential Gene Expression (DEG) and data curation

All statistical analyses were performed in R (R Core Team 2013), version 4.3.2 (2023-10-31). Differential Gene Analyses (DGA) were performed within R using the package "edgeR" (version 3.40.0) (Robinson et al. 2010). Gene Ontology (GO) (Gene Ontology Consortium, 2004) enrichment analyses were per- formed in situ between groups of genes of interest. In each step of the process *p*-values from the statistical test where transformed into *q*- values using the algorithm presented in (Benjamini and Hochberg 1995) implemented in the R function "p.adjust(p, method="fdr")", and only results with $q < 0.01$ were labeled as significant to obtain a False Discover Rate (FDR) of 1%.

As a first step all contrast between duplicated libraries in column "Library" of Table SEP1 were performed. Given that we have 15 libraries (different treatments) a total of $15(15-1)/2 = 105$ contrasts between all different pairs of libraries were performed and only genes with a FDR of 1% were taken into account for further analyses.

Using the package edgeR for all 105 pairs of contrasts and filtering the results by a FDR of 1%, we obtained a total of 786,975 significant results. Those results presented the same gene as significant in one or more contrasts; given that a total of 28,253 genes presented detectable expression in one or more libraries, the redundancy of the results was 786,975/28,253 ≈ 28, i.e., a proportion of approximately 28% of the significant genes appeared in more than one of the 105 contrasts performed. The number of Differentially Expressed Genes (DEG) at FDR 1% in all the 105 contrasts varied per contrast between a minimum of 13 up to a maximum of 14,373 with a mean of 7,495, a median of 8,038 and a standard deviation of 3,686. The contrast with the minimum number of DEG (only 13 genes), is "L1201D *vs* L1203D", that is, the contrast within the line L12 in recovery at recovery times 1 and 3 days. On the other hand, the contrast with the highest number of DEG, 14,373, corresponds to contrast "WT01H *vs* WT01D", that is, the contrast between the line WT at 1 h of stress with the same line (WT) but in the recovery treatment after 1 day.

Given the impossibility of making sense of such a large number of significant results in such a large number of different contrasts, we designed a strategy to decrease the complexity of the interpretation.

First, we performed a GO enrichment analysis in all the 105 contrasts, and isolated all Transcription Factors (TF) which were annotated in the GO biological process (BP) "*water deprivation*" (GO:0009414). This search resulted in a total of 61 different TF that were differentially expressed in one or more of the contrasts.

To organize these 61 TF, we determined in which one of the 15 libraries those genes presented their maximum of standardized expression. This criterion univocally classifies each one of those 61 TF by the library at which the maximum standardized expression of the TF was reached. Table 3 presents the 13 groups obtained for the 61 TF's with this procedure.

In Table SEP3 column "Group" (1 to 13) defines the number of group assigned to each one of the 61 TF, and that group number is used in further tables and figures. Column "n(TF)" gives the number of TF which are included in each group, and the table is ordered in decreasing order of that value. Column "Library" gives the library at which the maximum standardized expression was found for the TF in column "Loci".

Thirteen of the fifteen different libraries appear in column "Library" in Table SEP3. Libraries "WT01H" and "L1301D" are absent from that column because none of the 61 TF's have a maximum of standardized expression at those two libraries. Column "$\bar{x}$" in Table 3 gives the average (over the number of TF's in the group) of the maximum standardized expression, while column "$\hat{S}$" gives the standard deviation for those maxima. Finally, column "Loci" gives the identifiers of the TF's in each one of the 13 groups.

It is interesting that the TF AT3G02160 –which appear in red in the group 2 in Table 3, was also detected by the protein-protein interaction in this work (unpublished data). That TF is identified simply as "Bromodomain transcription factor" and details for that gene can be consulted in AT3G02160 in KEGG.

Table SEP3. Definition of groups in which the 61 TF's were classified by having the standardized maximum expression in a given library (column "Library").

Group	n(TF)	Library	$\bar{x}$	$\hat{S}$	Loci:
1	14	WT03D	2.80	0.65	AT1G22190, AT1G46768, AT1G71130, AT1G76590, AT1G78080, AT2G38470, AT2G47950, AT3G15500, AT3G19290, AT3G49690, AT3G62990, AT4G24020, AT5G13330, A T5G61590
2	8	L1201D	2.05	0.21	AT1G02220, AT1G43160, AT1G52890, AT1G69310, AT2G38340, AT2G47190, AT3G02160, AT3G58710
3	8	L1311H	2.11	0.37	AT1G51140, AT2G41070, AT3G47600, AT3G50310, AT4G06634, AT4G25480, AT4G27410, AT4G34000
4	6	L1303D	2.39	0.48	AT1G08810, AT1G74430, AT4G14540, AT5G07690, AT5G07700, AT5G21960
5	5	WT01D	2.06	0.55	AT1G19210, AT2G36270, AT2G38880, AT2G46680, AT4G16750
6	4	L1205H	1.94	0.47	AT1G01250, AT3G20310, AT4G25490, AT5G67190
7	3	L1211H	1.82	0.41	AT2G23340, AT5G05410, AT5G11270
8	3	L1301H	2.03	0.19	AT1G19490, AT1G54160, AT5G62470
9	3	WT11H	3.18	0.31	AT1G22985, AT2G47520, AT3G11020
10	2	L1201H	1.71	0.48	AT1G15360, AT1G17950
11	2	L1203D	2.34	0.20	AT1G28520, AT2G41240
12	2	WT05H	2.45	0.78	AT1G45249, AT1G77200
13	1	L1305H	3.31	–	AT1G12610

Tables SEP4 to SEP8 give the descriptions of each one of the 61 TF's, and in each case the locus identifier has a link to the TAIR site (loci are in blue in those tables).

Figures SEP2 to SEP4 present the plots of the Standardized Expression for the TF's × the Coded Time after Heath Shock for each one of the TF's in each one of the 13 groups, respectively. In these plots small random noise was added to the value in the *X*-axis (coded time after heath shock) to decrease the overlapping of the symbols and improve interpretability. Also, in those figures each point represents a single TF and shape and color determine in which of the lines the TF was expressed (WT – black circles; L12 – blue triangles and L13 – red squares). The mean standardized expression of the "n(TF)" per group and coded time are presented as purple asterisks linked with a purple line between neighboring times.

Finally, results in Tables SEP4 to SEP8 in conjunction with Figures SEP2 to SEP4 in this document were carefully analyzed to obtain **Tables S 3, S4 and S5** listed above. The headers of those main text tables are:

Table S3. Early transcriptional responses (1 and 5 h) to heat shock in *AhHAB4-PAI-1* and *Ah2880* overexpressing *A. thaliana plants.*

Table S4. Intermediate transcriptional responses (11 h) to heat shock in *AhHAB4-PAI-1* and *Ah2880* overexpressing *A. thaliana* plants.

Table S5. Transcriptional responses during recovery (1 and 3 days-post-HS) in *AhHAB4-PAI-1* and *Ah2880* overexpressing *A. thaliana* plants.

In those **Tables**, the groups of TF are identified as in Table SEP3 of this supplementary material, which are also linked with the corresponding plot of standardized expression in Figures SEP2 to SEP14.

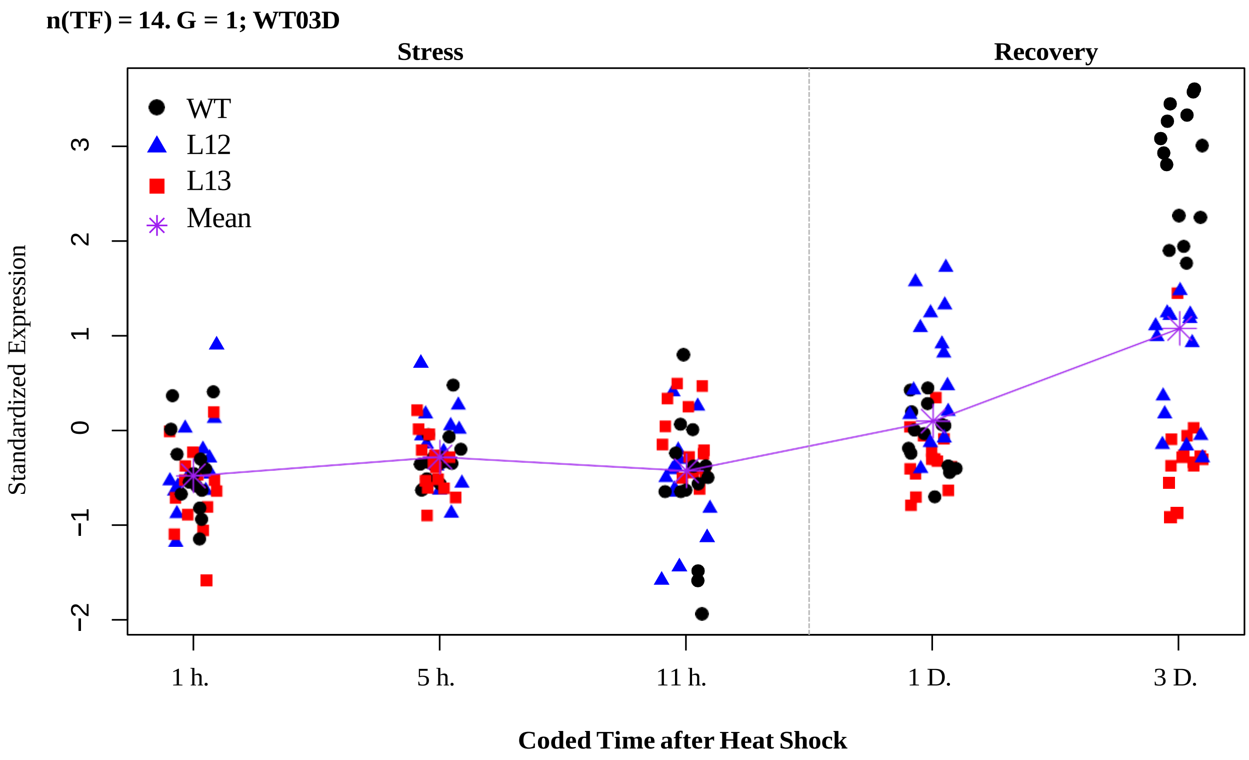

Figure SEP2 Group 1 (G = 1 in Table SEP4)

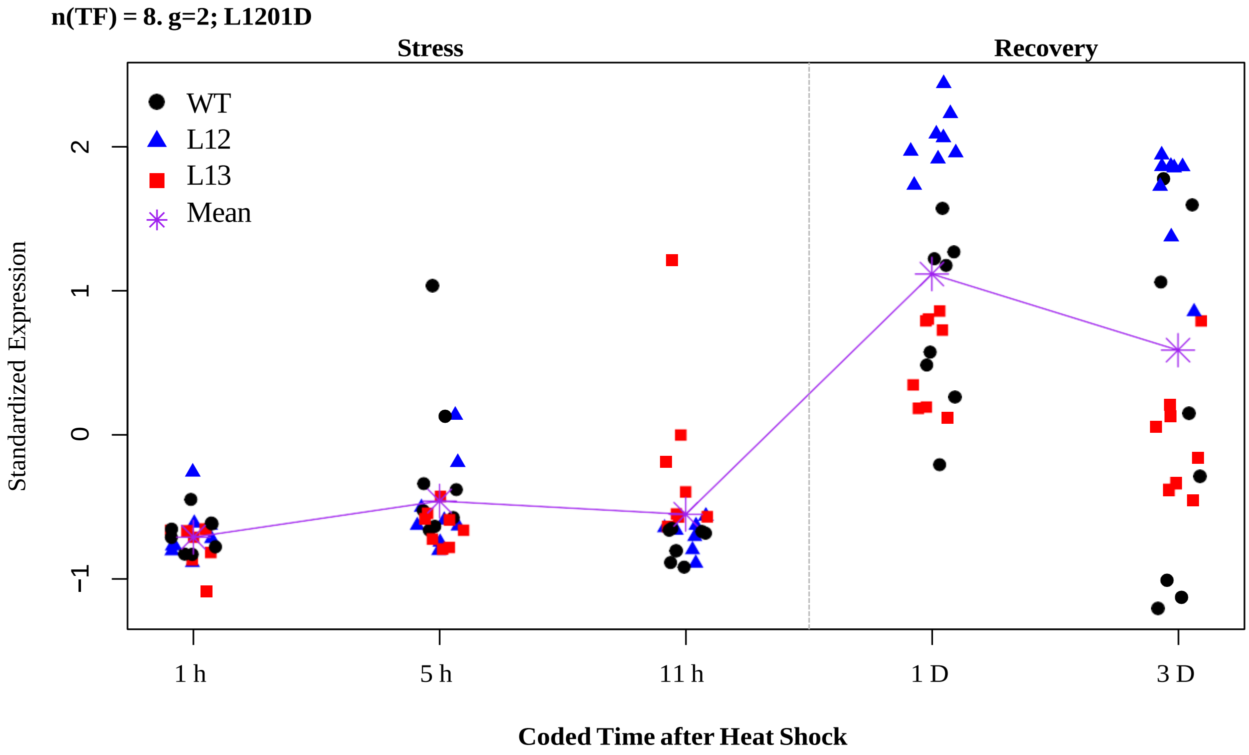

Figure SEP3. Group 2 (G = 2 in Table SEP5)

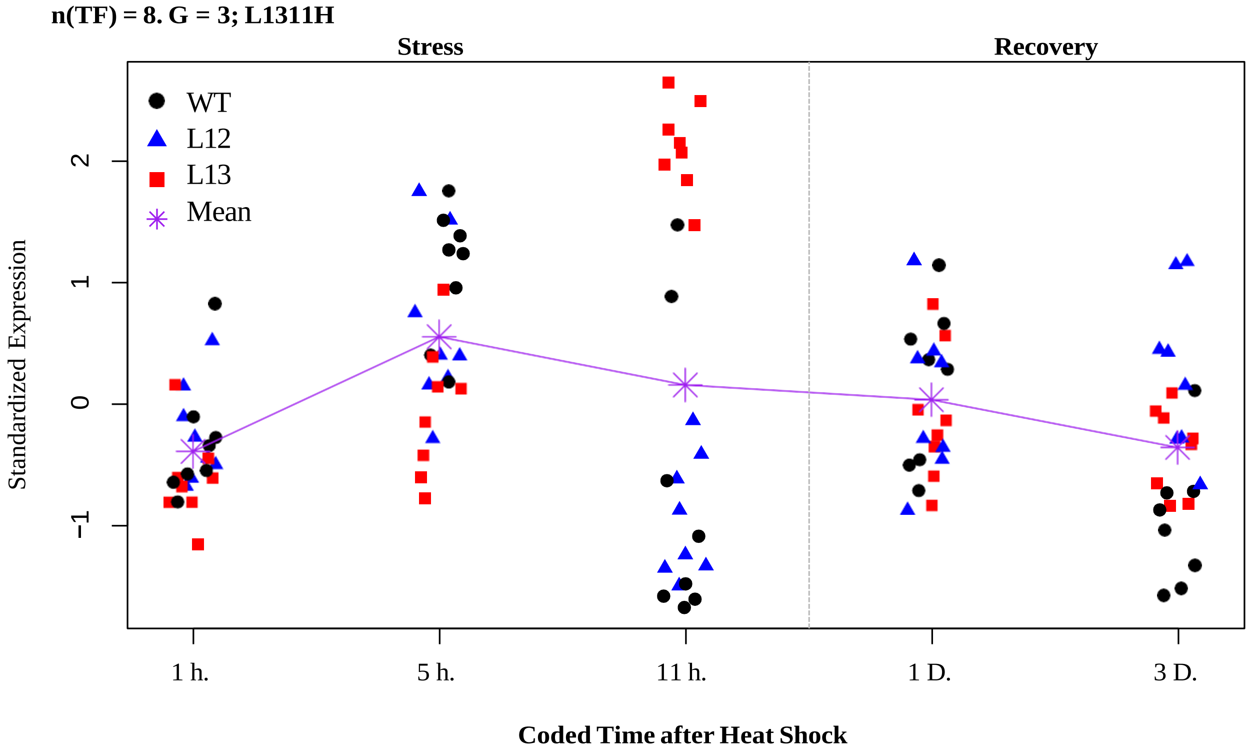

Figure SEP4 Group 3 (G = 3 in Table SEP5)

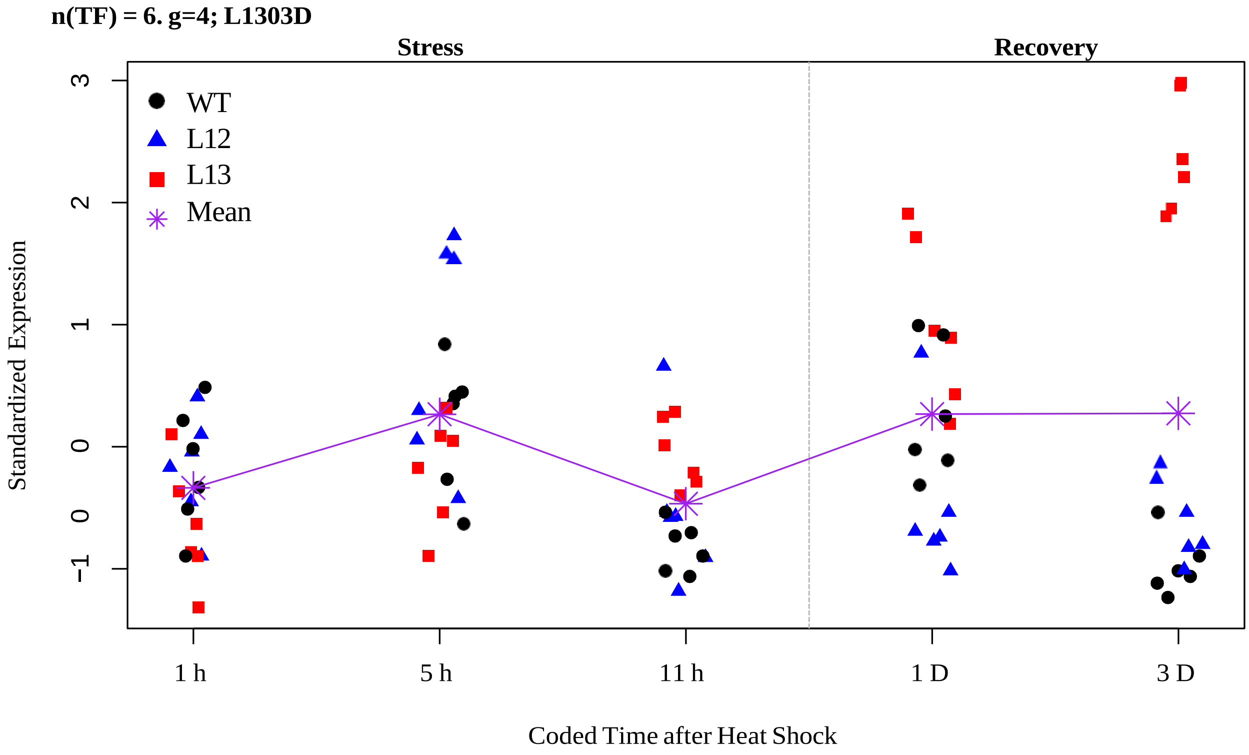

Figure SEP5 Group 4 (G = 4 in Table SEP6)

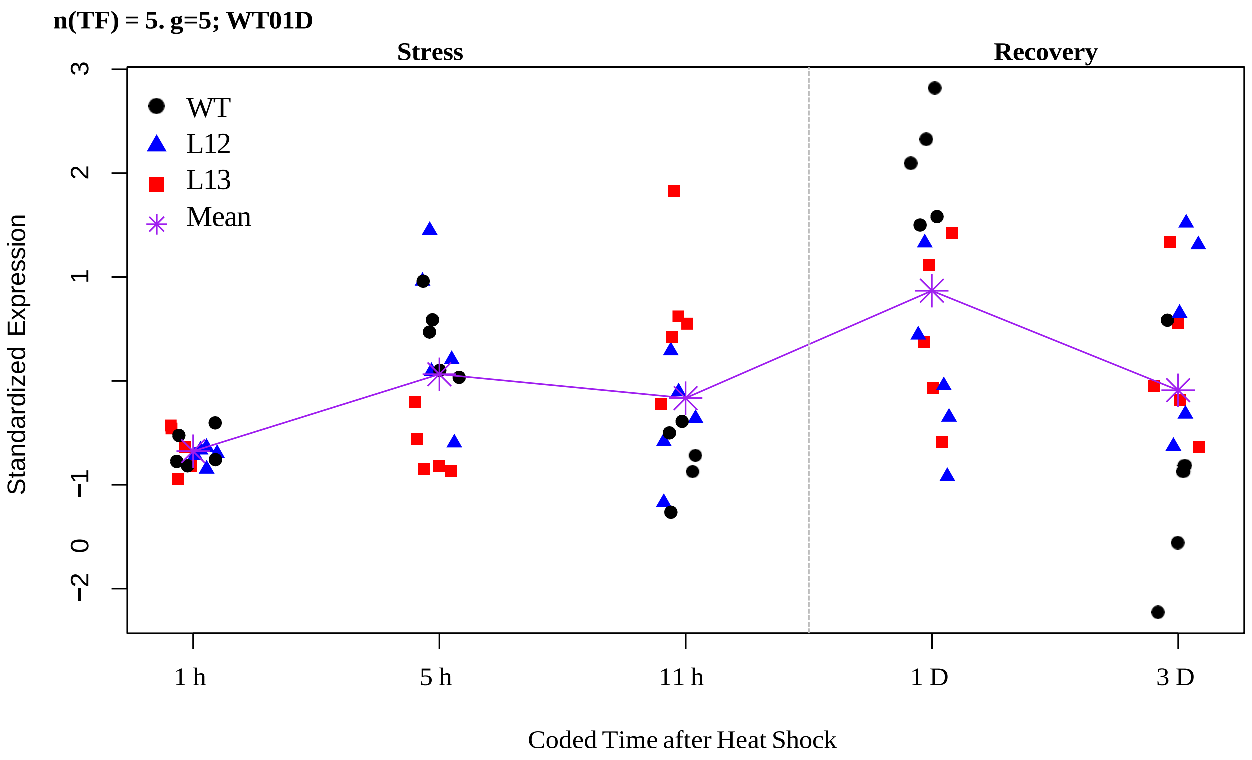

Figure SEP6 Group 5 (G = 5 in Table SEP6)

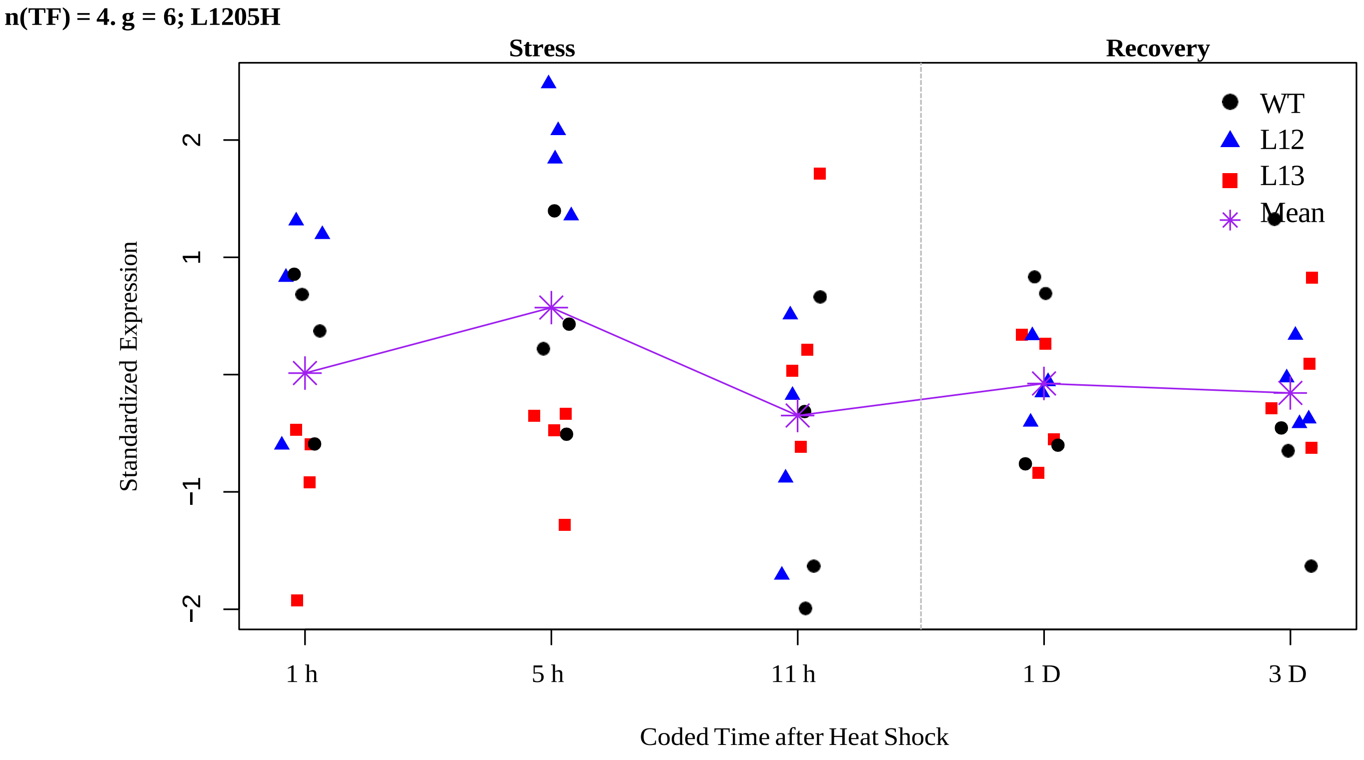

Figure SEP7 Group 6 (G = 6 in Table SEP6).

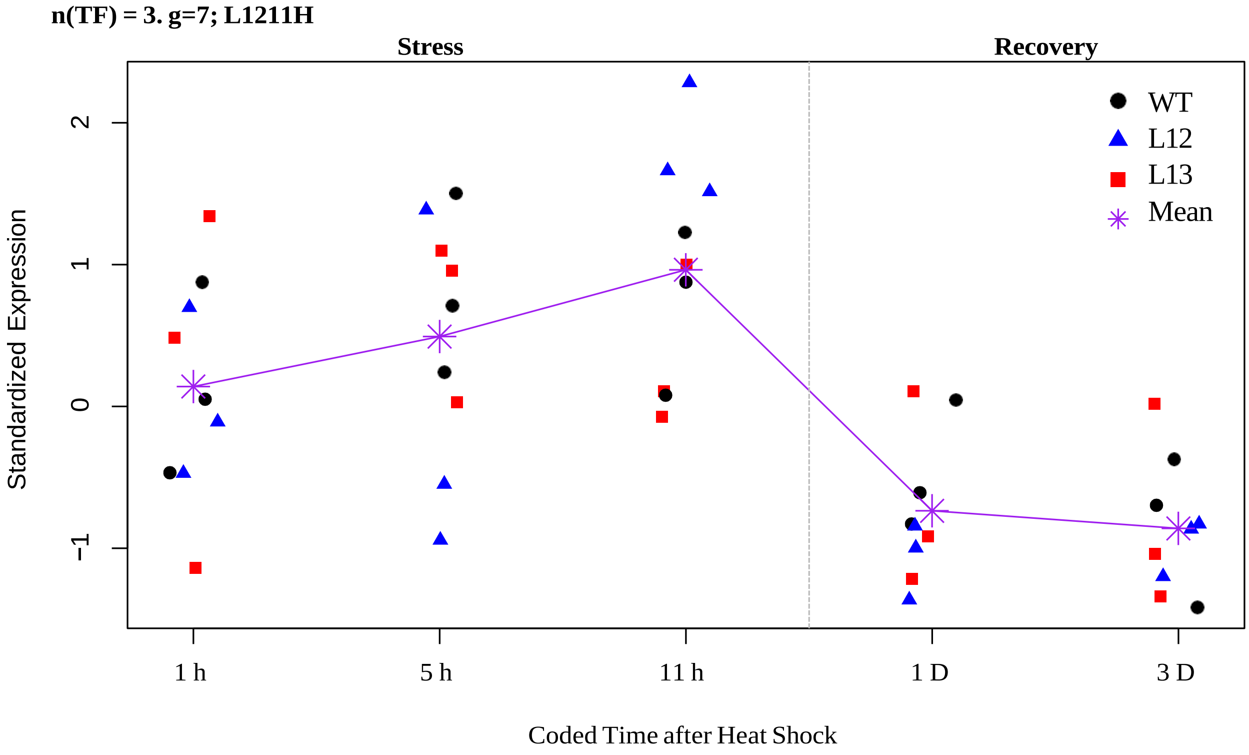

Figure SEP8 Group 7 (G = 7 in Table SEP7)

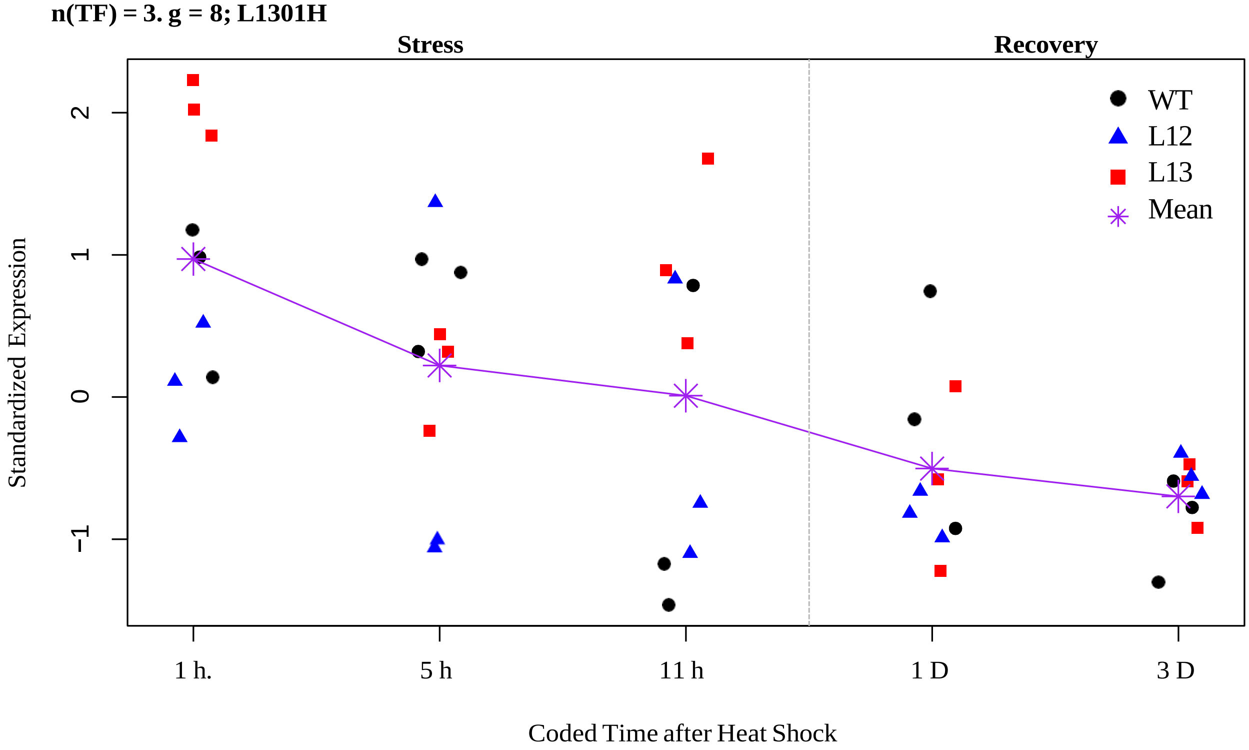

Figure SEP9 Group 8 (G = 8 in Table SEP7).

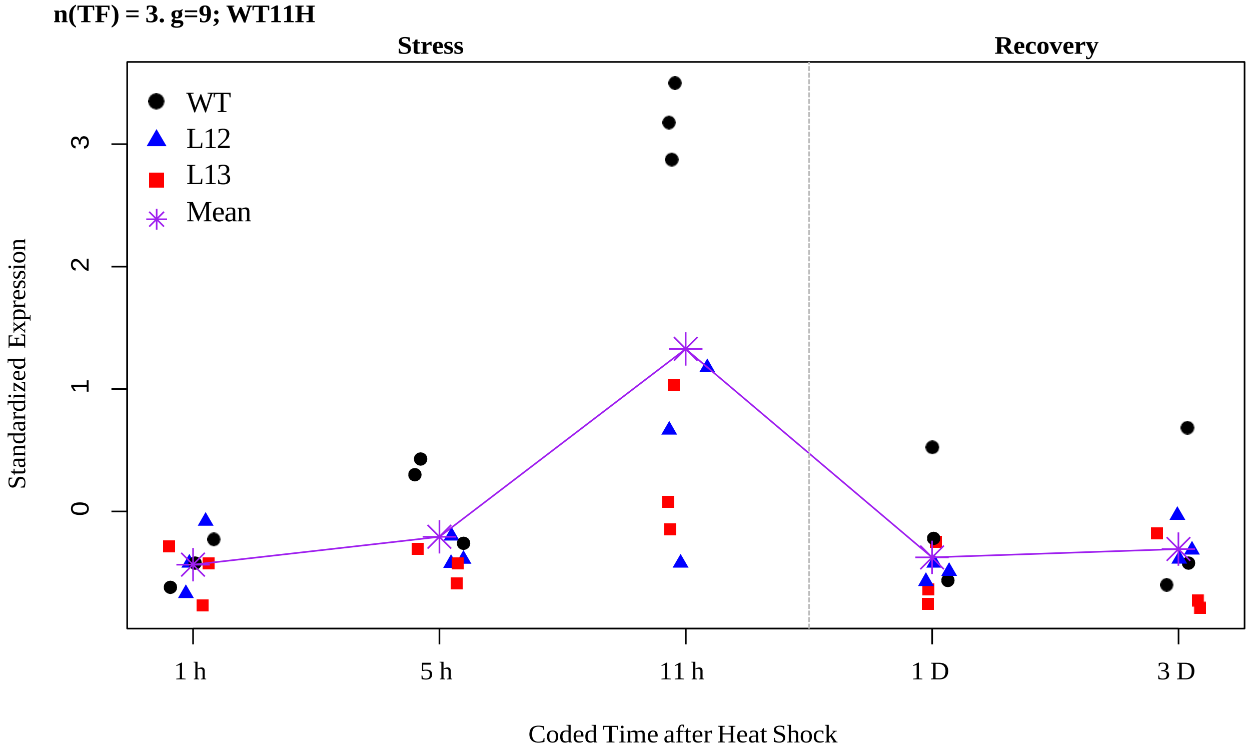

Figure SEP10 Group 9 (G = 9 in Table SEP7)

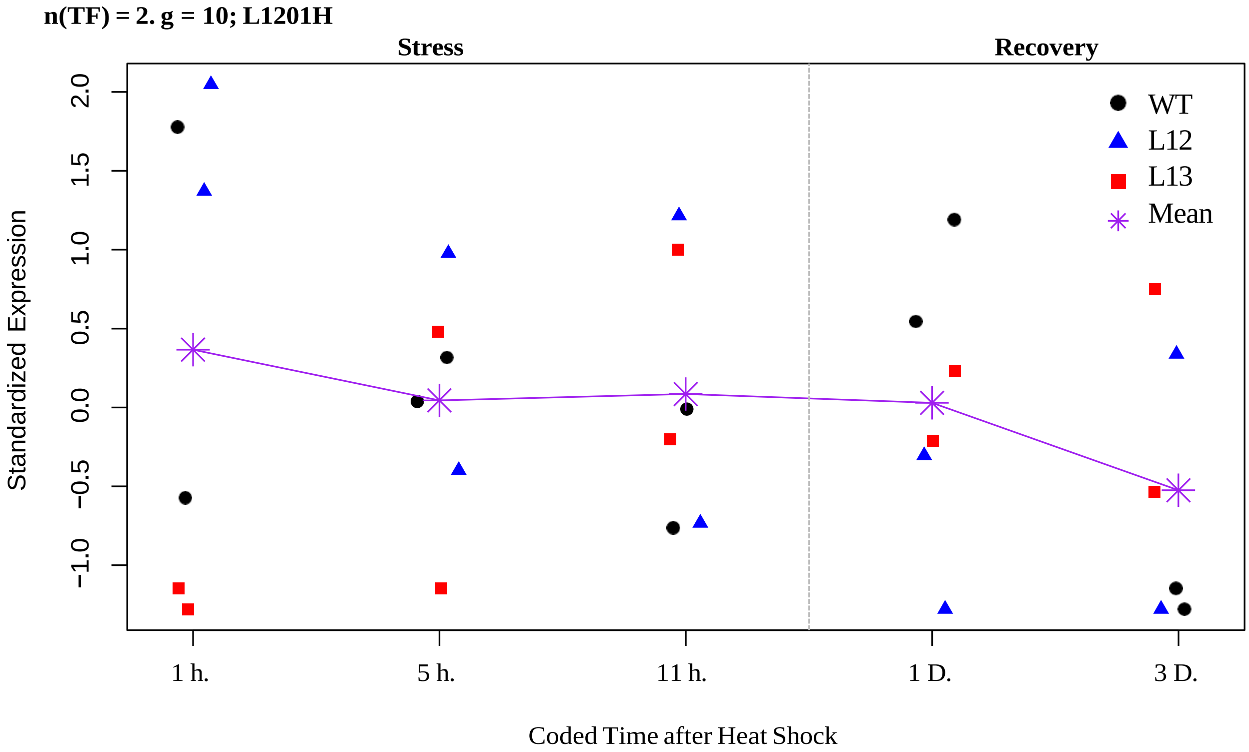

Figure SEP11 Group 10 (G = 10 in Table SEP7)

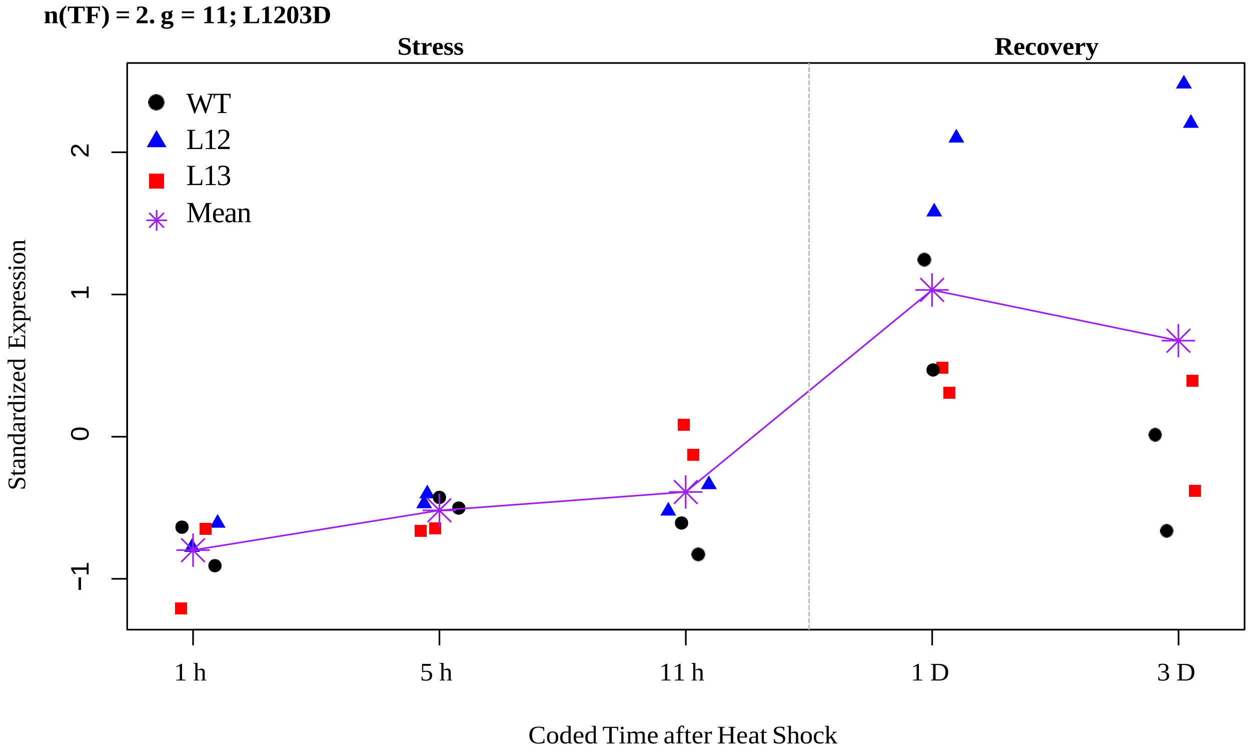

Figure SEP12 Group 11 (G = 11 in Table SEP8)

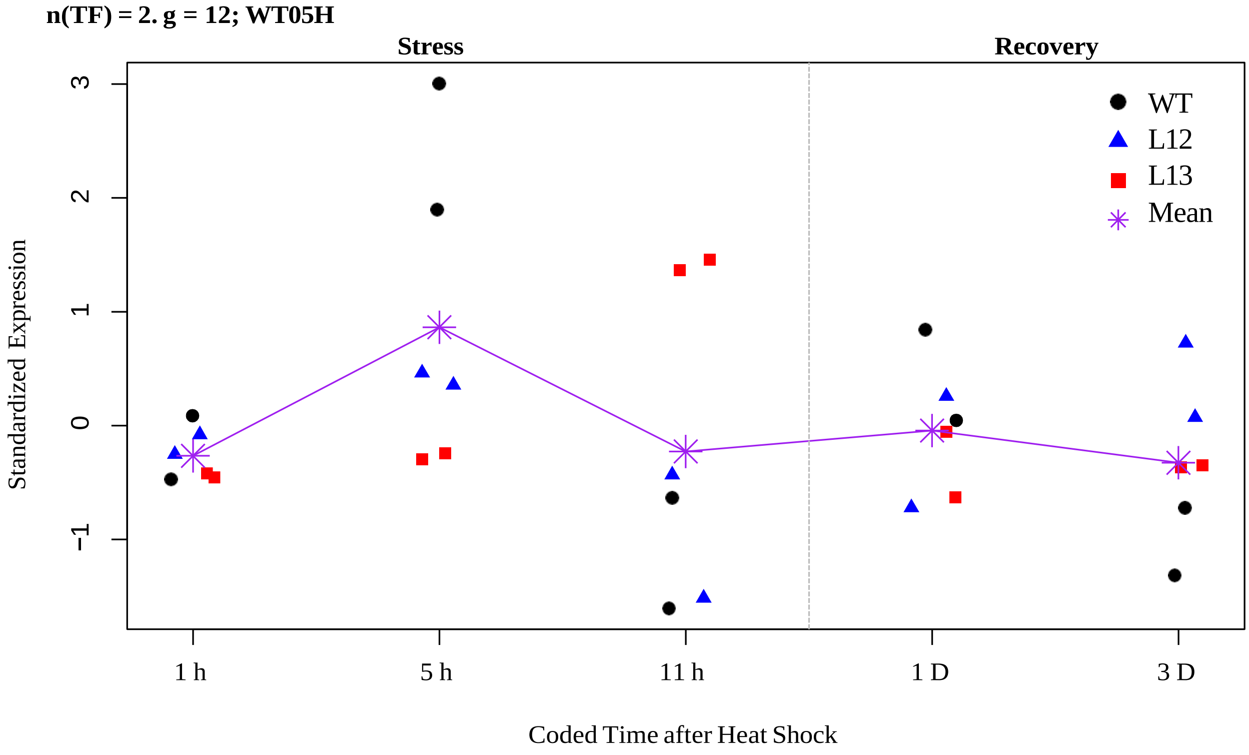

Figure SEP13 Group 12 (G = 12 in Table SEP8)

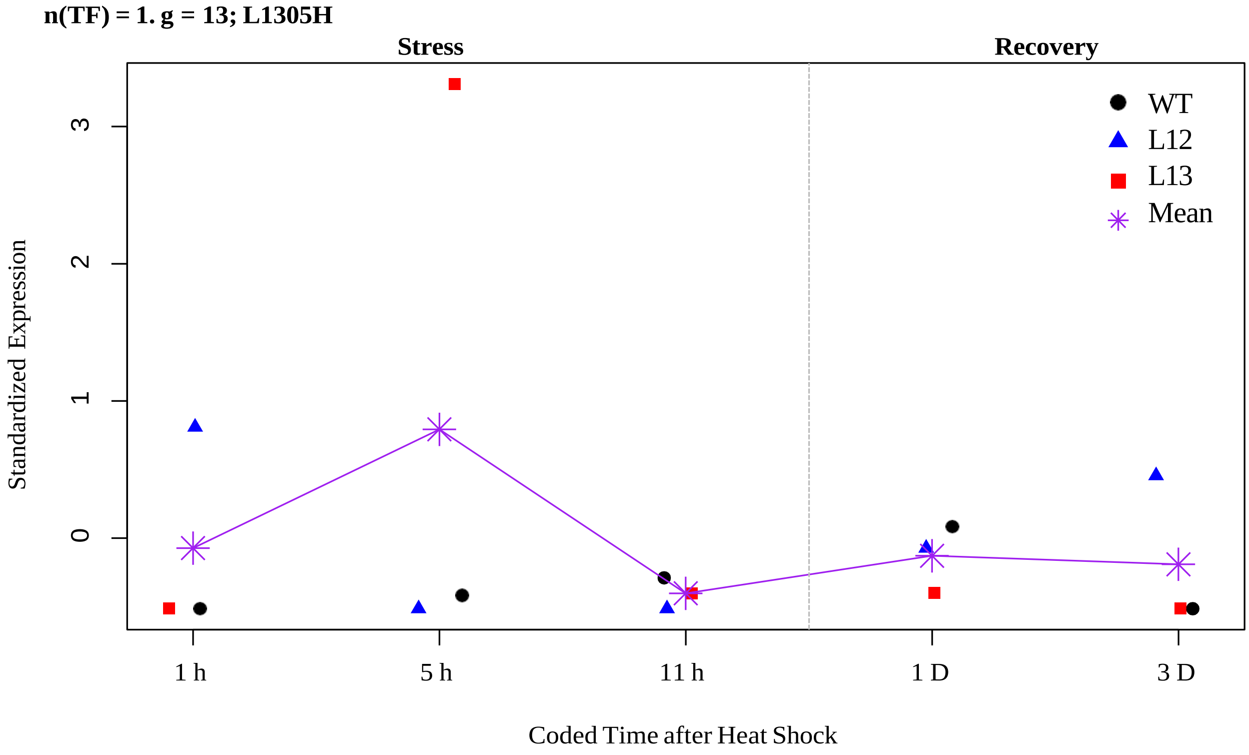

Figure SEP14 Group 13 (G = 13 in Table SEP8)

Table SEP4 TF's in Group (G) 1. n(TF) = 14. G = 1; WT03D

Locus	G	Description
AT3G62990	1	myelin transcription factor-like protein;
AT2G47950	1	myelin transcription factor-like protein;
AT1G76590	1	PLATZ transcription factor family protein;
AT3G19290	1	bZIP transcription factor with specificity for abscisic acid-responsive elements (ABRE). Mediates ABA-dependent stress responses.ABF4 acts through SnRK2 pathway and binds to ABA response elements of the promoters of NYE1 and regulates their expression to promote chlorophyll degradation.
AT1G71130	1	encodes a member of the ERF (ethylene response factor) subfamily B-5 of ERF/AP2 transcription factor family. The protein contains one AP2 domain. There are 7 members in this subfamily.
AT5G61590	1	Encodes an AP2/ERF-type transcription factor that is preferentially expressed in the epidermis and induced by darkness and negatively regulates cuticular wax biosynthesis.
AT3G49690	1	Putative homolog of the Blind gene in tomato. Together with RAX1 and RAX3 belong to the class R2R3 MYB genes; encoded by the Myb-like transcription factor MYB84, regulates axillary meristem formation.
AT3G15500	1	Encodes an ATAF-like NAC-domain transcription factor that doesn't contain C-terminal sequences shared by CUC1, CUC2 and NAM. Note: this protein (AtNAC3) is not to be confused with the protein encoded by locus AT3G29035, which, on occasion, has also been referred to as AtNAC3. The mRNA is cell-to-cell mobile.
AT4G24020	1	Encodes NIN Like Protein 7 (NLP7). Modulates nitrate sensing and metabolism. Mutants of NLP7 show features of nitrogen-starved plants and are tolerant to drought stress. Localized in the nucleus and functions as a putative transcription factor. The mRNA is cell-to-cell mobile.
AT1G46768	1	encodes a member of the DREB subfamily A-5 of ERF/AP2 transcription factor family (RAP2.1). The protein contains one AP2 domain. There are 16 members in this subfamily including RAP2.9 and RAP2.10.
AT1G22190	1	The gene encodes a putative transcription factor belongings to the abiotic stress-associated DREB A-6 clade. The mRNA is cell-to-cell mobile.
AT1G78080	1	Encodes a member of the DREB subfamily A-6 of ERF/AP2 transcription factor family (RAP2.4). The protein contains one AP2 domain. Role in mediating light and ethylene signaling. The mRNA is cell-to-cell mobile.
AT5G13330	1	encodes a member of the ERF (ethylene response factor) subfamily B-4 of ERF/AP2 transcription factor family. The protein contains one AP2 domain. There are 7 members in this subfamily.
AT2G38470	1	Member of the plant WRKY transcription factor family. Regulates the antagonistic relationship between defense pathways mediating responses to P. syringae and necrotrophic fungal pathogens. Located in nucleus. Involved in response to various abiotic stresses - especially salt stress. Regulates cytochrome P450 gene CYP94B1 to control apoplastic barrier formation in roots to confer salt tolerance.

Table SEP5. TF's in Groups (G) 2: n(TF) = 8, L1201D and 3: n(TF) = 8, L1311H

Locus	G	Description
AT3G02160	2	Bromodomain transcription factor
AT2G38340	2	encodes a member of the DREB subfamily A-2 of ERF/AP2 transcription factor family. The protein contains one AP2 domain. There are eight members in this subfamily including DREB2A AND DREB2B that are involved in response to drought.
AT2G47190	2	Encodes a MYB transcription factor that possesses an R2R3 MYB DNA binding domain and is known to regulate the expression of salt- and dehydration-responsive genes. Has been shown to bind calmodulin.

AT1G52890	2	encodes a NAC transcription factor whose expression is induced by drought, high salt, and abscisic acid. This gene binds to ERD1 promoter in vitro.
AT1G02220	2	NAC domain transcription factor which functions as a negative regulator of the TDIF-PXY module and fine-tunes TDIF signaling in vascular development. Controls the balance of xylem formation and cambial cell divisions.
AT1G43160	2	encodes a member of the ERF (ethylene response factor) subfamily B-4 of ERF/AP2 transcription factor family (RAP2.6). The protein contains one AP2 domain. There are 7 members in this subfamily.
AT1G69310	2	Encodes WRKY57, a member of the WRKY Transcription Factor. Activation of WRKY57 confers drought tolerance.
AT3G58710	2	member of WRKY Transcription Factor; Group II-e. Involved in thermomorphogenesis.
AT2G41070	3	Transcription factor homologous to ABI5. Regulates AtEm1 expression by binding directly at the AtEm1 promoter. Located in the nucleus and expressed during seed maturation in the cotyledons and later in the whole embryo.
AT4G34000	3	Encodes an ABA-responsive element-binding protein with similarity to transcription factors that is expressed in response to stress and abscisic acid.
AT4G25480	3	Encodes a member of the DREB subfamily A-1 of ERF/AP2 transcription factor family (CBF3). The protein contains one AP2 domain. There are six members in this subfamily, including CBF1, CBF2, and CBF3. This gene is involved in response to low temperature and abscisic acid.
AT1G51140	3	Encodes a basic helix-loop-helix-type transcription factor involved in photoperiodism flowering. Binds to the E-box cis-element in the CONSTANS (CO) promoter to regulate flowering. Interacts with CFL1 and along with CFLAP2 negatively regulates cuticle development. Binds to the potassium channel gene KAT1 as a dimer. The DNA-binding capacity is inhibited in response to ABA through phosphorylation-dependent monomerization.
AT3G50310	3	Encodes a member of MEKK subfamily. Target promoter of the male germline-specific transcription factor DUO1. Involved in osmotic stress response via regulation of MPK6 activity. It also plays an important role in regulating cell division and cell elongation in the primary root meristematic and elongation areas. Mutants show defects in root microtubule organization. It phosphorylates MPK18 and MKK3.It is a positive regulator of ABA-induced stomatal closure that acts by phosphorylating MKK5.
AT3G47600	3	Encodes a putative transcription factor (MYB94).
AT4G27410	3	Encodes a NAC transcription factor induced in response to desiccation. It is localized to the nucleus and acts as a transcriptional activator in ABA-mediated dehydration response.
AT4G06634	3	Encodes an ABA responsive C2H2-type zinc finger transcription factor with both transcriptional repression and activation domains, that binds a G-rich, 11-bp DNA-binding motif. YY1 binds to the promoter of ABR1 and disruption represses ABA- and salt-induced ABR1 expression.

Table SEP6 G = 4, n(TF) = 6, L1303D; G = 5, n(TF) = 5, WT01D and G = 6, n(TF) = 4, L1205H

Locus	G	Description
AT5G21960	4	encodes a member of the DREB subfamily A-5 of ERF/AP2 transcription factor family. The protein contains one AP2 domain. There are 15 members in this subfamily including RAP2.1, RAP2.9 and RAP2.10.
AT5G07690	4	Encodes a putative transcription factor (MYB29) that acts as a negative regulator of mitochondrial stress responses.
AT1G08810	4	putative transcription factor of the R2R3-MYB gene family. Transcript increases under conditions that promote stomatal opening (white and blue light, abi1-1 mutation) and decreases under conditions that trigger stomatal closure (ABA, desiccation,

		darkness), with the exception of elevated CO2. Expressed exclusively in guard cells of all tissues. It is required for light-induced opening of stomata. Mutant shows reduced stomatal aperture which helps to limit water loss during drought.
AT5G07700	4	Encodes a putative transcription factor (MYB76), which inhibits the accumulation of seed oil.
AT1G74430	4	Encodes a putative transcription factor (MYB95). The mRNA is cell-to-cell mobile.
AT4G14540	4	Component of Nuclear factor Y transcription factor.
AT2G36270	5	Encodes a member of the basic leucine zipper transcription factor family, involved in ABA signalling during seed maturation and germination. The Arabidopsis abscisic acid (ABA)-insensitive abi5 mutants have pleiotropic defects in ABA response, including decreased sensitivity to ABA inhibition of germination and altered expression of some ABA-regulated genes. Comparison of seed and ABA-inducible vegetative gene expression in wild-type and abi5-1 plants indicates that ABI5 regulates a subset of late embryogenesis-abundant genes during both developmental stages. Responsible for reducing cadmium uptake, mediated by interaction with MYB49 .
AT2G38880	5	Encodes a transcription factor from the nuclear factor Y (NF-Y) family, AtNF-YB1. Confers drought tolerance.
AT1G19210	5	encodes a member of the DREB subfamily A-5 of ERF/AP2 transcription factor family. The protein contains one AP2 domain. There are 15 members in this subfamily including RAP2.1, RAP2.9 and RAP2.10.
AT4G16750	5	encodes a member of the DREB subfamily A-4 of ERF/AP2 transcription factor family. The protein contains one AP2 domain. There are 17 members in this subfamily including TINY.
AT2G46680	5	encodes a putative transcription factor that contains a homeodomain closely linked to a leucine zipper motif. Transcript is detected in all tissues examined. Is transcriptionally regulated in an ABA-dependent manner and may act in a signal transduction pathway which mediates a drought response.
AT1G01250	6	encodes a member of the DREB subfamily A-4 of ERF/AP2 transcription factor family. The protein contains one AP2 domain. There are 17 members in this subfamily including TINY.
AT4G25490	6	Transcriptional activator that binds to the DRE/CRT regulatory element and induces COR (cold-regulated) gene expression increasing plant freezing tolerance. It encodes a member of the DREB subfamily A-1 of ERF/AP2 transcription factor family (CBF1). The protein contains one AP2 domain. There are six members in this subfamily, including CBF1, CBF2, and CBF3. This gene is involved in response to low temperature and abscisic acid.
AT5G67190	6	encodes a member of the DREB subfamily A-5 of ERF/AP2 transcription factor family. The protein contains one AP2 domain. There are 16 members in this subfamily including RAP2.1, RAP2.9 and RAP2.10.
AT3G20310	6	Encodes a member of the ERF (ethylene response factor) subfamily B-1 of ERF/AP2 transcription factor family (ATERF-7). The protein contains one AP2 domain. Phosphorylated by PKS3 in vitro. Involved in ABA-mediated responses. Acts as a repressor of GCC box-mediated transcription together with AtSin3 and HDA19.

Table SEP7 G=7, n(TF) = 3, L1211H; G = 8, n(TF) = 3, L1301H; G = 9, n(TF) = 3, WT11H; G = 10, n(TF) = 2, L1201H

Locus	G	Description
AT5G05410	7	Encodes a transcription factor that specifically binds to DRE/CRT cis elements (responsive to drought and low-temperature stress). Belongs to the DREB subfamily A-2 of ERF/AP2 transcription factor family (DREB2A). There are eight members in this subfamily including DREB2B. The protein contains one AP2 domain. Overexpression of transcriptional activation domain of DREB2A resulted in significant drought stress tolerance but only slight freezing tolerance in transgenic Arabidopsis plants. Microarray and RNA gel blot analyses revealed that DREB2A regulates

		expression of many water stress-inducible genes. The mRNA is cell-to-cell mobile.
AT2G23340	7	encodes a member of the DREB subfamily A-5 of ERF/AP2 transcription factor family. The protein contains one AP2 domain. There are 16 members in this subfamily including RAP2.1, RAP2.9 and RAP2.10.
AT5G11270	7	Encodes a homeodomain transcription factor involved in mediating resistance to infection by necrotrophic pathogens dependent on perception of jasmonic acid through COI1. Expressed in the nucleus. Downregulated upon fungal infection. Also involved in drought tolerance.
AT1G19490	8	Putative bZIP transcription factor. Expression is induced by drought and mutants are sensitive to drought.
AT5G62470	8	Encodes a R2R3 type Myb transcription factor whose expression is strongly induced by abscisic acid. Mediates abscisic acid signaling during drought stress response. Promotes seed fatty acid accumulation.
AT1G54160	8	Encodes a member of the CCAAT-binding transcription factor (CBF-B/NF-YA) family. Expression is upregulated in response to ABA and drought. This regulation appears to be mediated by MIR169A which is downregulated in response to drought. NFYA5 is a target of MIR169A. Loss of function mutations are hypersensitive to drought.
AT1G22985	9	encodes a member of the ERF (ethylene response factor) subfamily B-5 of ERF/AP2 transcription factor family. The protein contains one AP2 domain. There are 7 members in this subfamily.
AT3G11020	9	encodes a member of the DREB subfamily A-2 of ERF/AP2 transcription factor family (DREB2B). The protein contains one AP2 domain. There are eight members in this subfamily including DREB2A.
AT2G47520	9	encodes a member of the ERF (ethylene response factor) subfamily B-2 of ERF/AP2 transcription factor family. The protein contains one AP2 domain. There are 5 members in this subfamily including RAP2.2 AND RAP2.12. It plays a role in hypoxia-induced root slanting.
AT1G17950	10	R2R3-MYB transcription family. Absence of the MYB52 transcription factor is correlated with an increase in PME activity and a decrease in the degree of pectin methylesterification. Transcriptionally activates PECTIN METHYLESTERASE INHIBITOR6 (PMEI6), PMEI14, and SUBTILISIN-LIKE SER PROTEASE1.7 (SBT1.7) by binding to their promoters. MYB52 can bind to the same regulatory regions as the ERF4 transcriptional represssor.
AT1G15360	10	Encodes a member of the ERF (ethylene response factor) subfamily B-6 of ERF/AP2 transcription factor family. The protein contains one AP2 domain. There are 12 members in this subfamily including RAP2.11. This gene is involved in wax biosynthesis. Over-expression of the gene results in glossy leaf phenotype and increased drought tolerance. Two closely related genes, AT5G25390 and AT5G11190 have similar phenotypes when over-expressed. Strong expression levels in flowers. Binds to the promoter of LACS2.

Table SBP8. G = 11, n(TF) = 2, L1203D; G = 12, n(TF) = 2, WT05H and G = 13, n(TF) = 1, L1305H.

Locus	G	Description
AT2G41240	11	Encodes a member of the basic helix-loop-helix transcription factor family protein. Functions as a key regulator of iron-deficiency responses independent of the master regulator FIT. Likely regulates genes involved in the distribution of iron within the plant. Phosphatidylinositol 4-phosphate 5-kinase (PIP5K) enzyme family member.
AT1G28520	11	VOZ transcription factor which acts as positive regulator of several salt-responsive genes. Functionally redundant in salt stress with VOZ2.
AT1G45249	12	Leucine zipper transcription factor that binds to the abscisic acid (ABA)-responsive element (ABRE) motif in the promoter region of ABA-inducible genes. Enhances

		drought tolerance in vegetative tissues. Required for normal glucose response. Localized in the nucleus. Expressed constitutively in roots, leaf vascular tissues, and hydathodes or in all tissues under stress conditions. It Primes phosphorylated by a ABA-activated 42-KDa kinase. Overexpression of the phosphorylated active form of AREB1 expressed many ABA-inducible genes, such as RD29B, without ABA treatment.
AT1G77200	12	encodes a member of the DREB subfamily A-4 of ERF/AP2 transcription factor family. The protein contains one AP2 domain. There are 17 members in this subfamily including TINY.
AT1G12610	13	Encodes a member of the DREB subfamily A-1 of ERF/AP2 transcription factor family (DDF1). The protein contains one AP2 domain. There are six members in this subfamily, including CBF1, CBF2, and CBF3. Overexpression of this gene results in delayed flowering and dwarfism, reduction of gibberellic acid biosynthesis, and increased tolerance to high levels of salt. This gene is expressed in all tissues examined, but most abundantly expressed in upper stems. Overexpression of this gene is also correlated with increased expression of GA biosynthetic genes and RD29A (a cold and drought responsive gene). Under salt stress it induces the expression of GAOX7, which encodes ad C20-GA inhibitor.

References

Benjamini, Y., Hochberg, Y. (1995). Controlling the false discovery rate: a practical and powerful approach to multiple testing. J. R. Stat. Soc. Series B Stat. Methodol. 57, 289-300. https://doi.org/10.1111/j.2517-6161.1995.tb02031.x

DeLuca, D.S., Levin, J.Z., Sivachenko, A., Fennell, T., Nazaire, M.D., Williams, C., Reich, M., Winckler, W., Getz, G. (2012). RNA-SeQC: RNA- seq metrics for quality control and process optimization. Bioinformatics 28, 1530-1532. doi: 10.1093/bioinformatics/bts196.

Gene Ontology Consortium (2004) The gene ontology (GO) database and informatics resource. Nucleic Acids Res 32, D258-D261. doi: 10.1093/nar/gkh036.

R Core Team (2013). R: A language and environment for statistical computing. R Foundation for Statistical Computing, Vienna, Austria. URL http://www.r-project.org.

Robinson, M.D., McCarthy, D.J., Smyth, G.K. (2010). edgeR: a Bioconductor package for differential expression analysis of digital gene expression data. Bioinformatics 26, 139-140. doi: 10.1093/bioinformatics/btp616.
